# Methoxy and bromo scans on *N*-(5-methoxyphenyl) methoxybenzenesulphonamides reveal potent cytotoxic compounds, especially against the human breast adenocarcinoma MCF7 cell line

**DOI:** 10.1080/14756366.2021.1925265

**Published:** 2021-06-09

**Authors:** Myriam González, María Ovejero-Sánchez, Alba Vicente-Blázquez, Manuel Medarde, Rogelio González-Sarmiento, Rafael Peláez

**Affiliations:** a Laboratorio de Química Orgánica y Farmacéutica, Departamento de Ciencias Farmacéuticas, Facultad de Farmacia, Universidad de Salamanca, Salamanca, Spain; b Instituto de Investigación Biomédica de Salamanca (IBSAL), Hospital Universitario de Salamanca, Salamanca, Spain; c Centro de Investigación de Enfermedades Tropicales de la Universidad de Salamanca (CIETUS), Facultad de Farmacia, Universidad de Salamanca, Salamanca, Spain; d Unidad de Medicina Molecular, Departamento de Medicina, Facultad de Medicina, Universidad de Salamanca, Salamanca, Spain; e Laboratorio de Diagnóstico en Cáncer Hereditario, Centro de Investigación del Cáncer, Universidad de Salamanca-CSIC, Salamanca, Spain

**Keywords:** Sulphonamides, tubulin, antimitotic, structure–activity relationships, colchicine

## Abstract

Thirty seven *N*-(5-methoxyphenyl)-4-methoxybenzenesulphonamide with methoxy or/and bromo substitutions (series 1-4) and with different substituents on the sulphonamide nitrogen have been synthesised. 21 showed sub-micromolar cytotoxicity against HeLa and HT-29 human tumour cell lines, and were particularly effective against MCF7. The most potent series has 2,5-dimethoxyanilines, especially the 4-brominated compounds **23**–**25**. The active compounds inhibit microtubular protein polymerisation at micromolar concentrations, thus pointing at tubulin as the target. Co-treatment with the MDR inhibitor verapamil suggests that they are not MDR substrates. Compound **25** showed nanomolar antiproliferative potency. It severely disrupts the microtubule network in cells and arrests cells at the G_2_/M cell-cycle phase, thus confirming tubulin targeting. **25** triggered apoptotic cell death, and induced autophagy. Docking studies suggest binding in a distinct way to the colchicine site. These compounds are promising new antitumor agents acting on tubulin.

## Graphical Abstract

**Figure:**
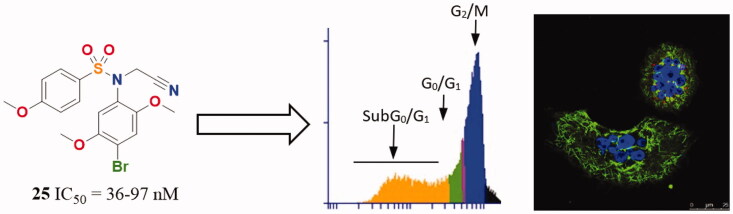

## Introduction

Microtubule dynamics is a very well-established target in antitumor, antiparasitic, herbicidal, and antifungal chemotherapy^1^^,^^2^. Many microtubule-targeting drugs act by binding to tubulin, with some of them causing microtubule destabilisation (MDA, such as colchicine or vincristine), and others stabilisation (MSA, such as the taxanes) through binding to several binding sites^3^. The colchicine domain of tubulin is a highly hydrophobic region located at the intradimer interface between the α- and β-tubulins^4^, and ligand binding prevents the dimer transition from curved to straight, which is necessary for polymerization^5^. Several colchicine-site ligands have reached clinical use as antiparasitic and antitumor drugs, such as the benzimidazoles nocodazole and albendazole^6^, the stilbene combretastatin A-4 (CA-4)^7^, or the sulphonamides ABT-751 or T138067^3^. However, each class of colchicine site drugs has liabilities, such as the low aqueous solubility and the instability of combretastatins^7^, the insufficient potency of ABT-751^3^, or the development of resistance by cancer cells^8^, which urge for new medicinal chemistry efforts.

Diarylsulphonamides are privileged scaffolds in medicinal chemistry and well-known anticancer agents^3^, with benzenesulphonamides carrying 3,4,5-trimethoxyphenyl (TMP) rings acting as tubulin inhibitors, such as *N*-(3,4,5-trimethoxyphenyl)-4-methoxybenzenesulphonamides^3^. The TMP ring has long been considered important for strong tubulin-binding through interactions with the sidechain of Cys241β amongst others and potent cytotoxic effects^4^^,^^9^^,^^10^, but it is also a bulky hydrophobic moiety that lowers the aqueous solubility and suffers from metabolic inactivation by *O*-demethylation reactions of the aromatic methoxy groups^11–13^. Recently, successful substitutions of the TMP ring have appeared in several tubulin inhibitory structural families, such as the combretastatins, the isocombretastatins, and the phenstatins, some of them even featuring polar azines replacing the phenyl ring^14–17^.

**Figure 1.**
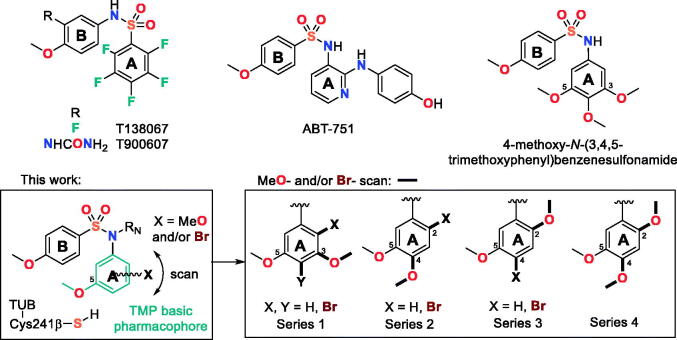
Chemical structure of tubulin inhibitor sulphonamides and summary of diarylsulphonamide scaffolds proposed in this work.

Here, we have explored new replacements for the *N*-TMP ring (Figure 1): keeping the 5-methoxy group to preserve the polar interaction with the sidechain of Cys241β and exploring the effect of the addition of methoxy groups (methoxy scan) and/or of bromine atoms (bromo scan) at different positions of the phenyl ring, as similar in size groups but with different bonding characteristics (weak hydrogen bonding acceptors for the methoxy groups and halogen bonding for the bromines^18^^,^^19^). We have combined these changes with different substituents on the sulphonamide nitrogen, exploring from hydrogen (unsubstituted) to small alkyls (methyl or ethyl) and more polar and larger acetates or acetonitriles or even to the much larger benzyl groups. We have assayed the new benzenesulphonamides against several human tumour cell lines with different sensitivity towards CA-4, against the non-tumorigenic cell line HEK293, and for tubulin inhibitory activity *in vitro*. We have also ascertained the sensitivity to MDR pumps by comparing the cytotoxicity of the compounds alone or combined with the MDR inhibitor verapamil^20^. The new family of compounds encompasses potent inhibitors of cell proliferation and tubulin polymerisation, in particular the 4-bromo-2,5-dimethoxyphenyl series. We have shown that the designed compounds are antitumor agents interfering with the mitotic machinery by studying their effect on the microtubules of cancer cells by immunofluorescence, and assessed the effects on the cell cycle by flow cytometry, showing initial G_2_/M arrest followed by apoptotic cell death. As for the reference ligands CA-4^21^ and ABT-751^22^, an increased autophagy response has been observed upon treatment. We have also studied the binding mode of the new benzenesulphonamides to the colchicine site of tubulin. These results show that the methoxy and bromo scans provide new antimitotic benzenesulphonamides devoid of the trimethoxyphenyl ring which are promising new antiproliferative agents.

## Materials and methods

### General chemical techniques

Reagents were used as purchased without further purification. Solvents (EtOAc, DMF, CH_2_Cl_2_, MeOH, CH_3_CN, toluene) were stored over molecular sieves. TLC was performed on precoated silica gel polyester plates (0.25 mm thickness) with a UV fluorescence indicator 254 (Polychrom SI F254). Chromatographic separations were performed on silica gel columns by flash (Kieselgel 40, 0.040–0.063; Merck) chromatography. Melting points were determined on a Büchi 510 apparatus and are uncorrected. ^1^H NMR and ^13 ^C NMR spectra were recorded in CDCl_3_ or CD_3_OD on a Bruker WP 200-SY spectrometer operating at 200/50 MHz, or Varian Mercury or Bruker SY spectrometers operating at 400/100 MHz. Chemical shifts (*δ*) are given in ppm downfield from tetramethylsilane and coupling constants (*J* values) are given in Hz. IR spectra were run on KBr discs on a Nicolet Impact 410 Spectrophotometer. A hybrid QSTAR XL quadrupole/time of flight spectrometer was used for HRMS analyses. GC-MS spectra were performed using a Hewlett-Packard 5890 series II mass detector. A Helios-α UV-320 from Thermo-Spectronic was used for UV spectra.

### General synthetic procedures

#### General procedure A for the formation of the sulphonamide bridge

To a stirred solution of the corresponding aniline (1 mmol) in CH_2_Cl_2_ (50 ml) and pyridine (2 ml), 4-methoxybenzenesulphonyl chloride (1 mmol) was slowly added. The mixture was stirred for 6 h at room temperature. Then the reaction was treated with 2 N HCl and 5% NaHCO_3_, washed with brine, dried over anhydrous Na_2_SO_4_ and the solvent evaporated to dryness. Crude reaction products were purified by crystallisation to afford the corresponding sulphonamides (**1**, **11**, **21,** and **32**).

#### General procedure B for alkylation of the sulphonamide nitrogen

*B1:* R_N_ = CH_3_. A mixture of the corresponding sulphonamide (1 mmol) and crushed KOH (2 mmol) in CH_3_CN (50 ml) was stirred for 30 min. Then, CH_3_I (2 mmol) was added and stirred at room temperature for 24 h. The solvent was removed under reduced pressure. The residue was re-dissolved in EtOAc and washed with brine, dried (Na_2_SO_4_), and concentrated in vacuum. Crude reaction products were further purified to afford the corresponding methylated sulphonamides (**2**, **12**, **14**, **22**, **24**, and **33**).

*B2:* R_N_ ≠ CH_3_. To a solution of the sulphonamide derivative (1 mmol) in dry DMF (3 ml), K_2_CO_3_ (2 mmol) was added and the mixture was stirred for 1 h at room temperature. Then, the appropriate alkyl halide (2 mmol) was added, and the mixture was stirred at room temperature for 24 h. The solution was concentrated in a vacuum and redissolved in EtOAc. Then, it was washed with brine, dried over Na_2_SO_4,_ and evaporated to dryness to give a crude reaction product which was further purified (**3**–**7**, **15**–**18**, **25**–**28,** and **34**–**37**).

#### General procedure C for bromination

A mixture of the corresponding sulphonamide (1 mmol) and *N*-bromosuccinimide (1–2 mmol) in CH_2_Cl_2_ (50 ml) was stirred at room temperature overnight. The reaction mixture was washed with brine, dried, and concentrated under a vacuum. Crude reaction products were further purified to afford the brominated sulphonamides (**8a**–**10c**, **13**, and **23**).

#### General procedure D for the nitration of Ar_A_ precursors

To a stirred solution at 0 °C of the corresponding Ar_A_ precursor (1 mmol) in acetic acid (5 ml), nitric acid (1 mmol) in acetic acid (5 ml) was dropwise added under nitrogen atmosphere. After 4 h, the reaction was poured onto ice and the mixture was kept at 4 °C for 30 min. Then, the precipitate was washed with cold water and filtered under vacuum to dryness. Crude reaction products were then used without further purification (**19** and **30**).

#### General procedure E for the reduction of nitro precursors

The nitro derivatives were dissolved in EtOAc. Pd(C) (10 mg) was added to the solution and stirred at room temperature under H_2_ atmosphere for 48 h. The reaction mixture was filtered through Celite^©^ and the solvent vacuum evaporated. Crude reaction products were then used without further purification (**20** and **31**).

### Chemical synthesis and characterisation

#### N-(3,5-dimethoxyphenyl)-4-methoxybenzenesulphonamide (1)

Following general procedure A, 3,5-dimethoxyaniline (290 mg, 1.89 mmol) and 4-methoxybenzenesulphonyl chloride (390 mg, 1.89 mmol) gave sulphonamide **1**. Yield, crude: 97% (596 mg); crystals: 71% (438 mg). M.p.: 115–122 °C (CH_2_Cl_2_/hexane). IR (KBr): 3234, 1595, 824 cm^−1^. ^1^H NMR (400 MHz, CDCl_3_): *δ* 3.66 (6H, s), 3.77 (3H, s), 6.13 (1H, t, *J* = 2), 6.17 (2H, d, *J* = 2), 6.85 (2H, d, *J* = 8.8), 7.68 (2H, d, *J* = 8.8). ^13^C NMR (100 MHz, CDCl_3_): *δ* 55.3 (2CH_3_), 55.5 (CH_3_), 97.0 (CH), 98.9 (2CH), 114.2 (2CH), 129.5 (2CH), 130.4 (C), 138.6 (C), 161.1 (2C), 163.1 (C). HRMS (C_15_H_17_NO_5_S + H^+^): calcd 324.0909 (M + H^+^), found 324.0900.

#### N-(3,5-dimethoxyphenyl)-4-methoxy-N-methylbenzenesulphonamide (2)

Following general procedure B1, compound **1** (205 mg, 0.63 mmol) was methylated with CH_3_I (79 µL, 1.27 mmol). Yield, crude: 89% (190 mg); crystals 50% (108 mg). M.p.: 120–128 °C (MeOH). ^1^H NMR (400 MHz, CDCl_3_): *δ* 3.10 (3H, s), 3.72 (6H, s), 3.85 (3H, s), 6.26 (2H, d, *J* = 2), 6.35 (1H, t, *J* = 2), 6.91 (2H, d, *J* = 9.2), 7.53 (2H, d, *J* = 9.2). ^13^C NMR (100 MHz, CDCl_3_): *δ* 38.1 (CH_3_), 55.4 (2CH_3_), 55.5 (CH_3_), 99.4 (CH), 104.9 (2CH), 113.8 (2CH), 128.2 (C), 129.9 (2CH), 143.5 (C), 160.5 (2C), 163.0 (C). HRMS (C_16_H_19_NO_5_S + H^+^): calcd 338.1057 (M + H^+^), found 338.1050.

#### N-(3,5-dimethoxyphenyl)-N-ethyl-4-methoxybenzenesulphonamide (3)

Following general procedure B2, compound **1** (274 mg, 0.85 mmol) was alkylated using bromoethane (252 µL, 3.40 mmol) as alkylating agent. Yield, crude: 87% (260 mg); crystals: 67% (201 mg). M.p.: 109–118 °C (MeOH). IR (KBr): 1605, 1497, 1162, 838 cm^−1^. ^1^H NMR (400 MHz, CDCl_3_): *δ* 1.07 (3H, t, *J* = 7.2), 3.52 (2H, q, *J* = 7.2), 3.71 (6H, s), 3.85 (3H, s), 6.20 (2H, d, *J* = 2), 6.39 (1H, t, *J* = 2), 6.93 (2H, d, *J* = 8.8), 7.58 (2H, d, *J* = 8.8). ^13^C NMR (100 MHz, CDCl_3_): *δ* 13.9 (CH_3_), 45.5 (CH_2_), 55.4 (2CH_3_), 55.5 (CH_3_), 100.1 (CH), 107.1 (2CH), 113.8 (2CH), 129.8 (2CH), 130.0 (C), 140.7 (C), 160.6 (2C), 162.8 (C). HRMS (C_17_H_21_NO_5_S + H^+^): calcd 352.1213 (M + H^+^), found 352.1219.

#### N-(cyanomethyl)-N-(3,5-dimethoxyphenyl)-4-methoxybenzenesulphonamide (4)

Following general procedure B2, compound **1** (113 mg, 0.35 mmol) was alkylated using 2-chloroacetonitrile (44 µL, 0.70 mmol) as alkylating agent. Yield, crude: 89% (113 mg); crystals: 53% (67 mg). M.p.: 92–96 °C (MeOH). ^1^H NMR (400 MHz, CDCl_3_): *δ* 3.70 (6H, s), 3.85 (3H, s), 4.51 (2H, s), 6.34 (2H, d, *J* = 2), 6.43 (1H, t, *J* = 2), 6.95 (2H, d, *J* = 8.8), 7.65 (2H, d, *J* = 8.8). ^13^C NMR (100 MHz, CDCl_3_): *δ* 39.4 (CH_2_), 55.5 (2CH_3_), 55.6 (CH_3_), 101.2 (CH), 106.1 (2CH), 114.2 (2CH), 115.0 (C), 128.8 (C), 130.2 (2CH), 140.2 (C), 161.1 (2C), 163.7 (C). HRMS (C_17_H_18_N_2_O_5_S + H^+^): calcd 363.1009 (M + H^+^), found 363.1003.

#### Ethyl N-(3,5-dimethoxyphenyl)-N-((4-methoxyphenyl)sulphonyl)glycinate (5)

Following general procedure B2, compound **1** (110 mg, 0.34 mmol) was alkylated using ethyl 2-bromoacetate (76 µL, 0.68 mmol) as alkylating agent and purified by preparative TLC (hexane:EtOAc 1:1). Yield, crude: 97% (135 mg); preparative: 80% (112 mg). ^1^H NMR (400 MHz, CDCl_3_): *δ* 1.22 (3H, t, *J* = 7.6), 3.68 (6H, s), 3.83 (3H, s), 4.14 (2H, q, *J* = 7.6), 4.35 (2H, s), 6.35 (3H, bs), 6.90 (2H, d, *J* = 8.8), 7.66 (2H, d, *J* = 8.8). ^13^C NMR (100 MHz, CDCl_3_): *δ* 14.0 (CH_3_), 52.6 (CH_2_), 55.3 (2CH_3_), 55.5 (CH_3_), 61.4 (CH_2_), 100.3 (CH), 106.4 (2CH), 113.8 (2CH), 130.0 (2CH), 130.4 (C), 141.7 (C), 160.7 (2C), 163.0 (C), 168.8 (C). HRMS (C_19_H_23_NO_7_S + H^+^): calcd 410.1268 (M + H^+^), found 410.1262.

#### N-benzyl-N-(3,5-dimethoxyphenyl)-4-methoxybenzenesulphonamide (6)

Following general procedure B2, compound **1** (90 mg, 0.28 mmol) was alkylated using benzyl chloride (48.5 µL, 0.42 mmol) as alkylating agent. Yield, crude: 90% (104 mg); crystals: 70% (81 mg). M.p.: 146–150 °C (MeOH). IR (KBr): 3467, 1458, 806 cm^−1^. ^1^H NMR (400 MHz, CDCl_3_): *δ* 3.62 (6H, s), 3.87 (3H, s), 4.65 (2H, s), 6.12 (2H, d, *J* = 2), 6.28 (1H, t, *J* = 2), 6.94 (2H, d, *J* = 8.8), 7.22 (5H, m), 7.63 (2H, d, *J* = 8.8). ^13^C NMR (100 MHz, CDCl_3_): *δ* 54.7 (CH_2_), 55.3 (2CH_3_), 55.6 (CH_3_), 100.0 (CH), 107.2 (2CH), 113.9 (2CH), 127.5 (CH), 128.3 (2CH), 128.5 (2CH), 129.8 (2CH), 130.3 (C), 136.1 (C), 140.9 (C), 160.4 (2C), 162.9 (C). HRMS (C_22_H_23_NO_5_S + H^+^): calcd 414.1370 (M + H^+^), found 414.1369.

#### Benzyl (3,5-dimethoxyphenyl)((4-methoxyphenyl)sulphonyl)carbamate (7)

Following general procedure B2, compound **1** (77 mg, 0.24 mmol) was alkylated using benzyl chloroformate (70 µL, 0.48 mmol) as alkylating agent and purified by preparative TLC (hexane:EtOAc 6:4). Yield 23% (25 mg). M.p.: 129–131 °C (MeOH). ^1^H NMR (400 MHz, CDCl_3_): *δ* 3.78 (6H, s), 3.88 (3H, s), 5.11 (2H, s), 6.40 (2H, d, *J* = 2.4), 6.53 (1H, t, *J* = 2.4), 6.92 (2H, d, *J* = 8.8), 7.16 (2H, bs), 7.29 (3H, bs), 7.90 (2H, d, *J* = 8.8). ^13^C NMR (100 MHz, CDCl_3_): *δ* 56.3 (2CH_3_), 56.5 (CH_3_), 69.5 (CH_2_), 102.5 (CH), 108.9 (2CH), 114.7 (2CH), 128.7 (2CH), 129.2 (CH), 129.3 (2CH), 131.2 (C), 132.1 (2CH), 135.6 (C), 138.2 (C), 152.8 (C), 161.6 (2C), 164.6 (C). HRMS (C_23_H_23_NO_7_S + Na^+^): calcd 480.1087 (M + Na^+^), found 480.1074.

#### N-(4-bromo-3,5-dimethoxyphenyl)-4-methoxy-N-methylbenzenesulphonamide (8a) and N-(2-bromo-3,5-dimethoxyphenyl)-4-methoxy-N-methylbenzenesulphonamide (8b)

Following general procedure C, compound **2** (190 mg, 0.56 mmol) was brominated. *N*-bromosuccinimide (150 mg, 0.84 mmol). **8a** and **8b** were isolated by flash column chromatography (hexane:EtOAc 8:2). Yield, **8a:** 54% (127 mg); **8b**: 14% (34 mg). **8a**: M.p.: 156–160 °C (MeOH). ^1^H NMR (200 MHz, CDCl_3_): *δ* 3.15 (3H, s), 3.77 (6H, s), 3.86 (3H, s), 6.30 (2H, s), 6.93 (2H, d, *J* = 8.8), 7.53 (2H, d, *J* = 8.8). ^13^C NMR (100 MHz, CDCl_3_): *δ* 38.2 (CH_3_), 55.6 (CH_3_), 56.5 (2CH_3_), 99.9 (C), 103.6 (2CH), 113.9 (2CH), 127.9 (C), 130.1 (2CH), 142.1 (C), 156.8 (2 C), 163.1 (C). HRMS (C_16_H_18_BrNO_5_S + H^+^): calcd 416.0162 and 418.0141 (M + H^+^), found 416.0158 and 418.0134. **8b**: M.p.: 158–164 °C (MeOH). IR (KBr): 1592, 1498, 1151, 666 cm^−1^. ^1^H NMR (200 MHz, CDCl_3_): *δ* 3.14 (3H, s), 3.73 (3H, s), 3.85 (3H, s), 3.87 (3H, s), 6.39 (1H, d, *J* = 2.6), 6.46 (1H, d, *J* = 2.6), 6.97 (2H, d, *J* = 9), 7.79 (2H, d, *J* = 9). ^13^C NMR (100 MHz, CDCl_3_): *δ* 38.1 (CH_3_), 55.6 (CH_3_), 56.4 (CH_3_), 56.7 (CH_3_), 99.8 (CH), 105.4 (C), 106.7 (CH), 114.1 (2CH), 130.1 (2CH), 130.7 (C), 141.7 (C), 157.5 (C), 159.6 (C), 163.0 (C). HRMS (C_16_H_18_BrNO_5_S + Na^+^): 437.9981 and 439.9961 calcd (M + Na^+^), found 437.9976 and 439.9961.

#### N-(4-bromo-3,5-dimethoxyphenyl)-N-ethyl-4-methoxybenzenesulphonamide (9a) and N-(2-bromo-3,5-dimethoxyphenyl)-N-ethyl-4-methoxybenzenesulphonamide (9b)

Following general procedure C, compound **3** (190 mg, 0.54 mmol) was brominated. *N*-bromosuccinimide (192 mg, 1.08 mmol). **9a** and **9b** were isolated by flash column chromatography (hexane:EtOAc 8:2). Yield, **9a:** 38% (90 mg); **9b**: 16% (37 mg). **9a**: M.p.: 141–144 °C (MeOH). ^1^H NMR (200 MHz, CDCl_3_): *δ* 1.09 (3H, t, *J* = 7), 3.57 (2H, q, *J* = 7), 3.75 (6H, s), 3.86 (3H, s), 6.23 (2H, s), 6.93 (2H, d, *J* = 9), 7.58 (2H, d, *J* = 9). ^13^C NMR (100 MHz, CDCl_3_): *δ* 14.0 (CH_3_), 45.6 (CH_2_), 55.6 (CH_3_), 56.5 (2CH_3_), 100.6 (C), 105.7 (2CH), 113.8 (2CH), 129.7 (C), 129.9 (2CH), 139.4 (C), 156.9 (2 C), 163.0 (C). HRMS (C_17_H_20_BrNO_5_S + H^+^): calcd 430.0318 and 432.0298 (M + H^+^), found 430.0313 and 432.0289. **9b**: M.p.: 146–150 °C (MeOH). ^1^H NMR (200 MHz, CDCl_3_): *δ* 1.09 (3H, t, *J* = 7), 3.59 (2H, m), 3.73 (3H, s), 3.86 (3H, s), 3.87 (3H, s), 6.33 (1H, d, *J* = 2.6), 6.47 (1H, d, *J* = 2.6), 6.95 (2H, d, *J* = 9), 7.76 (2H, d, *J* = 9). ^13^C NMR (100 MHz, CDCl_3_): *δ* 13.7 (CH_3_), 45.9 (CH_2_), 55.5 (CH_3_), 55.6 (CH_3_), 56.4 (CH_3_), 99.9 (CH), 106.9 (C), 108.2 (CH), 113.9 (2CH), 130.1 (2CH), 131.5 (C), 139.2 (C), 157.5 (C), 159.4 (C), 162.9 (C). HRMS (C_17_H_20_BrNO_5_S + H^+^): calcd 430.0318 and 432.0298 (M + H^+^), found 430.0312 and 432.0281.

#### N-benzyl-N-(4-bromo-3,5-dimethoxyphenyl)-4-methoxybenzenesulphonamide (10a), N-benzyl-N-(2-bromo-3,5-dimethoxyphenyl)-4-methoxybenzenesulphonamide (10b) and N-benzyl-N-(2,4-dibromo-3,5-dimethoxyphenyl)-4-methoxybenzenesulphonamide (10c)

Following general procedure C, compound **6** (195 mg, 0.47 mmol) was brominated. *N*-bromosuccinimide (168 mg, 0.94 mmol). **10a**, **10b** and **10c** were isolated by flash column chromatography (hexane:EtOAc 8:2). Yield, **10a:** 50% (116 mg); **10b**: 10% (24 mg); **10c**: 2% (6 mg). **10a:** M.p.: 199–203 °C (MeOH). IR (KBr): 3435, 1589, 836 cm^−1^. ^1^H NMR (200 MHz, CDCl_3_): *δ* 3.65 (6H, s), 3.88 (3H, s), 4.68 (2H, s), 6.12 (2H, s), 6.97 (2H, d, *J* = 9), 7.22 (5H, bs), 7.65 (2H, d, *J* = 9). ^13^C NMR (100 MHz, CDCl_3_): *δ* 54.9 (CH_2_), 55.7 (CH_3_), 56.4 (2CH_3_), 100.5 (C), 105.8 (2CH), 114.0 (2CH), 127.8 (CH), 128.4 (2CH), 128.6 (2CH), 129.9 (C), 130.0 (2CH), 135.7 (C), 139.4 (C), 156.7 (2 C), 163.1 (C). HRMS (C_22_H_22_BrNO_5_S + H^+^): calcd 492.0475 and 494.0454 (M + H^+^), found 492.0473 and 494.0440. **10b:** IR (KBr): 2938, 1593, 831 cm^−1^. ^1^H NMR (200 MHz, CDCl_3_): *δ* 3.57 (3H, s), 3.80 (3H, s), 3.87 (3H, s), 4.60 (1H, d, *J* = 14.4), 4.89 (1H, d, *J* = 14.4), 6.14 (1H, d, *J* = 2.8), 6.38 (1H, d, *J* = 2.8), 6.94 (2H, d, *J* = 9), 7.20 (5H, bs), 7.74 (2H, d, *J* = 9). ^13^C NMR (100 MHz, CDCl_3_): *δ* 54.4 (CH_2_), 55.5 (CH_3_), 55.6 (CH_3_), 56.3 (CH_3_), 100.0 (CH), 105.9 (C), 109.4 (CH), 113.9 (2CH), 127.7 (CH), 128.2 (2CH), 129.4 (2CH), 130.1 (2CH), 131.8 (C), 135.7 (C), 138.8 (C), 157.2 (C), 158.9 (C), 163.0 (C). HRMS (C_22_H_22_BrNO_5_S + H^+^): calcd 492.0475 and 494.0454 (M + H^+^), found 492.0467 and 494.0447. **10c:** IR (KBr): 2935, 1595, 835 cm^−1^. ^1^H NMR (200 MHz, CDCl_3_): *δ* 3.60 (3H, s), 3.77 (3H, s), 3.87 (3H, s), 4.55 (1H, d, *J* = 14.4), 4.97 (1H, d, *J* = 14.4), 6.30 (1H, s), 6.95 (2H, d, *J* = 9), 7.22 (5H, m), 7.73 (2H, d, *J* = 9). ^13^C NMR (100 MHz, CDCl_3_): *δ* 54.2 (CH_2_), 55.6 (CH_3_), 56.5 (CH_3_), 60.5 (CH_3_), 108.8 (C), 112.4 (C), 112.8 (CH), 114.0 (2CH), 128.0 (CH), 128.3 (2CH), 129.4 (2CH), 130.0 (2CH), 131.5 (C), 135.4 (C), 137.4 (C), 155.6 (C), 155.7 (C), 163.2 (C). HRMS (C_22_H_21_Br_2_NO_5_S + H^+^): calcd 569.9580 and 571.9559 (M + H^+^), found 569.9577 and 571.9558.

#### N-(3,4-dimethoxyphenyl)-4-methoxybenzenesulphonamide (11)

Following general procedure A, 3,4-dimethoxyaniline (2.49 g, 16.26 mmol) and 4-methoxybenzenesulphonyl chloride (3.61 g, 16.26 mmol) gave sulphonamide **11**. Yield, crude: 99% (5.29 g); crystals: 75% (4.04 g). M.p.: 101–102 °C (CH_2_Cl_2_/hexane). IR (KBr): 3224, 1498, 801 cm^−1^. ^1^H NMR (400 MHz, CDCl_3_): *δ* 3.75 (3H, s), 3.79 (3H, s), 3.80 (3H, s), 6.53 (1H, dd, *J* = 8.8 and 2.8), 6.66 (1H, d, *J* = 8.8), 6.70 (1H, d, *J* = 2.8), 6.86 (2H, d, *J* = 8.8), 7.66 (2H, d, *J* = 8.8). ^13^C NMR (100 MHz, CDCl_3_): *δ* 55.5 (CH_3_), 55.9 (CH_3_), 55.9 (CH_3_), 107.7 (CH), 111.1 (CH), 114.0 (2CH), 115.4 (CH), 129.4 (2CH), 129.5 (C), 130.3 (C), 147.2 (C), 149.1 (C), 163.0 (C). HRMS (C_15_H_17_NO_5_S + H^+^): calcd 324.0900 (M + H^+^), found 324.0906.

#### N-(3,4-dimethoxyphenyl)-4-methoxy-N-methylbenzenesulphonamide (12)

Following procedure B1, compound **11** (205 mg, 0.63 mmol) was methylated and purified by silica gel column chromatography (hexane:EtOAc, 6:4). CH_3_I (79 µL, 1.27 mmol). Yield 84% (180 mg). IR (KBr): 3435, 1595, 1508, 823 cm^−1^. ^1^H NMR (400 MHz, CDCl_3_): *δ* 3.06 (3H, s), 3.72 (3H, s), 3.80 (3H, s), 6.46 (1H, dd, *J* = 8.8 and 2.0), 6.62 (1H, d, *J* = 2.0), 6.69 (1H, d, *J* = 8.8), 6.87 (2H, d, *J* = 9.2), 7.44 (2H, d, *J* = 9.2). ^13^C NMR (100 MHz, CDCl_3_): *δ* 38.5 (CH_3_), 55.6 (CH_3_), 55.8 (CH_3_), 55.9 (CH_3_), 110.5 (CH), 111.1 (CH), 113.8 (2CH), 118.5 (CH), 127.8 (C), 130.0 (2CH), 134.5 (C), 148.2 (C), 148.6 (C), 162.9 (C). HRMS (C_16_H_19_NO_5_S + H^+^): calcd 338.1057 (M + H^+^), found 338.1058.

#### N-(2-bromo-4,5-dimethoxyphenyl)-4-methoxybenzenesulphonamide (13)

Following general procedure C, compound **11** (195 mg, 0.60 mmol) was brominated. *N*-bromosuccinimide (129 mg, 0.72 mmol). Yield, crude: 95% (232 mg); crystals: 32% (77 mg). M.p.: 162–167 °C (CH_2_Cl_2_/hexane). IR (KBr): 3265, 1594, 1510, 895 cm^−1^. ^1^H NMR (400 MHz, CDCl_3_): *δ* 3.80 (3H, s), 3.82 (3H, s), 3.89 (3H, s), 6.59 (1H, s), 6.81 (1H, s), 6.86 (2H, d, *J* = 9.2), 7.62 (2H, d, *J* = 9.2). ^13^C NMR (100 MHz, CDCl_3_): *δ* 55.5 (CH_3_), 56.1 (2CH_3_), 107.1 (C), 108.1 (CH), 114.0 (2CH), 114.3 (CH), 127.6 (C), 129.5 (2CH), 130.1 (C), 147.4 (C), 148.8 (C), 163.2 (C). HRMS (C_15_H_16_BrNO_5_S + H^+^): calcd 402.0005 and 403.9985 (M + H^+^), found 402.0008 and 403.9972.

#### N-(2-bromo-4,5-dimethoxyphenyl)-4-methoxy-N-methylbenzenesulphonamide (14)

Following procedure B1, compound **13** (54 mg, 0.13 mmol) was methylated. CH_3_I (16 µL, 0.26 mmol). Yield, crude: 88% (49 mg); crystals: 52% (29 mg). M.p.: 136–141 °C (MeOH). IR (KBr): 1596, 1507, 833 cm^−1^. ^1^H NMR (400 MHz, CDCl_3_): *δ* 3.15 (3H, s), 3.72 (3H, s), 3.84 (3H, s), 3.85 (3H, s), 6.62 (1H, s), 6.95 (2H, d, *J* = 9.2), 6.99 (1H, s), 7.73 (2H, d, *J* = 9.2). ^13^C NMR (100 MHz, CDCl_3_): *δ* 38.3 (CH_3_), 55.6 (CH_3_), 56.0 (CH_3_), 56.2 (CH_3_), 113.0 (CH), 113.9 (2CH), 114.7 (C), 115.4 (CH), 130.1 (2CH), 130.6 (C), 132.4 (C), 148.4 (C), 149.4 (C), 163.0 (C). HRMS (C_16_H_18_BrNO_5_S + H^+^): calcd 416.0162 and 418.0141 (M + H^+^), found 416.0168 and 418.0155.

#### N-(2-bromo-4,5-dimethoxyphenyl)-N-ethyl-4-methoxybenzenesulphonamide (15)

Following general procedure B2, compound **13** (91 mg, 0.22 mmol) was alkylated using bromoethane (34 µL, 0.45 mmol) as alkylating agent. Yield, crude: 72% (71 mg); crystals: 39% (38 mg). M.p.: 111–117 °C (MeOH). ^1^H NMR (200 MHz, CDCl_3_): *δ* 1.10 (3H, t, *J* = 7.2), 3.49 (2H, m), 3.74 (3H, s), 3.87 (6H, s), 6.57 (1H, s), 6.95 (2H, d, *J* = 9.0), 7.03 (1H, s), 7.72 (2H, d, *J* = 9.0). ^13^C NMR (100 MHz, CDCl_3_): *δ* 13.8 (CH_3_), 45.9 (CH_2_), 55.6 (CH_3_), 56.0 (CH_3_), 56.2 (CH_3_), 113.9 (2CH), 114.1 (CH), 115.5 (CH), 116.4 (C), 129.8 (C), 130.0 (2CH), 131.3 (C), 148.2 (C), 149.4 (C), 162.9 (C). HRMS (C_17_H_20_BrNO_5_S + H^+^): calcd 430.0318 and 432.0298 (M + H^+^), found 430.0313 and 432.0285.

#### N-(2-bromo-4,5-dimethoxyphenyl)-N-(cyanomethyl)-4-methoxybenzenesulphonamide (16)

Following general procedure B2, compound **13** (103 mg, 0.25 mmol) was alkylated using 2-chloroacetonitrile (32.5 µL, 0.51 mmol) as alkylating agent. Yield, crude: 83% (94 mg); crystals: 51% (58 mg). M.p.: 76–84 °C (MeOH). ^1^H NMR (400 MHz, CDCl_3_): *δ* 3.72 (3H, s), 3.85 (6H, s), 4.21 (1H, bd), 4.88 (1H, bd), 6.74 (1H, s), 6.97 (2H, d, *J* = 9.2), 7.02 (1H, s), 7.72 (2H, d, *J* = 9.2). ^13^C NMR (100 MHz, CDCl_3_): *δ* 38.8 (CH_2_), 55.7 (CH_3_), 56.1 (CH_3_), 56.3 (CH_3_), 114.0 (CH), 114.3 (2CH), 115.0 (C), 115.2 (C), 115.5 (CH), 128.5 (C), 129.9 (C), 130.3 (2CH), 148.6 (C), 150.4 (C), 163.7 (C). HRMS (C_17_H_17_BrN_2_O_5_S + H^+^): calcd 441.0114 and 443.0094 (M + H^+^), found 441.0101 and 443.0091.

#### Ethyl N-(2-bromo-4,5-dimethoxyphenyl)-N-((4-methoxyphenyl)sulphonyl)glycinate (17)

Following general procedure B2, compound **13** (98 mg, 0.24 mmol) was alkylated using ethyl 2-bromoacetate (54 µL, 0.48 mmol) as alkylating agent. Yield, crude: 87% (104 mg); crystals: 44% (52 mg). M.p.: 116–126 °C (MeOH). IR (KBr): 1751, 1739, 1596, 1507, 831 cm^−1^. ^1^H NMR (200 MHz, CDCl_3_): *δ* 1.25 (3H, t, *J* = 7), 3.76 (3H, s), 3.84 (3H, s), 3.85 (3H, s), 4.16 (4H, m), 6.91 (2H, d, *J* = 9.2), 6.93 (1H, s), 7.12 (1H, s), 7.66 (2H, d, *J* = 9.2). ^13^C NMR (100 MHz, CDCl_3_): *δ* 14.1 (CH_3_), 51.9 (CH_2_), 55.5 (CH_3_), 56.0 (CH_3_), 56.2 (CH_3_), 61.3 (CH_2_), 113.8 (2CH), 114.6 (C), 115.0 (CH), 116.7 (CH), 130.0 (2CH), 130.1 (C), 131.7 (C), 148.0 (C), 149.7 (C), 163.1 (C), 169.3 (C). HRMS (C_19_H_22_BrNO_7_S + H^+^): calcd 488.0373 and 490.0353 (M + H^+^), found 488.0364 and 490.0346.

#### N-benzyl-N-(2-bromo-4,5-dimethoxyphenyl)-4-methoxybenzenesulphonamide (18)

Following general procedure B2, compound **13** (107 mg, 0.26 mmol) was alkylated using benzyl chloride (62 µL, 0.53 mmol) as alkylating agent. Yield, crude: 82% (107 mg); crystals: 26% (34 mg). M.p.: 114–120 °C (MeOH). ^1^H NMR (400 MHz, CDCl_3_): *δ* 3.54 (3H, s), 3.79 (3H, s), 3.83 (3H, s), 4.57 (1H, d, *J* = 14), 4.86 (1H, d, *J* = 14), 6.33 (1H, s), 6.89 (1H, s), 6.93 (2H, d, *J* = 8.4), 7.19 (5H, bs), 7.71 (2H, d, *J* = 8.4). ^13^C NMR (100 MHz, CDCl_3_): *δ* 54.8 (CH_2_), 55.9 (CH_3_), 56.2 (CH_3_), 56.4 (CH_3_), 114.3 (2CH), 115.6 (CH), 115.8 (C), 116.1 (CH), 128.2 (CH), 128.6 (2CH), 129.8 (C), 129.9 (2CH), 130.4 (2CH), 132.0 (C), 136.0 (C), 148.2 (C), 149.6 (C), 163.4 (C). HRMS (C_22_H_22_BrNO_5_S + H^+^): calcd 492.0475 and 494.0454 (M + H^+^), found 492.0469 and 494.0451.

#### 1,4-Dimethoxy-2-nitrobenzene (19)

Following general procedure D, 1,4-dimethoxybenzene (2.35 g, 17 mmol) was nitrated. HNO_3_ (1.13 ml, 17 mmol). Yield 94% (2.92 g). M.p.: 71.8–72.5 °C (CH_2_Cl_2_/hexane). IR (KBr): 1528, 874, 763 cm^−1^. ^1^H NMR (400 MHz, CDCl_3_): *δ* 3.82 (3H, s), 3.92 (3H, s), 7.03 (1H, d, *J* = 9.6), 7.12 (1H, dd, *J* = 9.6 and 3.2), 7.4 (1H, d, *J* = 3.2). ^1 ^C NMR (100 MHz, CDCl_3_): *δ* 55.9 (CH_3_), 56.9 (CH_3_), 109.9 (CH), 115.0 (CH), 120.8 (CH), 139.3 (C), 147.3 (C), 152.7 (C). GC-MS (C_8_H_9_NO_4_): 183 (M^+^).

#### 2,5-Dimethoxyaniline (20)

Following general procedure E, compound **19** (2.92 g, 15.95 mmol) was reduced. Yield 99% (2.42 g). IR (KBr): 3459, 1519, 839 cm^−1^. ^1^H NMR (400 MHz, CDCl_3_): *δ* 3.72 (3H, s), 3.79 (3H, s), 6.24 (1H, dd, *J* = 9.2 and 3.2), 6.33 (1H, d, *J* = 3.2), 6.69 (1H, d, *J* = 9.2). ^13^C NMR (100 MHz, CDCl_3_): *δ* 54.5 (CH_3_), 55.1 (CH_3_), 100.9 (CH), 101.1 (CH), 110.3 (CH), 136.2 (C), 140.9 (C), 153.3 (C). GC-MS (C_8_H_11_NO_2_): 153 (M^+^).

#### N-(2,5-dimethoxyphenyl)-4-methoxybenzenesulphonamide (21)

Following general procedure A, aniline **20** (2.42 g, 15.84 mmol) and 4-methoxybenzenesulphonyl chloride (3.27 g, 15.84 mmol) gave sulphonamide **21**. Yield, crude: 95% (4.9 g); crystals: 84% (4.29 g). M.p.: 114–115 °C (CH_2_Cl_2_/hexane). IR (KBr): 3313, 1578, 830 cm^−1^. ^1^H NMR (400 MHz, CDCl_3_): *δ* 3.62 (3H, s), 3.74 (3H, s), 3.81 (3H, s), 6.53 (1H, dd, *J* = 9.2 and 3.2), 6.65 (1H, d, *J* = 9.2), 6.86 (2H, d, *J* = 9.2), 7.01 (1H, bs), 7.14 (1H, d, *J* = 3.2), 7.72 (2H, d, *J* = 9.2). ^13^C NMR (100 MHz, CDCl_3_): *δ* 55.5 (CH_3_), 55.7 (CH_3_), 56.2 (CH_3_), 106.8 (CH), 109.5 (CH), 111.4 (CH), 113.9 (2CH), 126.8 (C), 129.4 (2CH), 130.7 (C), 143.4 (C), 153.8 (C), 163.0 (C). HRMS (C_15_H_17_NO_5_S + H^+^): calcd 324.0900 (M + H^+^), found 324.0900.

#### N-(2,5-dimethoxyphenyl)-4-methoxy-N-methylbenzenesulphonamide (22)

Following general procedure B1, compound **21** (615 mg, 1.90 mmol) was methylated. CH_3_I (242 µL, 3.80 mmol). Yield, crude: 95% (611 mg); crystals: 57% (366 mg). M.p.: 105–110 °C (CH_2_Cl_2_/hexane). IR (KBr): 3467, 2948, 1595, 818 cm^−1^. ^1^H NMR (400 MHz, CDCl_3_): *δ* 3.09 (3H, s), 3.32 (3H, s), 3.65 (3H, s), 3.76 (3H, s), 6.65 (1H, d, *J* = 8.8), 6.73 (1H, dd, *J* = 8.8 and 3.2), 6.78 (1H, d, *J* = 3.2), 6.84 (2H, d, *J* = 8.8), 7.54 (2H, d, *J* = 8.8). ^13^C NMR (100 MHz, CDCl_3_): *δ* 38.2 (CH_3_), 55.9 (CH_3_), 56.0 (CH_3_), 56.1 (CH_3_), 112.9 (CH), 114.1 (2CH), 114.9 (CH), 117.6 (CH), 130.0 (2CH), 131.5 (C), 151.0 (C), 153.6 (C), 163.1 (C). HRMS (C_16_H_19_NO_5_S + H^+^): calcd 338.1057 (M + H^+^), found 338.1059.

#### N-(4-bromo-2,5-dimethoxyphenyl)-4-methoxybenzenesulphonamide (23)

Following general procedure C, compound **21** (1.00 g, 3.09 mmol) was brominated. *N*-bromosuccinimide (550 mg, 3.09 mmol). Yield, crude: 94% (1.17 g), crystals: 68% (852 mg). M.p.: 118–119 °C (CH_2_Cl_2_/hexane). IR (KBr): 3238, 1597, 1503, 828 cm^−1^. ^1^H NMR (400 MHz, CDCl_3_): *δ* 3.59 (3H, s), 3.81 (3H, s), 3.84 (3H, s), 6.86 (2H, d, *J* = 8.8), 6.91 (1H, s), 6.97 (1H, s), 7.20 (1H, s), 7.67 (2H, d, *J* = 8.8). ^13^C NMR (100 MHz, CDCl_3_): *δ* 55.6 (CH_3_), 56.4 (CH_3_), 56.8 (CH_3_), 105.9 (CH), 106.3 (C), 114.0 (2CH), 115.9 (CH), 125.9 (C), 129.3 (2CH), 130.4 (C), 143.8 (C), 150.2 (C), 163.1 (C). HRMS (C_15_H_16_BrNO_5_S + H^+^): calcd 402.0005 and 403.9985 (M + H^+^), found 402.0006 and 403.9992.

#### N-(4-bromo-2,5-dimethoxyphenyl)-4-methoxy-N-methylbenzenesulphonamide (24)

Following general procedure B1, compound **23** (90 mg, 0.22 mmol) was methylated and purified by preparative TLC (hexane:EtOAc 7:3). CH_3_I (28 µL, 0.44 mmol). Yield, crude: 89% (83 mg); preparative: 82% (76 mg); crystals: 62% (60 mg). M.p.: 132–133 °C (CH_2_Cl_2_/hexane). IR (KBr): 1596, 1503, 833 cm^−1^. ^1^H NMR (400 MHz, CDCl_3_): *δ* 3.17 (3H, s), 3.37 (3H, s), 3.83 (3H, s), 3.86 (3H, s), 6.92 (2H, d, *J* = 8.8), 6.92 (1H, s), 7.61 (2H, d, *J* = 8.8). ^13^C NMR (100 MHz, CDCl_3_): *δ* 37.7 (CH_3_), 55.6 (2CH_3_), 56.8 (CH_3_), 111.4 (C), 113.6 (2CH), 115.9 (CH), 116.6 (CH), 128.6 (C), 129.6 (2CH), 130.8 (C), 149.6 (C), 150.5 (C), 162.7 (C). HRMS (C_16_H_18_BrNO_5_S + Na^+^): calcd 437.9981 and 439.9961 (M + Na^+^), found 437.9969 and 439.9944.

#### N-(4-bromo-2,5-dimethoxyphenyl)-N-(cyanomethyl)-4-methoxybenzenesulphonamide (25)

Following general procedure B2, compound **23** (308 mg, 0.76 mmol) was alkylated using 2-chloroacetonitrile (97 µL, 1.53 mmol) as alkylating agent. Yield, crude: 79% (306 mg); crystals: 65% (254 mg). M.p.: 296–297 °C (MeOH). ^1^H NMR (400 MHz, CDCl_3_): *δ* 3.40 (3H, s), 3.81 (3H, s), 3.84 (3H, s), 4.52 (2H, s), 6.91 (2H, d, *J* = 8.8), 6.93 (1H, s), 7.00 (1H, s), 7.58 (2H, d, *J* = 8.8). ^13^C NMR (100 MHz, CDCl_3_): *δ* 38.3 (CH_2_), 55.7 (2CH_3_), 56.8 (CH_3_), 113.2 (C), 113.9 (2CH), 115.3 (C), 116.1 (CH), 116.8 (CH), 124.8 (C), 129.9 (2CH), 130.0 (C), 150.0 (C), 150.0 (C), 163.4 (C). HRMS (C_17_H_17_BrN_2_O_5_S + Na^+^): calcd 462.9934 and 464.9913 (M + Na^+^), found 462.9942 and 464.9916.

#### Ethyl N-(4-bromo-2,5-dimethoxyphenyl)-N-((4-methoxyphenyl)sulphonyl)glycinate (26)

Following general procedure B2, compound **23** (248 mg, 0.61 mmol) was alkylated using ethyl 2-bromoacetate (137 µL, 1.23 mmol) as alkylating agent and purified by flash column chromatography (hexane:EtOAc 3:7). Yield, crude: 95% (286 mg); column: 89% (268 mg). ^1^H NMR (400 MHz, CDCl_3_): *δ* 1.16 (3H, t, *J* = 7.2), 3.31 (3H, s), 3.75 (3H, s), 3.76 (3H, s), 4.07 (2H, q, *J* = 7.2), 4.29 (2H, s), 6.83 (2H, d, *J* = 9.2), 6.90 (1H, s), 7.08 (1H, s), 7.52 (2H, d, *J* = 9.2). ^13^C NMR (100 MHz, CDCl_3_): *δ* 15.7 (CH_3_), 52.4 (CH_2_), 57.2 (CH_3_), 57.3 (CH_3_), 58.3 (CH_3_), 62.8 (CH_2_), 113.6 (C), 115.2 (2CH), 117.9 (CH), 119.2 (CH), 127.8 (C), 131.2 (2CH), 133.1 (C), 151.1 (C), 151.6 (C), 164.4 (C), 170.8 (C). HRMS (C_19_H_22_BrNO_7_S + H^+^): calcd 488.0373 and 490.0353 (M + H^+^), found 488.0367 and 490.0348.

#### Ethyl (4-bromo-2,5-dimethoxyphenyl)((4-methoxyphenyl)sulphonyl)carbamate (27)

Following general procedure B2, compound **23** (230 mg, 0.57 mmol) was alkylated using ethyl chloroformate (220 µL, 2.28 mmol) as alkylating agent and purified by flash column chromatography (CH_2_Cl_2_:hexane 98:2). Yield 31% (84 mg). ^1^H NMR (400 MHz, CDCl_3_): *δ* 1.10 (3H, t, *J* = 6.8), 3.64 (3H, s), 3.86 (3H, s), 3.87 (3H, s), 4.09 (2H, q, *J* = 6.8), 6.91 (1H, s), 6.96 (2H, d, *J* = 8.8), 7.14 (1H, s), 7.94 (2H, d, *J* = 8.8). ^13^C NMR (100 MHz, CDCl_3_): *δ* 14.0 (CH_3_), 55.6 (CH_3_), 55.9 (CH_3_), 56.9 (CH_3_), 63.3 (CH_2_), 113.0 (C), 113.4 (2CH), 115.7 (CH), 116.7 (CH), 124.1 (C), 130.4 (C), 131.5 (2CH), 149.9 (2 C), 151.9 (C), 163.7 (C). HRMS (C_18_H_20_BrNO_7_S + Na^+^): calcd 496.0036 and 498.0016 (M + Na^+^), found 496.0018 and 497.9997.

#### N-(4-bromo-2,5-dimethoxyphenyl)-4-methoxy-N-(oxiran-2-ylmethyl)benzenesulphonamide (28)

Following general procedure B2, compound **23** (139 mg, 0.35 mmol) was alkylated using epichlorohydrin (108 µL, 1.38 mmol) as alkylating agent and purified by preparative TLC (toluene:EtOAc 3:7). Yield 45% (71 mg). ^1^H NMR (400 MHz, CDCl_3_): *δ* 2.45 (1H, dd, *J* = 2.4), 2.71 (1H, bt), 3.17 (1H, m), 3.33 (3H, s), 3.73 (2H, bs), 3.84 (3H, s), 3.85 (3H, s), 6.91 (2H, d, *J* = 8.8), 6.96 (1H, s), 6.97 (1H, s), 7.59 (2H, d, *J* = 8.8). ^13^C NMR (100 MHz, CDCl_3_): *δ* 45.5 (CH_2_), 50.7 (CH_2_), 52.5 (CH), 55.5 (CH_3_), 55.6 (CH_3_), 56.8 (CH_3_), 112.1 (C), 113.6 (2CH), 116.5 (CH), 116.9 (CH), 126.7 (C), 129.6 (2CH), 131.4 (C), 149.8 (C), 150.2 (C), 162.8 (C). HRMS (C_18_H_20_BrNO_6_S + Na^+^): calcd 480.0087 and 482.0066 (M + Na^+^), found 480.0077 and 482.0048.

#### N-(4-bromo-2,5-dimethoxyphenyl)-N-((4-methoxyphenyl)sulphonyl)glycine (29)

Compound **26** (220 mg 0.45 mmol) was diluted with methanolic KOH for saponification. After 30 min stirring at room temperature, the solution was acidified with 2N HCl and extracted with EtOAc. The extract was washed with brine, dried over anhydrous Na_2_SO_4_ and evaporated to dryness. Yield, crude: 87% (181 mg); crystals: 68% (141 mg). M.p.: 180–184 °C (CH_2_Cl_2_/hexane). ^1^H NMR (400 MHz, CDCl_3_): *δ* 3.46 (3H, s), 3.79 (3H, s), 3.85 (3H, s), 4.35 (2H, s), 6.91 (2H, d, *J* = 9.2), 6.97 (1H, s), 7.00 (1H, s), 7.60 (2H, d, *J* = 9.2). ^13^C NMR (100 MHz, CDCl_3_): *δ* 51.0 (CH_2_), 55.6 (CH_3_), 55.7 (CH_3_), 56.8 (CH_3_), 112.4 (C), 113.7 (2CH), 116.5 (CH), 117.2 (CH), 126.1 (C), 129.7 (2CH), 131.0 (C), 149.8 (2C), 163.0 (C), 174.2 (C). HRMS (C_17_H_18_BrNO_7_S + Na^+^): calcd 481.9880 and 483.9859 (M + Na^+^), found 481.9876 and 483.9848.

#### 1,2,4-Trimethoxy-5-nitrobenzene (30)

Following general procedure D, 1,2,4-trimethoxybenzene (3.66 ml, 23.8 mmol) was nitrated. HNO_3_ (1.54 ml, 23.8 mmol). Yield 85% (4.30 g). ^1^H NMR (200 MHz, CDCl_3_): *δ* 3.89 (3H, s), 3.97 (3H, s), 6.56 (1H, s), 7.59 (1H, s). GC-MS (C_9_H_11_NO_5_): 213 (M^+^).

#### 2,4,5-Trimethoxyaniline (31)

Following general procedure E, compound **30** (3.20 g, 15.0 mmol) was reduced. Yield 99% (2.72 g). ^1^H NMR (200 MHz, CDCl_3_): *δ* 3.80 (3H, s), 3.81 (3H, s), 3.82 (3H, s), 6.39 (1H, s), 6.53 (1H, s). GC-MS (C_9_H_13_NO_3_): 183 (M^+^).

#### 4-Methoxy-N-(2,4,5-trimethoxyphenyl)benzenesulphonamide (32)

Following general procedure A, aniline **31** (465 mg, 2.54 mmol) and 4-methoxybenzenesulphonyl chloride (629 mg, 3.05 mmol) gave sulphonamide **32**. Yield, crude: 99% (893 mg); crystals: 65% (587 mg). M.p.: 112–114 °C (MeOH). ^1^H NMR (200 MHz, CDCl_3_): *δ* 3.49 (3H, s), 3.81 (6H, s), 3.85 (3H, s), 6.33 (1H, s), 6.52 (1H, bs), 6.84 (2H, d, *J* = 9), 7.16 (1H, s), 7.61 (2H, d, *J* = 9). ^13^C NMR (100 MHz, CDCl_3_): *δ* 55.5 (CH_3_), 56.2 (CH_3_), 56.3 (CH_3_), 56.5 (CH_3_), 97.2 (CH), 108.1 (CH), 113.7 (2CH), 117.9 (C), 129.4 (2CH), 130.6 (C), 143.1 (C), 144.8 (C), 147.0 (C), 162.8 (C). HRMS (C_16_H_19_NO_6_S + Na^+^): calcd 376.0825 (M + Na^+^), found 376.0825.

#### 4-Methoxy-N-methyl-N-(2,4,5-trimethoxyphenyl)benzenesulphonamide (33)

Following procedure B1, compound **32** (103 mg, 0.29 mmol) was methylated. CH_3_I (36 µL, 0.58 mmol). Yield, crude: 90% (97 mg); crystals: 70% (75 mg). M.p.: 137–143 °C (MeOH). IR (KBr): 1595, 1523, 1152, 893 cm^−1^. ^1^H NMR (200 MHz, CDCl_3_): *δ* 3.18 (3H, s), 3.43 (3H, s), 3.79 (3H, s), 3.86 (3H, s), 3.87 (3H, s), 6.38 (1H, s), 6.79 (1H, s), 6.93 (2H, d, *J* = 9), 7.64 (2H, d, *J* = 9). ^13^C NMR (100 MHz, CDCl_3_): *δ* 38.0 (CH_3_), 55.5 (CH_3_), 55.6 (CH_3_), 56.1 (CH_3_), 56.5 (CH_3_), 97.2 (CH), 113.5 (2CH), 114.9 (CH), 120.7 (C), 129.7 (2CH), 131.2 (C), 142.4 (C), 149.5 (C), 150.8 (C), 162.6 (C). HRMS (C_17_H_21_NO_6_S + Na^+^): calcd 390.0982 (M + Na^+^), found 390.0986.

#### N-ethyl-4-methoxy-N-(2,4,5-trimethoxyphenyl)benzenesulphonamide (34)

Following general procedure B2, compound **32** (102 mg, 0.29 mmol) was alkylated using bromoethane (43 µL, 0.58 mmol) as alkylating agent and purified by preparative TLC (hexane:EtOAc 1:1). Yield, crude: 95% (105 mg); preparative: 87% (96 mg). IR (KBr): 1594, 1504, 1110, 804 cm^−1^. ^1^H NMR (400 MHz, CDCl_3_): *δ* 1.01 (3H, t, *J* = 7.2), 3.39 (3H, s), 3.55 (2H, bs), 3.75 (3H, s), 3.80 (3H, s), 3.83 (3H, s), 6.36 (1H, s), 6.70 (1H, s), 6.87 (2H, d, *J* = 9.2), 7.59 (2H, d, *J* = 9.2). ^13^C NMR (100 MHz, CDCl_3_): *δ* 14.3 (CH_3_), 44.7 (CH_2_), 55.5 (CH_3_), 55.6 (CH_3_), 56.0 (CH_3_), 56.4 (CH_3_), 97.0 (CH), 113.4 (2CH), 116.3 (CH), 117.8 (C), 129.5 (2CH), 132.2 (C), 142.2 (C), 149.6 (C), 151.3 (C), 162.4 (C). HRMS (C_18_H_23_NO_6_S + Na^+^): calcd 404.1138 (M + Na^+^), found 404.1138.

#### N-(cyanomethyl)-4-methoxy-N-(2,4,5-trimethoxyphenyl)benzenesulphonamide (35)

Following general procedure B2, compound **32** (87 mg, 0.24 mmol) was alkylated using 2-chloroacetonitrile (31 µL, 0.49 mmol) as alkylating agent. Yield, crude: 72% (70 mg); crystals: 56% (55 mg). M.p.: 173–180 °C (MeOH). ^1^H NMR (200 MHz, CDCl_3_): *δ* 3.45 (3H, s), 3.79 (3H, s), 3.86 (3H, s), 3.88 (3H, s), 4.54 (2H, s), 6.38 (1H, s), 6.84 (1H, s), 6.93 (2H, d, *J* = 9.2), 7.63 (2H, d, *J* = 9.2). ^13^C NMR (100 MHz, CDCl_3_): *δ* 38.7 (CH_2_), 55.5 (CH_3_), 55.6 (CH_3_), 56.1 (CH_3_), 56.4 (CH_3_), 96.9 (CH), 113.8 (2CH), 115.3 (CH), 115.4 (C), 116.8 (C), 129.9 (2CH), 130.5 (C), 142.7 (C), 150.6 (C), 150.6 (C), 163.2 (C). HRMS (C_18_H_20_N_2_O_6_S + Na^+^): calcd 415.0934 (M + Na^+^), found 415.0934.

#### Ethyl N-((4-methoxyphenyl)sulphonyl)-N-(2,4,5-trimethoxyphenyl)glycinate (36)

Following general procedure B2, compound **32** (95 mg, 0.27 mmol) was alkylated using ethyl 2-bromoacetate (60 µL, 0.54 mmol) as alkylating agent. Yield, crude: 99% (117 mg); crystals: 69% (82 mg). M.p.: 127–132 °C (MeOH). ^1^H NMR (200 MHz, CDCl_3_): *δ* 1.24 (3H, t, *J* = 7.2), 3.43 (3H, s), 3.78 (3H, s), 3.84 (3H, s), 3.85 (3H, s), 4.15 (2H, q, *J* = 7.2), 4.38 (2H, s), 6.34 (1H, s), 6.90 (2H, d, *J* = 9), 7.02 (1H, s), 7.63 (2H, d, *J* = 9). ^13^C NMR (100 MHz, CDCl_3_): *δ* 14.1 (CH_3_), 51.4 (CH_2_), 55.5 (2CH_3_), 56.0 (CH_3_), 56.3 (CH_3_), 61.1 (CH_2_), 96.6 (CH), 113.4 (2CH), 116.7 (CH), 118.3 (C), 129.8 (2CH), 132.0 (C), 142.2 (C), 149.8 (C), 150.3 (C), 162.7 (C), 169.6 (C). HRMS (C_20_H_25_NO_8_S + Na^+^): calcd 462.1193 (M + Na^+^), found 462.1193.

#### N-benzyl-4-methoxy-N-(2,4,5-trimethoxyphenyl)benzenesulphonamide (37)

Following general procedure B2, compound **32** (95 mg, 0.27 mmol) was alkylated using benzyl chloride (62.4 µL, 0.54 mmol) as alkylating agent. Yield, crude: 99% (118 mg); crystals: 82% (98 mg). M.p.: 130–139 °C (MeOH). IR (KBr): 1596, 1492, 1184, 809 cm^−1^. ^1^H NMR (200 MHz, CDCl_3_): *δ* 3.37 (3H, s), 3.62 (3H, s), 3.82 (3H, s), 3.87 (3H, s), 4.70 (2H, bs), 6.30 (1H, s), 6.49 (1H, s), 6.93 (2H, d, *J* = 9), 7.20 (5H, bs), 7.68 (2H, d, *J* = 9). ^13^C NMR (100 MHz, CDCl_3_): *δ* 53.7 (CH_2_), 55.5 (CH_3_), 55.6 (CH_3_), 55.9 (CH_3_), 56.3 (CH_3_), 96.7 (CH), 113.5 (2CH), 116.8 (CH), 117.9 (C), 127.4 (CH), 128.1 (2CH), 128.8 (2CH), 129.7 (2CH), 132.5 (C), 136.8 (C), 142.1 (C), 149.6 (C), 151.0 (C), 162.5 (C). HRMS (C_23_H_25_NO_6_S + Na^+^): calcd 466.1295 (M + Na^+^), found 466.1287.

### Aqueous solubility

The aqueous solubility was determined in a Helios-α UV-320 Spectrophotometer Thermo-Spectronic, Thermo Fischer Scientific, Waltham, MA, USA) using an approach based on the saturation shake-flask method. 1–2 mg of each selected compound was suspended in 300 µL pH 7.0 buffer and stirred for 48 h at room temperature. The resulting mixture was filtered through a 45 µm filter to discard the insoluble residues. A scan between 270 and 400 nm for the tested compound was performed and three wavelengths of maximum absorbance were selected. Calibration curves were performed at these wavelengths and the concentration in the supernatant was measured by UV absorbance. The final value was given as the average of the three measurements.

### Cell lines and cell culture conditions

MCF7 (human breast carcinoma), HeLa (human cervical carcinoma), and HEK-293 (human embryonic kidney) cells were cultured in Dulbecco’s Modified Eagle’s Medium (DMEM) (Gibco, Thermo Fischer Scientific) containing 10% (v/v) heat-inactivated foetal bovine serum (HIFBS) (Lonza-Cambrex, Karlskoga, Sweden), 2 mM L-glutamine (Lonza-Cambrex) and 100 μg/mL streptomycin-100 IU/mL penicillin (Lonza-Cambrex) at 37 °C in 95% humidified and 5% CO_2_ air. HT-29 (human colon carcinoma) cells were cultured in RPMI 1640 medium (Gibco) supplemented with 10% HIFBS and 100 μg/mL streptomycin-100 IU/mL penicillin at 37 °C in humidified 95% air and 5% CO_2_ atmosphere. The presence of mycoplasma was routinely checked with MycoAlert kit (Lonza-Cambrex) and only mycoplasma-free cells were used in the experiments. Tumour cell lines were kindly provided by Dr Faustino Mollinedo from Centro de Investigaciones Biológicas Margarita Salas, Madrid, Spain.

### Cell growth assay

To determine cell growth, cells in exponential growth phase were seeded (100 µL/well in 96-well plates) with appropriate cell concentration (1.5 × 10^4^ cells/mL for MCF7, HeLa and HEK-293 and 3 × 10^4^ cells/mL for HT-29) in complete DMEM or RPMI 1640 medium (see above) at 37 °C and 5% CO_2_ atmosphere. After 24 h incubation, to allow cells to attach to the plates, all compounds were added at 1 µM concentration (10 µL/well in 96-well plates) and the effect on the proliferation was evaluated 72 h post-treatment. Compounds showing antiproliferative effects at tested concentration were selected for IC_50_ calculation (50% inhibitory concentration compared to the untreated controls) from 10^−5^ to 10^−10 ^M concentration. Nonlinear curves fitting the experimental data were carried out for each compound. MCF7, HeLa, HT-29, and HEK-293 cell growth, when treated with the corresponding compound, were determined using the XTT (sodium 3′-[1(phenylaminocarbonyl)-3,4-tetrazolium]-bis(4-methoxy-6-nitro)-benzenesulphonic acid hydrate) cell proliferation kit (Roche Molecular Biochemicals, Mannheim, Germany). A freshly prepared mixture solution of XTT labelling reagent with 0.02% (v/v) PMS (N-methyl-dibenzopyrazine methyl sulfate) electron coupling reagent was added to cells (50 µL/well in 96-well plates, the total volume of 160 µL/well) and were incubated during the corresponding time according to each cell line (4 h for MCF7 and HeLa and 6 h for HT-29 and HEK-293) in a humidified atmosphere (37 °C, 5% CO_2_). The absorbance of the formazan product generated was measured at a test wavelength of 450 nm using a multi-well plate reader (Ultra Evolution, Tecan, Männedorf, Suiza). Compounds were dissolved in DMSO and the final solvent concentrations never exceeded 0.5% (v/v). The control wells included treated cells with 0.5% (v/v) DMSO and the positive control. 10 µM verapamil was included as a control for the HT-29 cell line. Measurements were performed in triplicate, and each experiment was repeated three times.

### Cell cycle analysis

MCF7, HeLa, and HT-29 cell cycle analyses were performed by quantifying the DNA content by flow cytometry. Cells in the exponential growth phase at 2 × 10^4^ cells/mL were seeded in 24-well plates (1 ml/well). After 24 h incubation, cells were treated with compound **25** at 87.5 or 175 nM. After 24, 48, and 72 h treatments, live and dead cells were collected and fixed in ice-cold ethanol:PBS (7:3) and stored at 4 °C for later use. Cells were rehydrated with phosphate-buffered saline (PBS), treated with 100 µg/mL RNase A (Sigma-Aldrich Co., St. Louis, MO, USA), and stained overnight in darkness at room temperature with 50 µg/mL propidium iodide (PI) (Sigma-Aldrich Co.). Cell cycle profiles were then analysed by flow cytometry using BD Accuri™ C6 Plus Flow Cytometer (BD Biosciences, San José, CA, USA). Data were analysed with BD Accuri™ C6 Software (version 1.0.264.21, BD Biosciences, San José, CA, USA) and compared to control cells treated with 0.5% (v/v) DMSO. Compounds were dissolved in DMSO and the final solvent concentrations never exceeded 0.5% (v/v).

### Apoptotic cell death quantification

MCF7, HeLa, and HT-29 apoptotic cells were quantified using an Annexin V-FITC/PI apoptosis detection kit (Immunostep, Salamanca, Spain) according to the manufacturer’s guidelines. 1.5 ml/well of cells in the exponential growth phase at 2 × 10^4^ cells/mL were seeded onto 12-well plates and left to attach overnight. After 24 h incubation, cells were treated with compound **25** at 87.5 or 175 nM. 72 h post-treatment, attached and floating cells of treated and untreated wells were collected, centrifuged, resuspended in the Annexin V binding buffer, and stained with Annexin V-FITC/PI. Cells were then incubated in darkness at room temperature for 15 min and a total of 30,000 cells were acquired and analysed using the BD Accuri™ C6 Plus Flow Cytometer and Software (version 1.0.264.21, BD Biosciences, San José, CA, USA), respectively. Compounds were dissolved in DMSO and the final solvent concentrations never exceeded 0.5% (v/v). Control wells included cells with 0.5% (v/v) DMSO.

### Western blotting

MCF7, HeLa and HT-29 cells in an exponential growth phase at 2 × 10^4^ cells/mL were seeded in Petri dishes (20 × 100 mm) (10 ml/dish). After 24 h incubation, cells were treated with compound **25** at 175 nM, and the levels of p-62 in treated and untreated cells were measured at 24 h post-treatment. The culture medium was removed, cells were washed with PBS and resuspended in 1 ml lysis buffer (50 mM Tris, 130 mM NaCl, 1 mM EDTA, 1% Triton X-100) containing protease inhibitors (Complete, Roche Applied Science, Indianapolis). Cells were harvested and incubated with lysis buffer for 30 min at 4 °C in constant agitation. Cell extracts were centrifuged at 12,000 rpm for 15 min at 4 °C and the supernatants were stored at −80 °C. Protein concentrations were determined using Bradford (BioRad, Hercules, CA) method taking BSA (Bovine Serum Albumin) as standard. Protein extracts were used for electrophoresis mixing with loading buffer (4% SDS, 0.05% bromophenol blue, 20% glycerine, 2% β-mercaptoethanol, and Tris 100 mM at pH 6.8). Proteins were then denatured at 95 °C for 7 min. Protein samples (50 μg/lane) were subjected to electrophoresis on 8% acrylamide gel and SDS-PAGE running buffer at constant voltage (140 V). Then, acrylamide gels were transferred to a PVDF membrane (Merck Millipore, Burlington, Massachusetts, USA) using a semi-dry transfer system for 30 min at constant voltage (15 V). After blocking with TBS-T (NaCl 140 mM, Tween20 0.05%, Tris 10 mM at pH 7.5) with 5% of skimmed milk powder, membranes were incubated overnight at 4 °C with p-62 (1:1000, ab109012, Abcam, Cambridge, UK) or β-actin (1:10000, Sigma-Aldrich Co.) antibodies, in 3% BSA/TBS-T. β-actin was used as the loading control. Immunoblots were incubated for 50 min at room temperature with secondary antibodies (Horseradish peroxidase linked-sheep (anti-mouse) (NXA931, GE Healthcare, Chicago, Illinois, USA) or goat (anti-rabbit) (AP307P, Merck Millipore)) at a 1:10,000 dilution in 7% of skimmed milk powder/TBS-T. Bands were visualised using an enhanced chemiluminescence western blotting detection kit (Thermo Fisher Scientific) based on the oxidation of luminol in the presence of hydrogen peroxide.

### Immunofluorescence

MCF7, HeLa, and HT-29 cells were grown on round glass coverslips coated with poly-L-lysine (12 mm diameter). To reach appropriate cell confluence, coverslips were manipulated in 6-well plates (3 coverslips/well) seeding 3 ml/well of cells in the exponential growth phase at 2 × 10^4^ cells/mL. After 24 h incubation, cells were treated with compound **25** at 175 nM and incubated for 72 h. Cells were washed with PBS, fixed with 4% formaldehyde in PBS for 10 min, permeabilized with 0.5% Triton X-100 (Boehringer Mannheim, Ingelheimam Rhein, Germany) in PBS for 10 min, and blocked with 10% BSA in PBS for 30 min. Then, coverslips were incubated with anti-α-tubulin mouse monoclonal antibody (diluted 1:200 in 3% BSA/PBS) (Sigma-Aldrich Co.) and anti-SQSTM1/p62 rabbit monoclonal antibody (diluted 1:100 in 3% BSA/PBS) (Abcam) for 1 h 30 min. After PBS washing, coverslips were incubated with fluorescent secondary antibodies Alexa Fluor 488 goat anti-mouse IgG and Alexa Fluor 594 anti-rabbit (both diluted 1:400 in 1% BSA/PBS) (Molecular Probes, Invitrogen, Thermo Fischer Scientific) for 1 h in darkness. After being washed with PBS, cell nuclei were stained with DAPI (dihydrochloride of 4′,6-diamidino-2-phenylindole) (diluted 1:10,000 in milli-Q H_2_O) (Roche, Basel, Switzerland) for 5 min in darkness, DAPI excess was removed by washing with PBS. Finally, Mowiol reagent (Calbiochem, Sigma-Aldrich Co.) was used to mount preparations on slides. Cells were analysed by confocal microscopy using a LEICA SP5 microscope DMI-6000V model coupled to a LEICA LAS AF software computer.

### Tubulin isolation and inhibition of tubulin polymerisation

Microtubular protein was isolated from calf brain according to the modified Shelanski method^23^^,^^24^ by two cycles of temperature-dependent assembly/disassembly and stored at −80 °C. Before each use, protein concentration is determined by Bradford method^25^ taking BSA as standard. Tubulin polymerisation was monitored using a Helios α spectrophotometer by measuring the increase in turbidity at 450 nm, caused by a shift from 4 °C to 37 °C, which allows the *in vitro* microtubular protein to depolymerise and to polymerise, respectively. The assay was carried out in quartz cuvettes containing 1.5 mg/mL microtubular protein and the ligand (except for control cuvette with only DMSO at the same concentration) in a mixture of 0.1 M MES buffer, 1 mM EGTA, 1 mM MgCl_2_, 1 mM β-ME, and 1.5 mM GTP at pH 6.7 (final volume 500 µL). Cuvettes were preincubated at 20 °C for 30 min, to allow ligand binding to tubulin, and subsequently cooled on ice for 10 min. Then, the experiment starts at 4 °C to establish the initial baseline. The assembly process was initiated by a temperature shift to 37 °C and the turbidity produced by tubulin polymerisation can be measured by an absorbance increase. After reaching a stable plateau, the temperature was switched back to 4 °C to return to the initial absorption values (to confirm the reversible nature of the monitored process and to determine whether or not the microtubular protein has stabilized). The difference in amplitude between the stable plateau and the initial baseline of the curves was taken as the degree of tubulin polymerisation for each experiment. Comparison with control curves under identical conditions but without ligands yielded TPI as a percentage value. All compounds were tested at 10 µM concentration. IC_50_ values were calculated for compounds that inhibit tubulin polymerisation more than 50% at 10 µM. Compounds were dissolved in DMSO and the final solvent concentrations never exceeded 4% (v/v), which has been reported not to interfere with the assembly process. All the measurements were carried out in at least two independent experiments using microtubular protein from different preparations.

### Computational studies

Docking studies were carried out as previously described^26^. The protein flexibility was taken into account by using 50 tubulin complexes with different ligands bound at the colchicine site retrieved from the pdb. The proteins were superimposed using the amino acids within 8 Å of any of the ligands and used in the docking experiments. Five additional structures came from a molecular dynamics run of a complex with an indolecombretastatin bound at the colchicine site^26^. Dockings were performed in parallel with PLANTS with default settings^27^ and 10 runs per ligand and with AutoDock 4.2^28^ runs applying the Lamarckian genetic algorithm (LGA) 100−300 times for a maximum of 2.5 × 10^6^ energy evaluations, 150 individuals, and 27,000 generations maximum. For each virtual ligand 550 poses were generated with PLANTS and AutoDock generated from 550 to more than 1500 poses. The colchicine subzones occupied by every pose were automatically assigned using in-house KNIME pipelines^29^. The programs’ docking scores were converted into Z-scores and the poses with best scores were visually compared until a consensus pose obtaining the best consensus scores for both programs could be selected and taken as the docking result. Docked poses were analysed with Chimera^30^, Marvin^31^, OpenEye^32^, and JADOPPT^33^.

## Results and discussion

### Chemistry

**Scheme 1.**
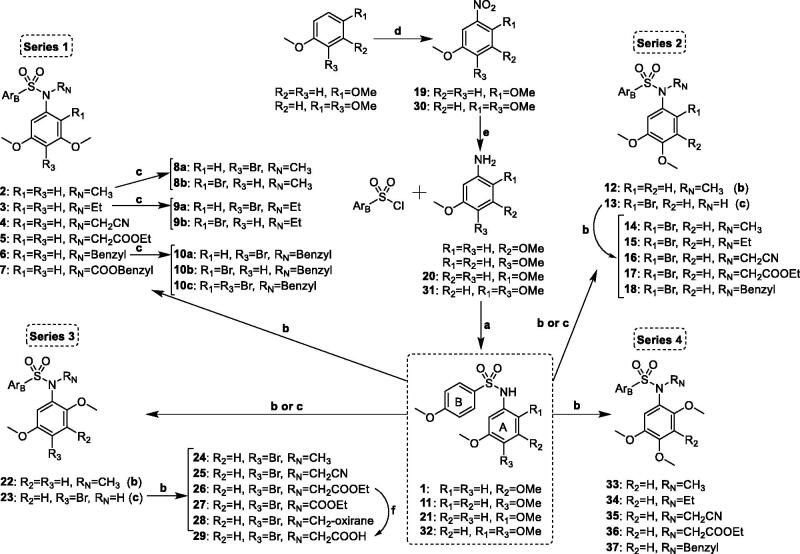
Synthesis of new tubulin inhibiting sulphonamides. Ar_B_: *p*-methoxyphenyl. Reagents, conditions, and yields: (a) Pyridine, CH_2_Cl_2_, rt, 6 h, 95–99%. (b) R_N_ = CH_3_: CH_3_I, KOH, CH_3_CN, rt, 24 h, 84–95%; R_N_ ≠ CH_3_: R_N_-halogen, K_2_CO_3_, dry DMF, rt, 24 h, 72–99%. (c) NBS, CH_2_Cl_2_, rt, 12 h, 62–95%. (d) HNO_3_, AcOH, 0 °C, 4 h, 85–94%. (e) H_2_, Pd(C), EtOAc, rt, 48 h, 99%. (f) KOH/MeOH, rt, 30 min, 87%.

The TMP ring is common to many colchicine site ligands and is considered important for high antiproliferative activity and tubulin binding. In an attempt to find new antimitotic sulphonamides with TMP replacements we have explored the effect of additional methoxy (methoxy scan) and/or bromo (bromo scan) substituents at different positions on the basic pharmacophore of the TMP moiety: a phenyl ring with a 5-methoxy aimed at recognising the thiol group of Cys241β of tubulin. Therefore, a new family of 4-methoxybenzenesulphonamides with methoxy and bromo substituents at different positions of the *N*-(5-methoxyphenyl) ring and with different substitutions on the sulphonamide nitrogen has been synthesised (Scheme 1 and Table 1). As a result of the methoxy scan, four main structural families have been sampled (Figure 1): 1) 3,5-dimethoxyphenyl (series 1); 2) 3,4-dimethoxyphenyl (series 2); 3) 2,5-dimethoxyphenyl (series 3); and 4) 2,4,5-trimethoxyphenyl (series 4). For the first three, substitutions of aromatic hydrogens by bromo atoms have been additionally carried out, with the fourth one being the methoxylated equivalent of the 4-bromo-2,5-dimethoxyphenyls of series 3. The 3,5- and 3,4-dimethoxyphenyl are the mono demethoxylated analogs of the TMP, and have been previously shown to result in severe potency reductions, with occasional exceptions such as in the 3,5-dimethoxyphenyl triazole analogs of combretastatin A-4^34^. The 2,5-dimethoxyphenyl substitution pattern has as well yielded modest results at best^15^^,^^35–38^, and only combined with bulky and hydrophobic B ring derivatives such as the carbazolesulphonamides^38^^,^^39^ or quinazolinone sulfamates^40^ achieved good antiproliferative results.

**Table 1.** Structures and biological evaluation of new synthetised sulphonamides.

				Comp	Antiproliferative activity IC_50_ (nM)^a^				TPI^b^	
R_1_	R_2_	R_3_	R_N_		HeLa	MCF7	HT-29	HT-29 Verap^c^	TPI % 10 µM	IC_50_ (µM)	
**Series 1**										
H	OCH_3_	H	H	**1**	>1000	>1000	>1000	n.d.	0	>10
H	OCH_3_	H	CH_3_	**2**	877	765	>1000	n.d.	10	>10
H	OCH_3_	H	Et	**3**	>1000	>1000	>1000	n.d.	0	>10
H	OCH_3_	H	CH_2_CN	**4**	>1000	>1000	>1000	n.d.	0	>10
H	OCH_3_	H	CH_2_COOEt	**5**	>1000	>1000	>1000	n.d.	0	>10
H	OCH_3_	H	Benzyl	**6**	>1000	>1000	>1000	n.d.	0	>10
H	OCH_3_	H	COOBenzyl	**7**	>1000	>1000	>1000	n.d.	0	>10
H	OCH_3_	Br	CH_3_	**8a**	497	99	495	293	10	>10
H	OCH_3_	Br	Et	**9a**	417	n.d.	610	n.d.	0	>10
H	OCH_3_	Br	Benzyl	**10a**	310	335	450	533	0	>10
Br	OCH_3_	H	CH_3_	**8b**	>1000	>1000	>1000	n.d.	0	>10
Br	OCH_3_	H	Et	**9b**	>1000	>1000	>1000	n.d.	0	>10
Br	OCH_3_	H	Benzyl	**10b**	>1000	>1000	>1000	n.d.	0	>10
Br	OCH_3_	Br	Benzyl	**10c**	>1000	>1000	>1000	n.d.	0	>10
**Series 2**										
H	H	OCH_3_	H	**11**	>1000	>1000	>1000	n.d.	4	>10
H	H	OCH_3_	CH_3_	**12**	>1000	>1000	>1000	n.d.	0	>10
Br	H	OCH_3_	H	**13**	510	n.d.	543	n.d.	0	>10
Br	H	OCH_3_	CH_3_	**14**	557	n.d.	907	n.d.	0	>10
Br	H	OCH_3_	Et	**15**	313	660	365	500	24	>10
Br	H	OCH_3_	CH_2_CN	**16**	407	575	360	750	16	>10
Br	H	OCH_3_	CH_2_COOEt	**17**	>1000	>1000	>1000	n.d.	0	>10
Br	H	OCH_3_	Benzyl	**18**	>1000	>1000	>1000	n.d.	38	>10
**Series 3**										
OCH_3_	H	H	H	**21**	227	350	187	187	0	>10
OCH_3_	H	H	CH_3_	**22**	177	153	250	305	4	>10
OCH_3_	H	Br	H	**23**	45	25	72	30	65	6.9
OCH_3_	H	Br	CH_3_	**24**	33	19	123	160	59	7.6
OCH_3_	H	Br	CH_2_CN	**25**	36	86	97	85	51	7.6
OCH_3_	H	Br	CH_2_COOEt	**26**	577	700	2100	867	28	>10
OCH_3_	H	Br	COOEt	**27**	377	220	525	170	44	>10
OCH_3_	H	Br	CH_2_-oxirane	**28**	495	n.d.	860	n.d.	32	>10
OCH_3_	H	Br	CH_2_COOH	**29**	>1000	>1000	>1000	n.d.	8	>10
**Series 4**										
OCH_3_	H	OCH_3_	H	**32**	410	580	360	553	3	>10
OCH_3_	H	OCH_3_	CH_3_	**33**	413	99	500	577	0	>10
OCH_3_	H	OCH_3_	Et	**34**	313	715	500	1070	19	>10
OCH_3_	H	OCH_3_	CH_2_CN	**35**	500	67	365	415	5	>10
OCH_3_	H	OCH_3_	CH_2_COOEt	**36**	>1000	>1000	>1000	n.d.	0	>10
OCH_3_	H	OCH_3_	Benzyl	**37**	833	n.d.	1250	n.d.	7	>10
H	OCH_3_	OCH_3_	H	**TMP**^41^	240	375	897	975	0	>20
H	OCH_3_	OCH_3_	CH_3_	**TMP**^41^	71	127	143	117	35	>20
**Combretastatin A-4**					2	1	305	327	100	3
**ABT-751**					388	180	213	250	69	4.4

Compounds have been grouped in four different series regarding the position of the methoxy groups on the aromatic A ring.

^a^Drug concentration required to inhibit the growth of selected human tumour cell lines by 50%, relative to untreated controls after 72 h of drug exposure.

^b^*In vitro* tubulin polymerisation inhibition.

^c^IC_50_ in human colon adenocarcinoma HT-29 cell line in the presence of the Pgp/MDR1 inhibitor verapamil (10 µM).

^d^n.d.: not determined.

Thirty seven new benzenesulphonamides (series 1–4) were synthesised following the synthetic approach outlined in Scheme 1. The basic diphenylsulphonamide skeletons of the four series (**1**, **11**, **21,** and **32**) were built up in good yields (95–99%) by the reaction between the corresponding anilines and 4-methoxybenzenesulphonyl chloride. For series 3 and 4, anilines **20** and **31** were previously prepared by nitration-reduction reaction sequences. Substituents of different length, polarity, and size (e.g. small alkyl chains such as methyl or ethyl, more polar acetonitrile or acyl derivatives, or larger benzyl groups) were appended on the sulphonamide bridge through alkylation reactions carried out under basic conditions that favour the formation of the nucleophilic sulphonamide anion. Methylations with methyl iodide and KOH in dry acetonitrile were achieved in good yields (84–95%) to give **2**, **12**, **14**, **22**, **24**, and **33**. Other substitutions were performed with the corresponding alkyl halides and K_2_CO_3_ as a base in dry DMF (**3**–**7**, **15**–**18**, **25**–**28**, and **34**–**37**). Brominations (**8a**–**10c**, **13**, and **23**) were carried out using NBS as a source of electrophilic bromine. The positions of the bromine atom(s) were established by the proton couplings in the ^1^H NMR spectra. The synthesised compounds were characterised by ^1^H and ^13^C NMR, IR, HRMS, and melting points (if crystals), and agree with the proposed molecular structures.

The highly hydrophobic nature of the colchicine domain drives the physicochemical properties of the colchicine-site ligands towards non-drug-like physicochemical properties and makes the aqueous insolubility one of the recurrent problems of colchicine site ligands. The aqueous solubilities (Table 2) of representative compounds in pH 7 phosphate buffer were spectrophotometrically measured, showing improvements with respect to the marginal solubility of CA-4 (1 µg/mL). The best results were obtained for series 4, with values 100–200 times higher than that obtained for CA-4. Moreover, 6 out of 16 tested compounds were up to five times more soluble than the orally administered tubulin-inhibiting sulphonamide ABT-751 (40 µg/mL). The introduction of substituents at the sulphonamide and/or of bromo atoms led to a solubility decrease compared to the unsubstituted pairs.

**Table 2.** Aqueous solubility in pH 7.0 phosphate buffer of some representative compounds. Solubility values are expressed in µg/mL.

Comp	Solub (µg/mL)	Comp	Solub (µg/mL)	Comp	Solub (µg/mL)
**2**	35	**16**	13	**32**	213
**11**	82	**21**	13	**33**	157
**12**	70	**22**	25	**34**	122
**13**	22	**23**	9	**35**	15
**14**	61	**24**	1	**CA-4**	1
**15**	26	**25**	1	**ABT-751**	40

#### Diphenylsulphonamides are potent in vitro antiproliferative agents: analysis of the structural-modification scans and structure-activity relationships

We screened by the XTT method all the synthesised sulphonamides in three independent assays at 1 µM for antiproliferative effect 72 h after treatment against the human tumour cell lines HeLa (cervix epithelioid carcinoma), MCF7 (breast adenocarcinoma), and HT-29 (colon adenocarcinoma). We classified benzenesulphonamides inhibiting cell proliferation by more than 40% in the screening phase as actives and the rest as inactives. More than half (21 out of 37) of the benzenesulphonamides were actives, with more actives in series 3 (8 out of 9) and 4 (5 out of 6) than in series 1 (4 out of 14) and 2 (4 out of 8). Methoxybenzenesulphonamides of trisubstituted anilines represented most of the actives (18 out of 21), thus indicating that a larger size is important to achieve sub-micromolar potency. We further assayed the actives at concentrations between 10 µM and 0.1 nM and IC_50_ (half-maximal inhibitory concentration) values were calculated (Table 1) along with those of CA-4, ABT-751, and *N*-(3,4,5-trimethoxyphenyl)-4-methoxyphenylsulphonamide^41^, used as references. The distributions of the number of actives and relative potencies within the series were similar, thus indicating that the substitution pattern is an important factor for the antiproliferative potency. Interestingly, the series with the most similar substitution patterns to the 3,4,5-trimethoxyphenyl ring (series 1, 2, and 4) are not the ones with more potent representatives (IC_50_ values lower than 100 nM), which correspond to the 4-bromo-2,5-dimethoxyphenyl derivatives (**23**–**25**) clustered in series 3. The most sensitive cell line was the human breast cancer cell line MCF7 (6 compounds with IC_50_ values lower than 100 nM), followed by HeLa (3 compounds with IC_50_ values lower than 100 nM), and HT-29 (2 compounds with IC_50_ values lower than 100 nM) was the least sensitive. The lower sensitivity of HT-29 is not unexpected, as it is often observed for tubulin inhibitors and has been associated with efflux by MDR proteins^42^, autophagy induction^21^, and/or metabolic glucuronidation reactions^43^. However, MCF7 is not usually more sensitive than HeLa as observed here, in particular for compounds **8a**, **33**, and **35** which are 4–7 fold more potent against MCF7 than against HeLa.

For simplicity, in the following, we will use the IC_50_ values of the *N*-methylated sulphonamides as representative for the pairwise comparison of the different substitution patterns on the aniline rings (series 1–4). Similar effects, usually with lower potencies, are obtained for other substituents on the sulphonamide nitrogen. Regarding the methoxy scan, the more favourable substitution pattern for the dimethoxylated anilines is the 2,5- (series 3: **22**) with potencies just slightly lower than those of the TMP analogues^41^, while the structurally more similar to the TMP (just differing in the removal of one of the methoxy groups) 3,5- (series 1: **2**), and 3,4- (series 2: **12**) are inactive or active in the micromolar range. This result is different from similar modifications with other bridges between the aromatic rings, for instance with triazoles where the 3,5-dimethoxyphenyl ring yielded equally potent analogs as the TMP^34^.

The methoxy or bromo scans of additional substituents on the disubstituted anilines resulted mostly in potency improvements, thus confirming that larger sizes are preferred. This favourable effect on the antiproliferative potency varies with the initial dimethoxy substitution pattern and the position and nature of the substituents. For series 1, the introduction of an additional bromo substituent on the 3,5-dimethoxy is slightly favourable at position 4 (e.g. compare **2** with **8a**) where it gives a much TMP-like 3,4,5- substitution pattern, whereas it is detrimental at position 2 (e.g. compare **2** with **8 b**) where it results in a 2,3,5- substitution pattern. Similarly, for series 2 an additional bromo or methoxy group (series 4) at position 2 of the 4,5-dimethoxy (2,4,5-substitution pattern) results in modest potency improvements (compare **12** vs. **14** for bromo, or **12** vs. **33** for methoxy). Remarkably, an additional bromo at position 4 is highly favourable for the 2,5-dimethoxy series 3 (compare **22** vs. **24**) whereas a methoxy group, usually considered an important pharmacophoric element of the TMP ring for the interaction with tubulin^44^^,^^45^, is detrimental at this position except for MCF7 (compare **22** vs. **33**), both again for the 2,4,5-substitution pattern. The structure-activity relationships (SAR) results show an interesting pattern of especially high antiproliferative potency against the human breast cancer cell line MCF7 for the series with 2,4,5-trisubstituted anilines, even compared to the usually highly sensitive HeLa cells^41^. A comparison of the compounds with 2,4,5-trisubstituted anilines shows that a 2,4,5-trimethoxyphenyl ring (**33**) is worse than the 3,4,5-trimethoxyphenyl ring, except for MCF7, against which they are of similar potency. Replacement of one of the methoxy groups with a bromine atom is slightly detrimental at position 2 (e.g. **14**) but highly favourable at position 4 (e.g. **24**), where it results in the most potent compounds, even compared to their TMP pairs.

Finally, for the bromo scan, the introduction of the additional bromo substituent at position 4 results in high potency improvements (e.g. compare **2** vs. **8a** for series 1, or **22** vs. **24** for series 3), whereas at position 2 it is neutral or even detrimental (e.g. compare **2** vs. **8b** for series 1, **12** vs. **14** for series 2, or **33** vs. **14** for series 4). The introduction on the basic pharmacophore of a substituent at position 2 (either a methoxy or a bromo) is favourable, whereas at position 3 (a methoxy) it is detrimental or neutral at best. Finally, a substituent at position 4 is also favourable, more for bromo than for methoxy groups. The structural combination of these favourable modifications is also positive, thus resulting in the highest antiproliferative potencies, even better than those of the well-established TMP.

Concerning the substituent on the sulphonamide nitrogen, hydrogen or small groups (methyl, ethyl, or acetonitrile) resulted in optimal potency for every series (**2**, **8a**, **9a**, **13**–**16**, **21**–**25,** and **32**–**35**), whereas larger substituents such as acetates (**5**, **17**, **26**, **29**, and **36**), carboxylates (**7** and **27**), or benzyls (**10b**–**c**, **18**, and **37**) showed reduced potencies or rendered the compounds inactive, except for the benzyl group of the 3,4,5- trisubstituted compound **10a** of the series 1. These results are different from previously described sulphonamides, including those with TMP phenyl rings, where methyl groups are usually strongly preferred^3^^,^^46^^,^^47^. Acetonitrile substitutions on the sulphonamide nitrogen (**16**, **25**, and **35**) showed similar potencies (IC_50_ HeLa = 407, 36, and 500 nM, respectively) with respect to the less polar methylated analogs (**14**, **24**, and **33**) (IC_50_ HeLa = 557, 33, and 413 nM, respectively).

In summary, best outcomes were achieved by the 2,4,5-trisubstitution pattern instead of the 3,4,5- of the TMP ring, with optimal results for a 2,5-dimethoxy phenyl ring together with a 4-bromo atom (**23**–**25**). Compound **25**, which combines these structural characteristics with the novel acetonitrile moiety on the sulphonamide bridge was therefore selected for further characterisation of the mechanism of action in HeLa, MCF7, and HT-29 human tumour cell lines.

#### Benzenesulphonamides are *in vitro* antiproliferative agents against the CA-4 resistant cell line HT-29 and do not suffer from MDR efflux or UDPGluc-transferase conjugation reactions

The human colon adenocarcinoma cell line HT-29 shows low sensitivity towards CA-4 and other colchicine site ligands. This low sensitivity has been attributed to drug efflux by MDR expression^42^, autophagy induction^21^, and drug metabolism by UDP glucuronyltransferase enzymes^12^. All the synthesised sulphonamides were evaluated against this cell line (Table 1), both in the absence and the presence of the non-selective Pgp/MDR1 inhibitor verapamil at a concentration of 10 µM, which was shown to not affect HT-29 cell proliferation^20^^,^^48^. A comparison of the pairs of IC_50_ values provides a measure of the sensitivity of the drugs to efflux by MDR pumps, as previously described^49^^,^^50^. Potency differences higher than 3-fold were considered indicative of sensitivity to MDR efflux. Compound **27** was the only active benzenesulphonamide with a difference in IC_50_ values larger than 3-fold arising from the co-treatment with the MDR inhibitor. These results suggest that the benzenesulphonamides are not substrates of the MDR efflux, which is one of the main ways tumour cells use to develop resistance against tubulin inhibitors^8^.

Most of the active compounds showed similar potencies against HeLa and HT-29, with IC_50_ values encompassed within a 3 to 4-fold range. These differences are much lower than that observed for CA-4 (>100 fold) and result in a potency improvement of the new sulphonamides of series 3 (i.e. **21**–**25**) against HT-29 compared with CA-4. The different sensitivity to CA-4 compared to the benzenesulphonamides seems not to be due to drug efflux, as co-treatment with verapamil did not result in a potency increase for CA-4. Alternatively, the lack of the 3 hydroxy group that is the point of metabolism by UDP-glucuronyltransferases in CA-4 might explain the observed difference. Sulphonamides are potential substrates of UDP-glucuronyltransferases, but we have found no differences between unsubstituted and substituted sulphonamides against HT-29 (e.g. compare **23** with **24** and **25**), thus suggesting that they do not experience the inactivating conjugation reaction and providing an explanation for the higher effect against this cell line.

#### *In vitro* cell growth inhibition against the human non-tumorigenic cell line HEK293

The antiproliferative IC_50_ values of sulphonamides **22**–**25** against the embryonic kidney human cell line HEK293 were used as a surrogate for toxicity against non-tumorigenic cells (Table 3). HeLa and MCF7 tumour cells were more sensitive than the non-tumorigenic HEK293 cells towards sulphonamides **22** (IC_50_ values of 177 and 153 nM compared with 517 nM) and **24** (IC_50_ values of 33 and 19 nM, compared with 91 nM), thus showing promising selectivity indexes up to 3- to 5-fold. The tumour cell lines were similarly sensitive to sulphonamides **23** (IC_50_ values of 45 and 25 nM, compared with 43 nM for the non-tumorigenic cells) and **25** (IC_50_ values of 36 and 86 nM, compared with 45 nM), with selectivity indexes from 0.5 to 2-fold. These results suggest that the new diarylsulphonamides have a modest safety profile for potential application in the treatment of tumour cells.

**Table 3.** Cell growth inhibition of the most potent compounds against the non-tumorigenic human embryonic kidney cell line HEK293.

Antiproliferative activity IC_50_ (nM)	
Comp	HEK293
**22**	517
**23**	43
**24**	91
**25**	45

#### Diarylsulphonamides of series 3 are tubulin polymerization inhibitors (TPI) *in vitro*

To confirm that the antiproliferative effect of the compounds is due to inhibition of tubulin, all the synthesised sulphonamides were screened at a concentration of 10 µM for inhibition of the thermally induced assembly of microtubular protein isolated from calf brain. Only compounds from series 3 (**23**–**28**) showed consistently inhibition of tubulin polymerisation, with the rest of the series showing low inhibition percentages or no inhibition at all, even for compounds with sub-micromolar antiproliferative potencies (e.g. **8a**–**10a**, **13**–**16**, or **33**–**37**). For those compounds of series 3 that reduced polymerisation by more than 50% at the tested concentration, we determined the IC_50_ values of tubulin polymerisation inhibition (Table 1 and Supplemental Figure 1). Only the most potent antiproliferative compounds of series 3 (**23**–**25**) showed IC_50_ values below 10 µM (IC_50_ = 6.9, 7.6, and 7.6 µM, respectively). These compounds are also the most potent ones in proliferation assays, thus suggesting TPI as the potential mechanism of action. As previously reported for many anti-tubulin agents^15^, no correlation is often observed between the IC_50_ values of TPI and the cell growth inhibition values, and compounds with different scaffolds show very different TPI to antiproliferative ratios. This discrepancy is probably due to the accepted proposal that the antiproliferative effects observed at low treatment doses are caused by the interference with microtubule dynamics but not by the alteration of polymer mass, which only occurs at high drug concentrations^1^.

#### Diarylsulphonamide 25 interferes with the microtubule network in cells and causes mitotic arrest followed by apoptotic cell death

**Figure 2.**
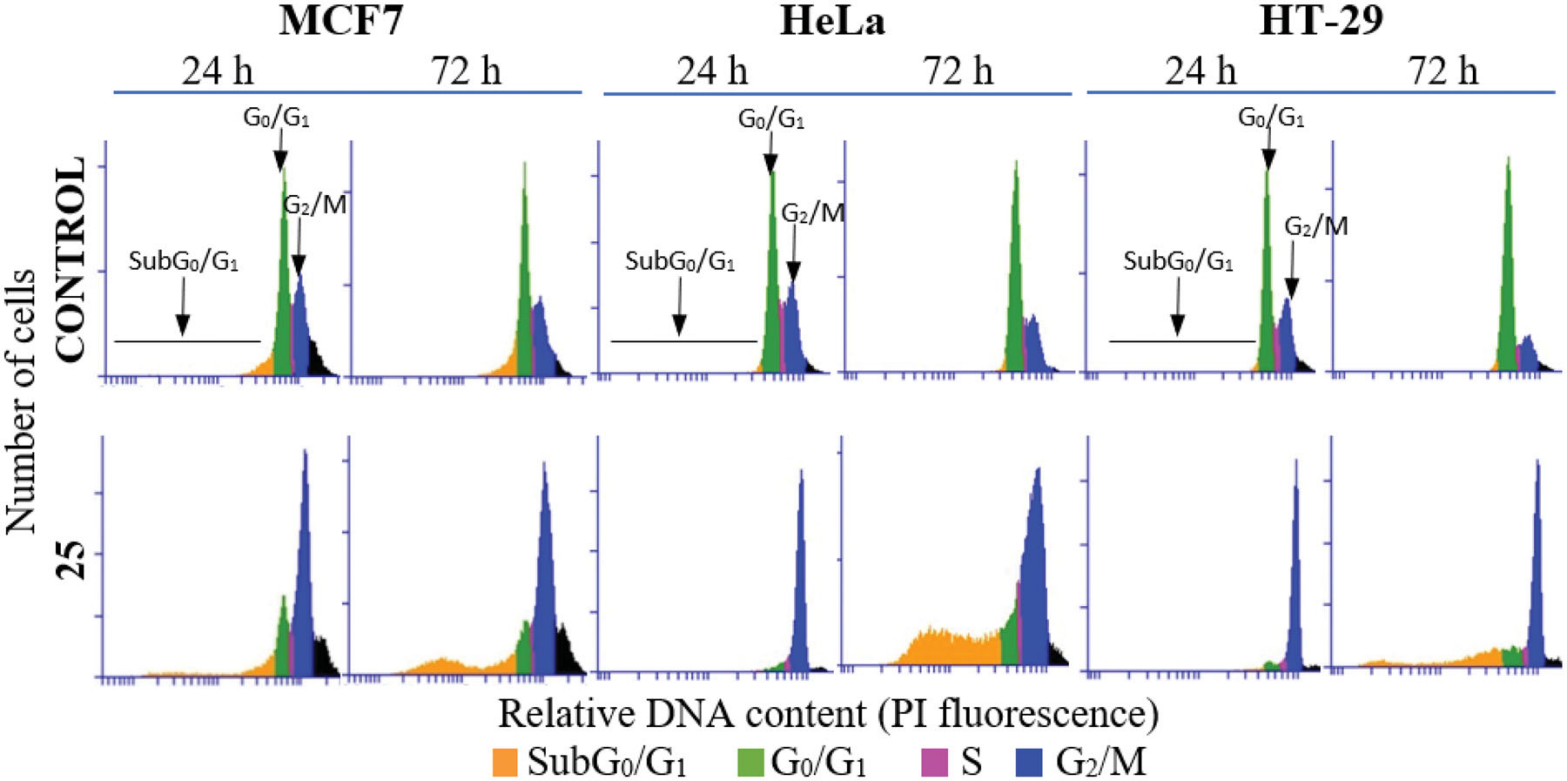
DNA content frequency histograms representing the progression on the cell cycle profile from 24 to 72 h in MCF7, HeLa, and HT-29 cell lines untreated (control) or treated with compound **25** at 175 nM. The position of subG_0_/G_1_, G_0_/G_1_, and G_2_/M peaks are indicated by arrows. Most of the cells are arrested at G_2_/M peak after 24 h treatment. Cell death is observed 72 h post-treatment by the increase in the percentage of cells at subG_0_/G_1_ region. The histograms shown are representative of three independent experiments. Control cells were run in parallel.

**Figure 3.**
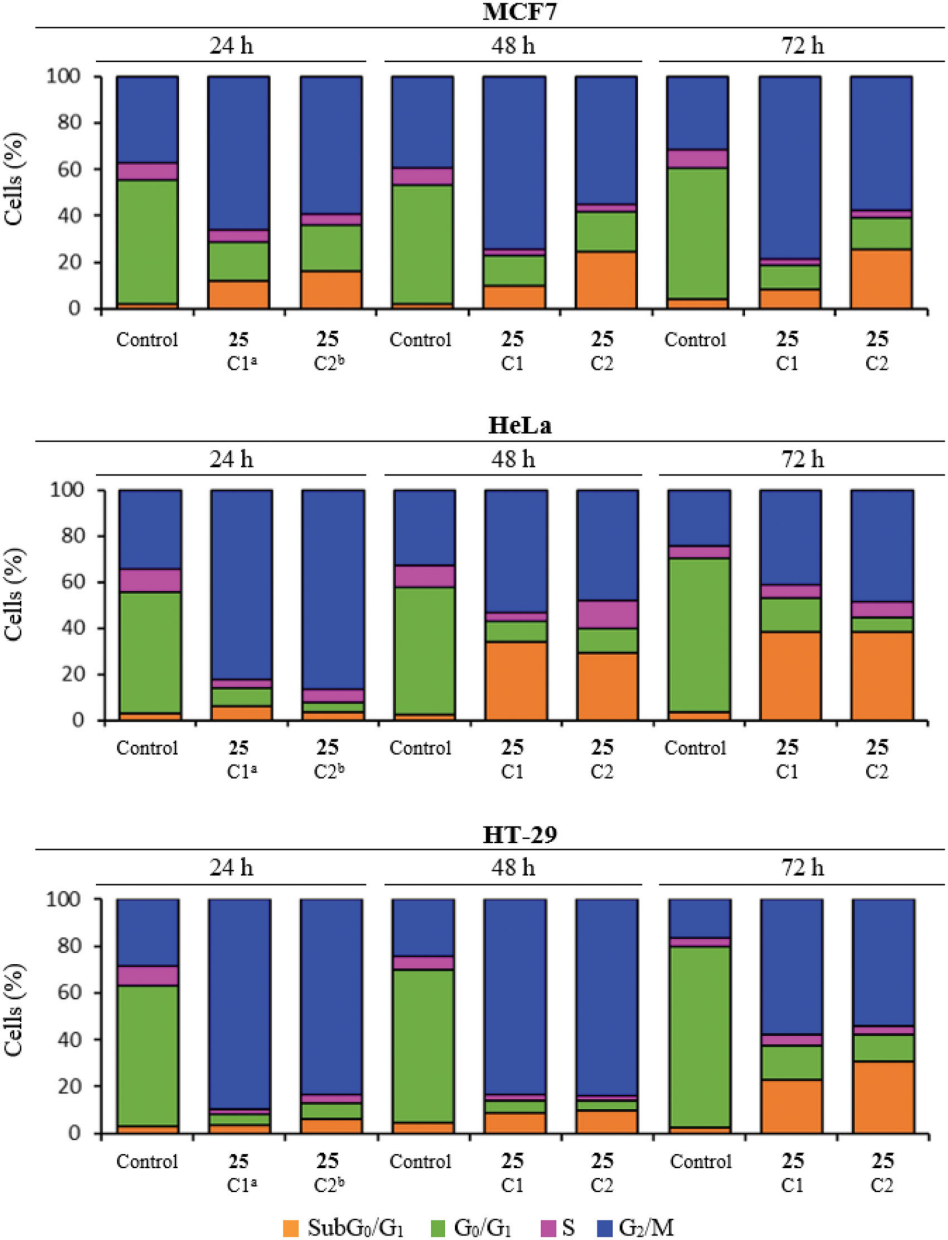
Time-course effect of compound **25** on cell cycle distribution in MCF7, HeLa, and HT-29 cell lines. ^a^C1: 87.5 nM. ^b^C2: 175 nM. Cells were incubated in the absence (control) or the presence of compound **25** at 87.5 or 175 nM for 24, 48, and 72 h, stained with PI, and their DNA content was analysed by fluorescence flow cytometry. The proportion of cells in each phase of the cell cycle was quantified and expressed in percentages. The data shown are the average of three independent experiments. Control cells were run in parallel.

The time-course effects of compound **25** (87.5 and 175 nM) on the cell cycle populations of MCF7, HeLa, and HT-29 cells were studied to further interrogate its mechanism of action. Cells were stained with Propidium Iodide (PI) and the DNA content measured by fluorescence flow cytometry was used to evaluate the distribution of cells along the cell cycle at 24 h time steps from 24 to 72 h post-treatment (Figures 2 and 3).

Compound **25** at both concentrations measured (87.5 nM and 175 nM) arrested almost completely cervix cancer HeLa cells (82% and 86%, respectively) and colon adenocarcinoma HT-29 cells (90% and 83%) at the G_2_/M phase 24 h after treatment. The mitotic arrest was sustained at the 48 h time point for HT-29 (84%) cells along with a small increase of the subG_0_/G_1_ population (9%), and later followed at 72 h by a further increase of the subG_0_/G_1_ population (23% and 31%) at the expense of the G_2_/M phase (58% and 54%). For the HeLa cells, this trend was reproduced but in a shorter timescale, with the subG_0_/G_1_ population (34% and 29%) increasing significantly at 48 h at the expense of the G_2_/M phase (54% and 48%), showing a small additional advance in this direction at the 72 h timepoint (subG_0_/G_1_ population: 38%; G_2_/M phase 41% and 49%). Breast cancer MCF7 cells showed a more modest G_2_/M arrest at 24 h for both drug concentrations (66% and 60%), accompanied by an early increase in the subG_0_/G_1_ (apoptotic) populations (12% and 16%). After the initial 24 h timepoint, differences for the two drug concentrations were apparent. At the lower dose, the distribution of cells remained mostly unchanged for 72 h, whereas for the higher dose an increment in the subG_0_/G_1_ population (24%) at the expense of the G_2_/M phase (55%) was observed at 48 h and maintained at 72 h (subG_0_/G_1_ population: 26%; G_2_/M phase 58%), in a similar way as that observed for HeLa cells but with a lower advance of the subG_0_/G_1_ population.

Therefore, the lead compound **25** caused a mitotic arrest of the cell cycle at the G_2_/M phase and an increase in the subG_0_/G_1_ population that varied depending on the cell line in the time of onset and advance, and in the concentration dependency. These results agree with the proposed mechanism of action affecting the mitotic machinery by interfering with tubulin polymerization^1^.

To further ascertain the apoptotic cell death suggested by the cell cycle studies, MCF7, HeLa, and HT-29 cells were double-stained with Annexin V-FITC (AnV) and propidium iodide (PI) and then analysed by flow cytometry 72 h after treatment with compound **25** at 87.5 and 175 nM. Cells were considered alive as being double negative (AnV–/PI–), in Early Apoptosis (EA) as being AnV+/PI–, in Late Apoptosis (LA) as being double-positive (AnV+/PI+) and in necrosis as being AnV–/PI+ (Table 4). Live control cells were always >90%. Consistently with the cell cycle results, an apoptotic response was observed for the HT-29 cell line (23% to 36%), and for HeLa cells, which had an almost complete apoptotic profile (>90%). A concentration-dependent apoptotic response similar to that observed in the cell cycle experiments was observed for treated MCF7 cells, with 18% apoptotic cells at the lowest concentration, which increased to 33% with the concentration shift. The weak build-up of the apoptotic response in MCF7 cells may putatively depend on its deficiency of caspase 3^51^^,^^52^, the hub effector of the apoptotic machinery. These results are in line with the cell cycle experiments and suggest that the lead compound **25** exhibits its antitumor effect by causing a mitotic arrest that triggers an apoptotic response, as has been previously described for other antimitotic agents^53^.

**Table 4.** Cell death quantification.

	MCF7	HeLa	HT-29
CONTROL			
Live^a^	91.1	96.8	92.3
Early apoptosis^b^	0.9	0.6	3.3
Late apoptosis^c^	5.9	1.9	4.1
Necrosis^d^	2.1	0.6	0.2
**25**			
87.5 nM			
Live	82.1	6.5	76.2
Early apoptosis	11.7	52.3	16.4
Late apoptosis	5.8	39.7	6.8
Necrosis	0.5	1.5	0.7
175 nM			
Live	65.7	4.1	61.7
Early apoptosis	23.8	47.5	20.4
Late apoptosis	9.5	45.3	15.3
Necrosis	1.0	3.1	2.5

MCF7, HeLa, and HT-29 cell lines were incubated in the absence (control) or the presence of compound **25** at 87.5 or 175 nM for 72 h, double-stained with Annexin V-FITC (AnV) and propidium iodide (PI) and analysed by flow cytometry. Data shown represent the average of three independent experiments and are expressed in percentages. Control cells were run in parallel.

^a^Cells were considered live as being double-negative (AnV–/PI–).

^b^In early apoptosis as being AnV-positive.

^c^In late apoptosis as being double-positive (AnV+/PI+).

^d^In necrosis as being PI-positive.

**Figure 4.**
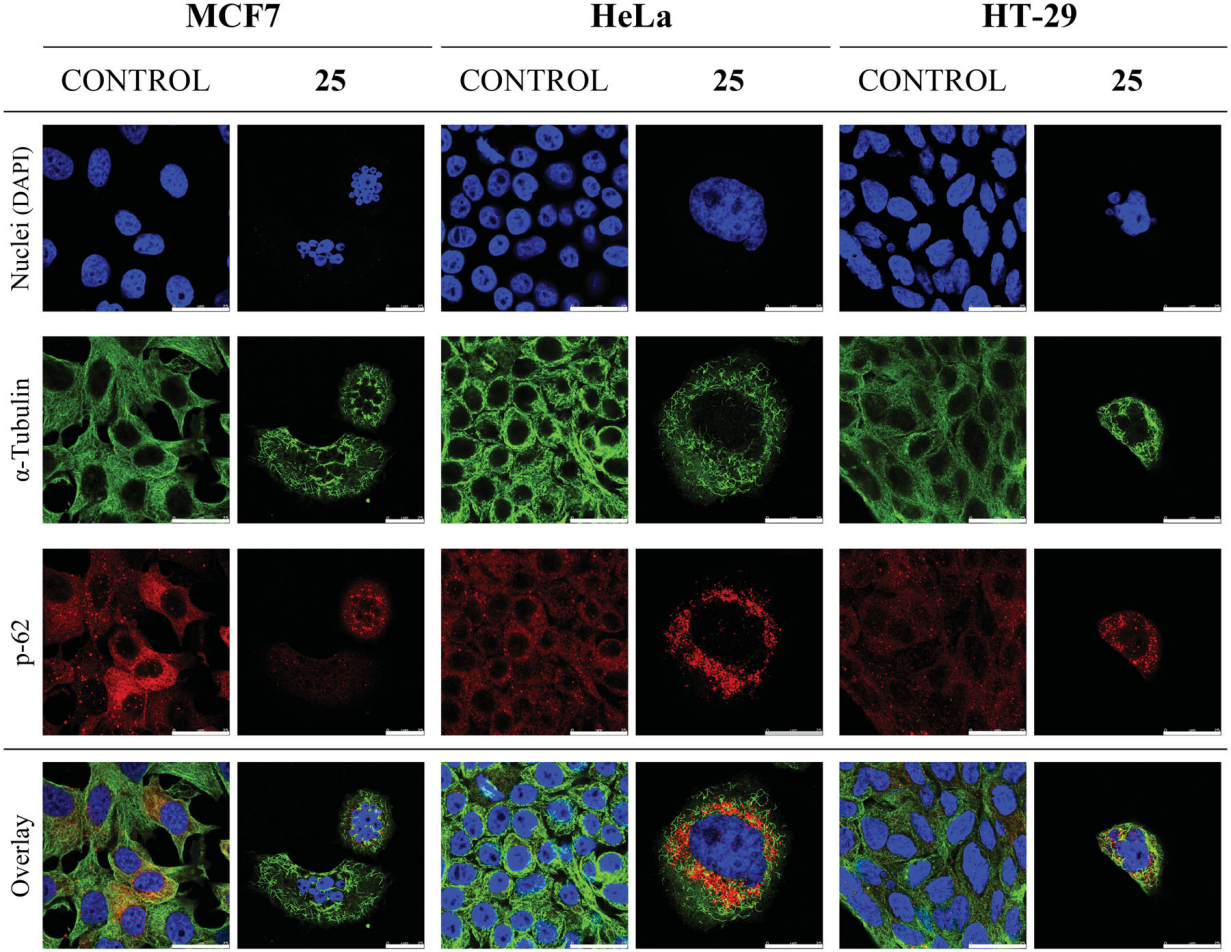
Effect of compound **25** (175 nM) on the microtubule system and autophagy of MCF7, HeLa, and HT-29 cells after 72 h of treatment. Microtubules were stained with anti-α-tubulin antibody (green fluorescence), p-62 protein with anti-SQSTM1/p62 antibody (red fluorescence), and cell nuclei with DAPI (blue fluorescence). Preparations were analysed by confocal microscopy. Control cells were run in parallel. Scale bar: 25 µm. The photomicrographs shown are representative of three independent experiments.

To confirm the effect on the microtubular system of treated cells, the microtubule network of MCF7, HeLa, and HT-29 cell lines was studied by immunofluorescent microscopy. Microtubules (green fluorescence) were stained with anti-α-tubulin antibody after 72-h exposure to compound **25** at 175 nM, the p-62 autophagosome marker (red fluorescence) was stained with anti-SQSTM1/p62 antibody and cell nuclei (blue fluorescence) were stained with DAPI (Figure 4). Compound **25** disrupted the microtubule network of MCF7, HeLa, and HT-29 cell lines. Besides, as expected for tubulin-binding drugs^54^^,^^55^, alterations in chromatin organisation could be observed. Condensation and fragmentation of chromatin in multilobulated nuclei resembling bunches of grapes are clear in treated MCF7 and HT-29 cells; whereas giant nuclei are present in HeLa cells. These results further support that the lead compound **25** exhibits its antitumor effect by interfering with the microtubule network.

#### Diarylsulphonamide 25 causes a downregulation of the autophagosome marker p-62

**Figure 5.**
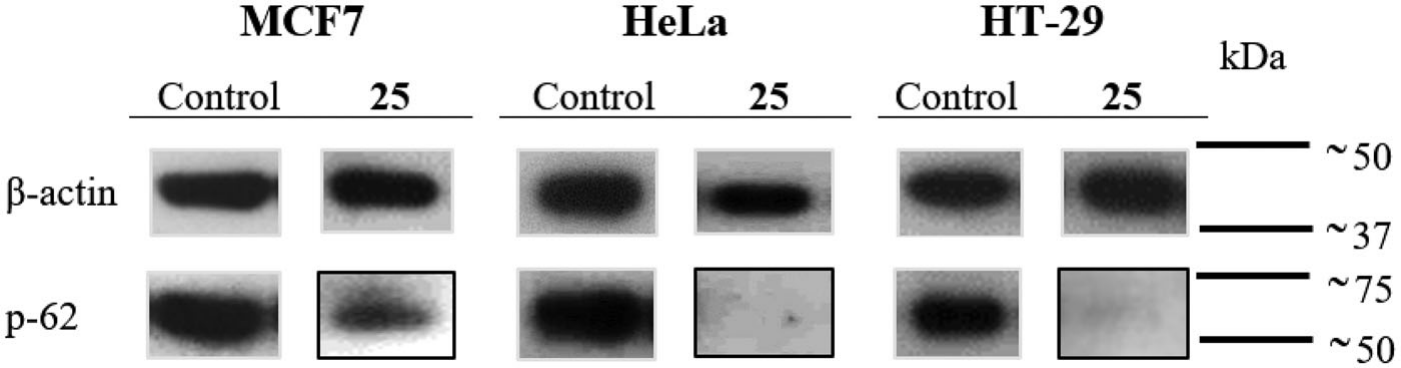
Western blot analysis of autophagosome marker p-62, on MCF7, HeLa and HT-29 cell extracts after 24 h of treatment with compound **25** at 175 nM. β-actin constitutive protein was used as an internal control. Control cells were run in parallel.

The confocal microscopy studies showed downregulation and perinuclear clumps of autophagosome marker p-62 upon drug treatment, in accordance with activated autophagy^56^ (Figure 4). Additional evidence of this mechanism was pursued by western blot analysis of the autophagosome marker p-62 after treatment with compound **25** at 175 nM (Figure 5). Treatment with compound **25** caused decay in p-62 levels in the MCF7 cell line and an almost complete reduction in the expression of p-62 in HeLa and the HT-29 cell lines after 24 h treatment. These results reveal activation of the autophagic process, which is associated with decreased p-62 levels^57^, and sustain that compound **25** exerts its action by interfering with microtubule dynamics and by activation of autophagy.

#### Molecular docking studies

**Figure 6.**
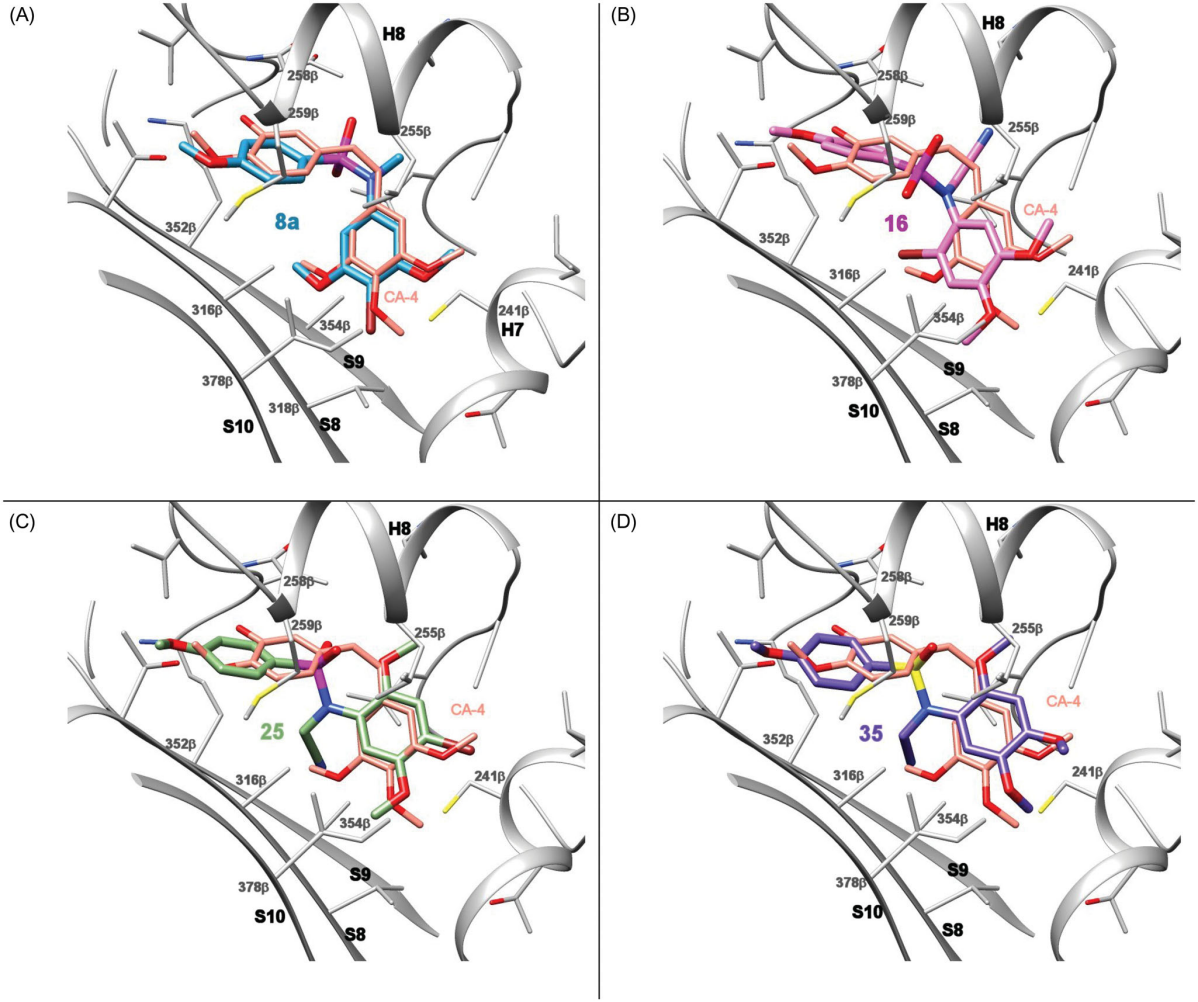
Consensus docking poses for representative compounds of each series: (A) **8a** (carbons in dodger blue) of series 1, (B) **16** (carbons in magenta) of series 2, (C) **25** (carbons in yellow-green) of series 3, (D) **35** (carbons in purple) of series 4. The secondary elements of the colchicine site are shown in a silver cartoon representation and labelled. In every panel, the X-ray structure of combretastatin A-4 (CA-4) in thinner sticks with carbons in orange is shown superimposed for comparison. Amino acids within 5 A of the ligands are shown as thin sticks.

The binding mode of the sulphonamides at the colchicine site has been studied using flexible docking studies. The protein conformational space has been sampled employing ensemble docking^58^ into 55 tubulin structures with different colchicine domain configurations induced by different ligands. Two docking programs with different scoring functions have been combined for the docking and scoring processes. For each ligand, the common pose shared by the two docking programs and with the highest (corresponding to lowest scoring energies) combined normalised scores for the two scoring functions was selected as the ligand-binding mode (Supplemental Table 1). All the ligands bound to the colchicine domain with the two phenyl rings (*p*-methoxyphenyl and *N*-phenyl) arranged similarly as rings A (trimethoxyphenyl) and B (3-hydroxy-4-methoxyphenyl) of combretastatin A-4 (Figure 6). Furthermore, with the only exception of compound **12** that binds in the reverse sense, in all cases the 4-methoxyphenyl rings bound at the B-ring site (zone 1) of combretastatin A-4 whereas the *N*-phenyl rings did so at the A-ring site (zone 2), thus reflecting their respective size and shape similarity that translates into the protein cavities that they lodge in. Regarding the protein sites preferred, most of the selected poses bind to sites similar in shape and size to combretastatin A-4 (the most frequent sites being those binding the β-lactam analogs of combretastatins, which likely reflects the larger space needed to allocate the sulphonamide bridge compared to the olefin of CA-4 itself). Concerning the conformation of the selected poses (Figure 6 and Supplemental Table 1), a different outcome was observed depending on the positions of the substituents of the phenyl ring occupying zone 2 and the bulkiness of the substituent on the sulphonamide nitrogen. For compounds with a 3,4,5-substituted *N*-phenyl ring such as those of series 1 (e.g. **8a**) a *cisoid* conformation of the sulphonamide that results in a very close overlap with CA-4 are observed (Figure 6(A)) unless polar or/and bulky substituents on the sulphonamide nitrogen cause the sulphonamide to adopt a *transoid* confirmation, which is the preferred one in most compounds having 2,3,5- (series 1) or 2,4,5-substituted (series 2-4) *N*-phenyl rings (Figure 6(B–D)). For all the compounds with a substituent *ortho* to the anilide nitrogen, the steric demand increases, therefore favouring a *transoid* disposition (Figure 6(B–D)). In every case, the disposition of the ring aims at fulfilling the pocket for the A-ring, and depending on the substituent *ortho* to the nitrogen atom of the *N*-phenyl ring differences in their binding mode are observed: for the 2-bromo- series 2 (Figure 6(B)) the *N*-substituent projects towards the interfacial surface between the α and β tubulin subunits, whereas for the 2-methoxy- series 3 (4-bromo-5-methoxy, Figure 6(C)) and 4 (4,5-dimethoxy, Figure 6(D)), except in this later case for the combination with the smaller substituents on the sulphonamide nitrogen (hydrogen or methyl), the *N*-substituent points towards S8–10, thus replacing one of the methoxy groups. This new binding mode explains the high potency observed for the acetonitrile substituent on the sulphonamide nitrogen, which was not observed for *N*-(3,4,5-trimethoxyphenyl)-4-methoxybenzenesulphonamides^41^.

## Conclusion

A focussed library of 37 new sulphonamides that represents a scan of the substitution of hydrogen atoms on the anilide ring of the basic colchicine-binding site pharmacophore *N*-(5-methoxyphenyl)-4-methoxybenzenesulphonamides with methoxy and/or bromo groups, grouped in 4 structural series, has been successfully prepared. The assessment of the antiproliferative activity of the library members against several human cancer cell lines showed that many compounds outperform analogs carrying the conventional TMP ring. Furthermore, the compounds are not substrates of the MDR-1 protein, as co-treatments with MDR-1 inhibitor verapamil do not enhance the antiproliferative potency against the HT-29 cell line. The structure-activity relationships of the series differ from the TMP analogs, with polar substituents better tolerated on the sulphonamide bridge, and a special susceptibility is observed for the breast cancer cell line MCF7, not paralleled by the TMP analogs. Unexpectedly, the most promising series is the more structurally dissimilar to the TMP, the 4-bromo-2,5-dimethoxyphenyl series 3. The effect of the most potent compounds on the microtubule system was confirmed by *in vitro* tubulin polymerisation inhibition. Compound **25** inhibits microtubule polymerisation *in cells* as shown by confocal microscopy studies and causes a mitotic arrest followed by a dose-dependent induction of apoptosis at later times, accompanied by autophagy induction. Docking experiments agree with binding to the tubulin colchicine-site in a different disposition that accounts for the high potency. In conclusion, the structural modifications provide access to new colchicine-site compounds, and lead compound **25** is a promising new candidate tubulin-binding antitumor drug.

## Supplementary Material

Supplemental Material
