# Supplementary material for: Methoxy and bromo scans on N-(5-methoxyphenyl) methoxybenzenesulphonamides reveal potent cytotoxic compounds, especially against the human breast adenocarcinoma MCF7 cell line
Source: J Enzyme Inhib Med Chem. 2021 Jun 9;36(1):1029–47. doi: 10.1080/14756366.2021.1925265 (PMC8205030; doi:10.1080/14756366.2021.1925265)
Supplement: Supplemental Material [file IENZ_A_1925265_SM6539.pdf]

Methoxy and bromo scans on the *N*-(5-methoxyphenyl) ring of methoxybenzenesulfonamides reveal potent cytotoxic compounds with especially high potency against the human breast adenocarcinoma MCF7 cell line.

Myriam González[a,b,c], María Ovejero-Sánchez[b,d,e], Alba Vicente-Blázquez[a,b,c] Manuel Medarde[a,b,c], Rogelio González-Sarmiento[b,d,e] and Rafael Peláez[a,b,c]*

[a]*Laboratorio de Química Orgánica y Farmacéutica. Departamento de Ciencias Farmacéuticas, Facultad de Farmacia, Universidad de Salamanca, Salamanca, Spain;* [b]*Instituto de Investigación Biomédica de Salamanca (IBSAL), Hospital Universitario de Salamanca, Salamanca, Spain;* [c]*Centro de Investigación de Enfermedades Tropicales de la Universidad de Salamanca (CIETUS), Facultad de Farmacia, Universidad de Salamanca, Salamanca, Spain;* [d]*Unidad de Medicina Molecular. Departamento de Medicina, Facultad de Medicina, Universidad de Salamanca, Salamanca, Spain;* [e]*Laboratorio de Diagnóstico en Cáncer Hereditario, Centro de Investigación del Cáncer, Universidad de Salamanca-CSIC, Salamanca, Spain.*

*pelaez@usal.es

Supplementary information

(1) Supplemental Figure 1. Tubulin Polymerization Assay data. IC_{50} calculation.

(2) Supplemental Table 1. Results of the docking studies.

(3) ^{1}H NMR and ^{13}C NMR spectra

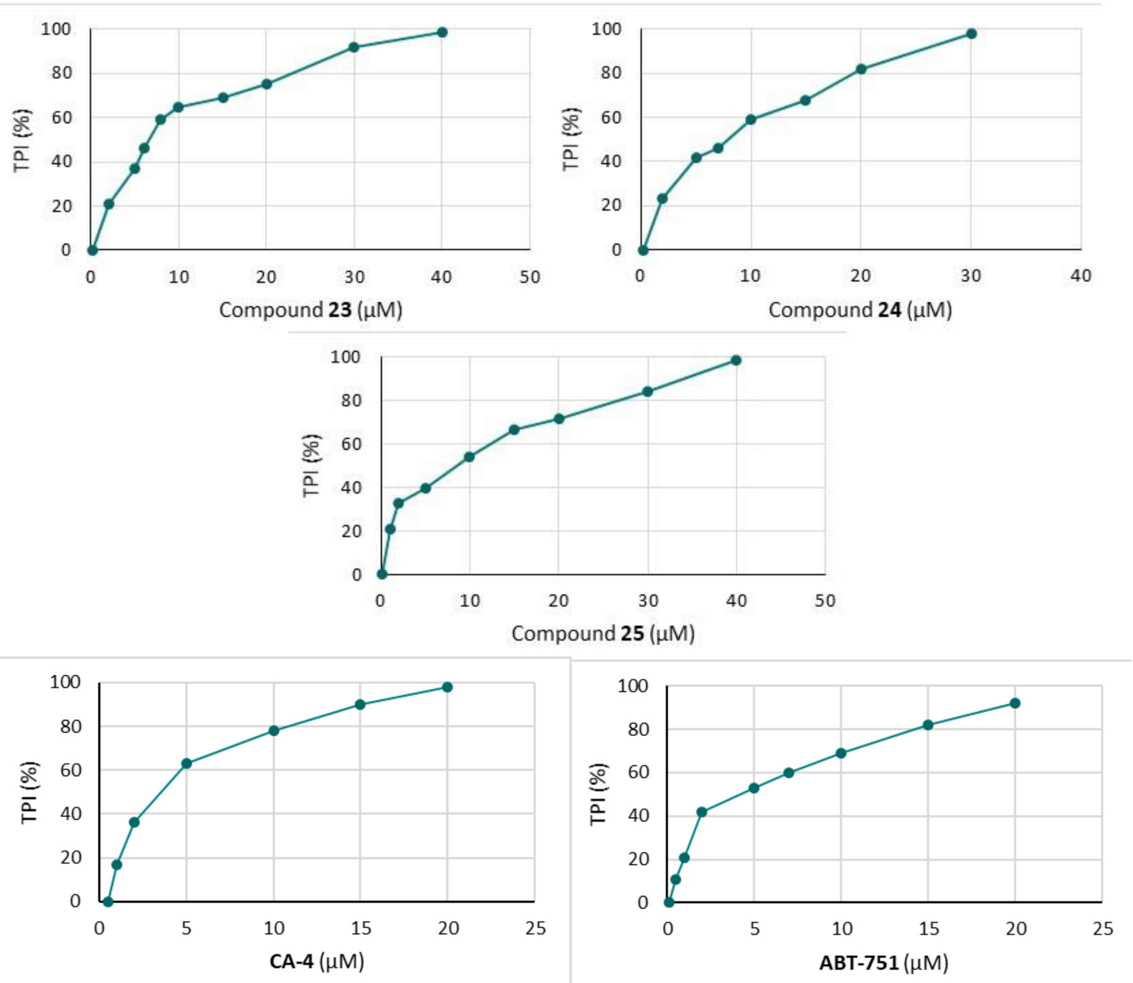

Supplemental Figure 1. Tubulin Polymerization Assay data. IC_{50} calculation. Tubulin Polymerization Inhibition (TPI) percentages are the average of three independent experiments.

Supplemental Table 1. Results of the docking studies.

Series 1	HeLa	MCF-7	HT-29	HT29 Ver	TPI	Conformation	PDB-ID	SITE_OCC	Z-score	PLANTS	E_AD
**8a M327**	497	99	495	293	>10						
35DM4Br_MeNSO2_4MeOPh_M327_5GON_conf_01						cisoid	5GON	AB	1,0	-85,5	-25,5
35DM4Br_MeNSO2_4MeOPh_M327_5LYJ_90						cisoid	5LYJ	AB	1,0		
**8b M327B**	>1000	>1000	>1000	-	>10						
35DM2Br_MeNSO2_4MeOPh_M327B_5CB4_conf_02						transoid out	5CB4	AB	1,0	-79,5	-23,4
35DM2Br_MeNSO2_4MeOPh_M327B_5LYJ_78						transoid out	5LYJ	AB	1,0		
**9a M335**	417	-	610	-	>10						
35DM4Br_EtNSO2_4MeOPh_M335_5GON_conf_01						cisoid out	5GON	AB	1,0	-87,7	-25,2
35DM4Br_EtNSO2_4MeOPh_M335_5LYJ_60						transoid out	5LYJ	AB	1,0		
**9b M335B**	>1000	>1000	>1000	-	>10						
35DM2Br_EtNSO2_4MeOPh_M335B_5XKH_39						transoid out	5XKH	AB	1,0		
35DM2Br_EtNSO2_4MeOPh_M335B_6GJ4_conf_01						cisoid out	6GJ4	AB	1,0	-81,4	-23,1
**10a M326**	310	335	450	533	>10						
35DM4Br_BnNSO2_4MeOPh_M326_6BS2_78						transoid out	6BS2	ABC	1,0		
35DM4Br_BnNSO2_4MeOPh_M326_5GON_conf_01						transoid out	5GON	AB	1,0	-94,5	-25,3
**10b M326B**	>1000	>1000	>1000	>1000	>10						
35DM2Br_BnNSO2_4MeOPh_M326B_5XAF_31						transoid out	5XAF	AB	1,0		
35DM2Br_BnNSO2_4MeOPh_M326B_5GON_conf_01						transoid out	5GON	AB	0,9	-95,6	-25,6
**10c M326C**	>1000	>1000	>1000	>1000	>10						
35DM24Br2_BnNSO2_4MeOPh_M326C_5GON_conf_01						transoid out	5GON	AB	1,0	-90,3	-23,6
35DM24Br2_BnNSO2_4MeOPh_M326C_5XAF_38						transoid out	5XAF	AB	1,0		

Series 2	HeLa	MCF-7	HT-29	HT29 Ver	TPI	Conformation	PDB-ID	SITE_OCC	Z-score	PLANTS	E_AD
**11 M8**	>1000	>1000	>1000	>1000	>10						
34DM_HNSO2_4MeOPh_M8_5GON_conf_02						transoid	5GON	AB	0,8	-76,7	-23,1
34DM_HNSO2_4MeOPh_M8_6BS2_47						transoid	6BS2	AB	0,7		
**12 M38**	>1000	>1000	>1000	>1000	>10						
34DM_MeNSO2_4MeOPh_M38_5GON_conf_01						cisoid reverse	5GON	AB	0,5	-83,1	-25,0
34DM_MeNSO2_4MeOPh_M38_5LYJ_83						cisoid reverse	5LYJ	AB	0,5		
**14 M236**	557	-	907	-	>10						
34DM6Br_MeNSO2_4MeOPh_M236_6GJ4_conf_01						cisoid	6GJ4	AB	1,0	-75,9	-22,2
34DM6Br_MeNSO2_4MeOPh_M236_5XKF_78						cisoid	5XKF	BC	1,0		
**15 M354**	313	660	365	500	>10						
34DM6Br_EtNSO2_4MeOPh_M354_5GON_conf_01						cisoid	5GON	AB	1,0	-90,4	-26,1
34DM6Br_EtNSO2_4MeOPh_M354_6D88_86						cisoid	6D88	AB	1,0		
34DM6Br_EtNSO2_4MeOPh_M354_5LYJ_conf_01						transoid	5LYJ	ABC	1,0	-88,7	-25,6
34DM6Br_EtNSO2_4MeOPh_M354_5XKG_6						transoid	5XKG	BC	1,0		
**16 M237**	407	575	360	750	>10						
34DM6Br_NAcNSO2_4MeOPh_M237_6BRY_55						transoid out	6BRY	AB	1,0		
34DM6Br_NAcNSO2_4MeOPh_M237_5CB4_conf_01						transoid out	5CB4	AB	1,0	-79,6	-21,5
34DM6Br_NAcNSO2_4MeOPh_M237_5XKF_22						transoid in	5XKF	ABC	1,0		
34DM6Br_NAcNSO2_4MeOPh_M237_5XLZ_conf_03						transoid in	5XLZ	AB	1,0	-80,2	-21,7
**17 M355**	>1000	>1000	>1000	>1000	>10						
34DM6Br_EtOAcNSO2_4MeOPh_M355_5CB4_conf_02						transoid out	5CB4	AB	1,0	-91,2	-23,2
34DM6Br_EtOAcNSO2_4MeOPh_M355_5XKG_79						transoid out	5XKG	AB	1,0		
**18 M356**	>1000	>1000	>1000	>1000	>10						
34DM6Br_BnNSO2_4MeOPh_M356_6BR1_91						transoid out	6BR1	AB	1,0		
34DM6Br_BnNSO2_4MeOPh_M356_5GON_conf_02						transoid out	5GON	AB	1,0	-101,7	-27,6
**21 M76**	227	350	187	187	>10						
25DM_HNSO2_4MeOPh_M76_5XLZ_conf_02						transoid	5XLZ	AB	-79,9	-67,9	
25DM_HNSO2_4MeOPh_M76_6BRY_65						transoid	6BRY	AB			-8,0

**22 M89**	177	153	250	305	>10						
25DM_MeNSO2_4MeOPh_M89_6BRY_4						transoid	6BRY	AB	0,5		
25DM_MeNSO2_4MeOPh_M89_5XLZ_conf_02						transoid	5XLZ	AB	0,5	-76,8	-22,8
**23 M98**	45	25	72	30	6.9						
25DM4Br_HNSO2_4MeOPh_M98_5GON_conf_03							5GON	AB	0,5	-77,2	-22,9
25DM4Br_HNSO2_4MeOPh_M98_5XAF_22						transoid	5XAF	BC	0,5		
**24 M105**	33	19	123	160	7,6						
25DM4Br_MeNSO2_4MeOPh_M105_6BS2_conf_01							6BS2	AB	0,7	-76,6	-22,4
25DM4Br_MeNSO2_4MeOPh_M105_5LYJ_59							5LYJ	AB	0,7		
**25 M176**	36	86	97	85	7,6						
25DM4Br_NAcNSO2_4MeOPh_M176_5XLZ_conf_01						transoid	5XLZ	AB	0,8	-81,1	-22,0
25DM4Br_NAcNSO2_4MeOPh_M176_5LYJ_89						transoid	5LYJ	AB	0,8		
**26 M177**	577	700	2100	867	>10						
25DM4Br_EtOAcSO2_4MeOPh_M177_5XAF_20						transoid	5XAF	AB	1,0		
25DM4Br_EtOAcSO2_4MeOPh_M177_5CB4_conf_01						transoid	5CB4	AB	1,0	-89,9	-22,8
**27 M98**	377	220	525	170	>10						
25DM4Br_EtOCONSO2_4MeOPh_M98_5XKF_22						transoid	5XKF	AB	1,0		
25DM4Br_EtOAcNSO2_4MeOPh_M98_5GON_conf_02						transoid	5GON	AB	1,0	-90,0	-23,7
**27 M177**	377	220	525	170	>10						
25DM4Br_EtOCONSO2_4MeOPh_M179_5XKF_62						transoid	5XKF	AB	1,0		
25DM4Br_EtOCONSO2_4MeOPh_M179_5GON_conf_03						transoid	5GON	AB	0,9	-87,5	-22,9
**28 M178R**	495	-	860	-	>10						
25DM4Br_OxirMeNSO2_4MeOPh_M178_5XLZ_conf_01						transoid	5XLZ	AB	1,0	-88,5	-24,2
25DM4Br_OxirMeNSO2_4MeOPh_M178_6D88_16						transoid	6D88	AB	1,0		
**28 M178S**											
25DM4Br_OxirMeNSO2_4MeOPh_M178S_6BRY_57						cisoid	6BRY	AB	1,0		
25DM4Br_OxirMeNSO2_4MeOPh_M178S_5XAF_conf_01						cisoid	5XAF	AB	1,0	-86,4	-23,5
**29 M190**	>1000	>1000	>1000	>1000	>10						
25DM4Br_HOAcSO2_4MeOPh_M190_6D88_59						transoid	6D88	AB	1,0		
25DM4Br_HOAcSO2_4MeOPh_M190_5XLZ_conf_01						transoid	5XLZ	AB	1,0	-86,7	-22,9
**29 M190an**											
25DM4Br_OAcSO2_4MeOPh_M190_5LYJ_40						transoid	5LYJ	AB	1,0		
25DM4Br_OAcSO2_4MeOPh_M190_6BS2_conf_01						cisoid	6BS2	AB	1,0	-88,3	-24,1
25DM4Br_OAcSO2_4MeOPh_M190_6BRY_7						cisoid	6BRY	AB	0,9		
25DM4Br_OAcSO2_4MeOPh_M190_5XLZ_conf_02						transoid	5XLZ	AB	0,9	-83,4	-22,5
**32 M350**	410	580	360	553	>10						
245TM_HNSO2_4MeOPh_M350_5XAF_conf_01						cisoid	5XAF	AB	0,9	-74,6	-21,0
245TM_HNSO2_4MeOPh_M350_6BRY_36						transoid	6BRY	AB	0,9		
**33 M361**	413	99	500	577	>10						
245TM_MeNSO2_4MeOPh_M361_5XKG_6						transoid	5XKG	ABC	1,0		
245TM_MeNSO2_4MeOPh_M361_5XLZ_conf_02						transoid	5XLZ	AB	1,0	-72,8	-20,1
**34 M362**	313	715	500	1070	>10						
245TM_EtNSO2_4MeOPh_M362_6D88_54						transoid	6D88	ABC	1,0		
245TM_EtNSO2_4MeOPh_M362_5XLZ_conf_02						transoid	5XLZ	AB	0,9	-79,0	-21,3
**35 M365**	500	67	365	415	>10						
245TM_NAcNSO2_4MeOPh_M365_5XKG_90						transoid	5XKG	BC	1,0		
245TM_NAcNSO2_4MeOPh_M365_5XLZ_conf_03						transoid	5XLZ	AB	0,9	-78,2	-20,1
**36 M364**	>1000	>1000	>1000	-	>10						
245TM_EtOAcNSO2_4MeOPh_M364_5CB4_conf_01						cisoid	5CB4	AB	1,0	-86,7	-20,8
245TM_EtOAcNSO2_4MeOPh_M364_5XAF_42						transoid	5XAF	AB	1,0		
**37 M363**	833	-	1250	-	>10						
245TM_BnNSO2_4MeOPh_M363_6BR1_14						transoid	6BR1	AB	0,99361702		
245TM_BnNSO2_4MeOPh_M363_5GON_conf_01						transoid	5GON	AB	0,92598817	-91,3968	-23,3645

**Series 3**	**HeLa**	**MCF-7**	**HT-29**	**HT29 Ver**	**TPI**	**Conformation**	**PDB-ID**	**SITE_OCC**	**Z-score**	**PLANTS**	**E_AD**
**21 M76**	227	350	187	187							

25DM_HNSO2_4MeOPh_M76_5XLZ_conf_02						transoid	5XLZ	AB	-79,9		
25DM_HNSO2_4MeOPh_M76_6BRY_65						transoid	6BRY	AB		-8,0	
**22 M89**	177	153	250	305							
25DM_MeNSO2_4MeOPh_M89_6BRY_4						transoid	6BRY	AB	0,5		
25DM_MeNSO2_4MeOPh_M89_5XLZ_conf_02						transoid	5XLZ	AB	0,5	-22,8	
**23 M98**	45	25	72	30							
25DM4Br_HNSO2_4MeOPh_M98_5GON_conf_03							5GON	AB	0,5	-22,9	
25DM4Br_HNSO2_4MeOPh_M98_5XAF_22						transoid	5XAF	BC	0,5		
**24 M105**	33	19	123	160							
25DM4Br_MeNSO2_4MeOPh_M105_6BS2_conf_01							6BS2	AB	0,7	-22,4	
25DM4Br_MeNSO2_4MeOPh_M105_5LYJ_59							5LYJ	AB	0,7		
**25 M176**	36	86	97	85							
25DM4Br_NAcNSO2_4MeOPh_M176_5XLZ_conf_01						transoid	5XLZ	AB	0,8	-22,0	
25DM4Br_NAcNSO2_4MeOPh_M176_5LYJ_89						transoid	5LYJ	AB	0,8		
**26 M177**	577	700	2100	867							
25DM4Br_EtOAcSO2_4MeOPh_M177_5XAF_20						transoid	5XAF	AB	1,0		
25DM4Br_EtOAcSO2_4MeOPh_M177_5CB4_conf_01						transoid	5CB4	AB	1,0	-22,8	
**27 M98**	377	220	525	170							
25DM4Br_EtOCONSO2_4MeOPh_M98_5XKF_22						transoid	5XKF	AB	1,0		
25DM4Br_EtOAcNSO2_4MeOPh_M98_5GON_conf_02						transoid	5GON	AB	1,0	-23,7	
**27 M177**	377	220	525	170							
25DM4Br_EtOCONSO2_4MeOPh_M179_5XKF_62						transoid	5XKF	AB	1,0		
25DM4Br_EtOCONSO2_4MeOPh_M179_5GON_conf_03						transoid	5GON	AB	0,9	-22,9	
**28 M178R**	495	-	860	-							
25DM4Br_OxirMeNSO2_4MeOPh_M178_5XLZ_conf_01						transoid	5XLZ	AB	1,0	-24,2	
25DM4Br_OxirMeNSO2_4MeOPh_M178_6D88_16						transoid	6D88	AB	1,0		
**28 M178S**											
25DM4Br_OxirMeNSO2_4MeOPh_M178S_6BRY_57						cisoid	6BRY	AB	1,0		
25DM4Br_OxirMeNSO2_4MeOPh_M178S_5XAF_conf_01						cisoid	5XAF	AB	1,0	-23,5	
**29 M190**	>1000	>1000	>1000	>1000							
25DM4Br_HOAcSO2_4MeOPh_M190_6D88_59						transoid	6D88	AB	1,0		
25DM4Br_HOAcSO2_4MeOPh_M190_5XLZ_conf_01						transoid	5XLZ	AB	1,0	-22,9	
**29 M190an**											
25DM4Br_OAcSO2_4MeOPh_M190_5LYJ_40						transoid	5LYJ	AB	1,0		
25DM4Br_OAcSO2_4MeOPh_M190_6BS2_conf_01						cisoid	6BS2	AB	1,0	-24,1	
25DM4Br_OAcSO2_4MeOPh_M190_6BRY_7						cisoid	6BRY	AB	0,9		
25DM4Br_OAcSO2_4MeOPh_M190_5XLZ_conf_02						transoid	5XLZ	AB	0,9	-22,5	

**Series 4**	**HeLa**	**MCF-7**	**HT-29**	**HT29 Ver**	**TPI**	**Conformation**	**PDB-ID**	**SITE_OCC**	**Z-score**	**PLANTS**	**E_AD**
**32 M350**	410	580	360	553							
245TM_HNSO2_4MeOPh_M350_5XAF_conf_01						cisoid	5XAF	AB	0,9	-21,0	
245TM_HNSO2_4MeOPh_M350_6BRY_36						transoid	6BRY	AB	0,9		
**33 M361**	413	99	500	577							
245TM_MeNSO2_4MeOPh_M361_5XKG_6						transoid	5XKG	ABC	1,0		
245TM_MeNSO2_4MeOPh_M361_5XLZ_conf_02						transoid	5XLZ	AB	1,0	-20,1	
**34 M362**	313	715	500	1070							
245TM_EtNSO2_4MeOPh_M362_6D88_54						transoid	6D88	ABC	1,0		
245TM_EtNSO2_4MeOPh_M362_5XLZ_conf_02						transoid	5XLZ	AB	0,9	-21,3	
**35 M365**	500	67	365	415							
245TM_NAcNSO2_4MeOPh_M365_5XKG_90						transoid	5XKG	BC	1,0		
245TM_NAcNSO2_4MeOPh_M365_5XLZ_conf_03						transoid	5XLZ	AB	0,9	-20,1	
**36 M364**	>1000	>1000	>1000	-							
245TM_EtOAcNSO2_4MeOPh_M364_5CB4_conf_01						cisoid	5CB4	AB	1,0	-20,8	
245TM_EtOAcNSO2_4MeOPh_M364_5XAF_42						transoid	5XAF	AB	1,0		
**37 M363**	833	-	1250	-							

245TM_BnNSO2_4MeOPh_M363_6BR1_14	transoid	6BR1	AB	0,99361702	
245TM_BnNSO2_4MeOPh_M363_5GON_conf_01	transoid	5GON	AB	0,92598817	-23,3645

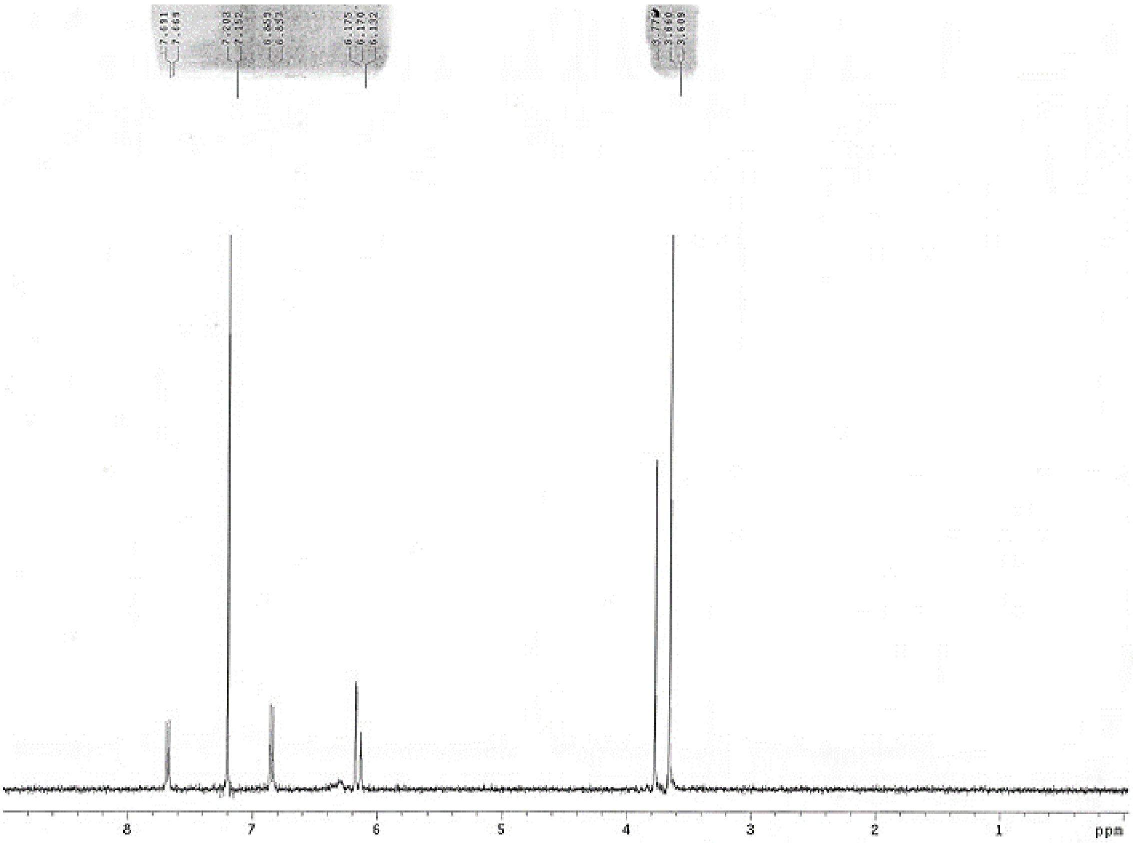

Fig. 1a: Compound **1** ^{1}H NMR spectra.

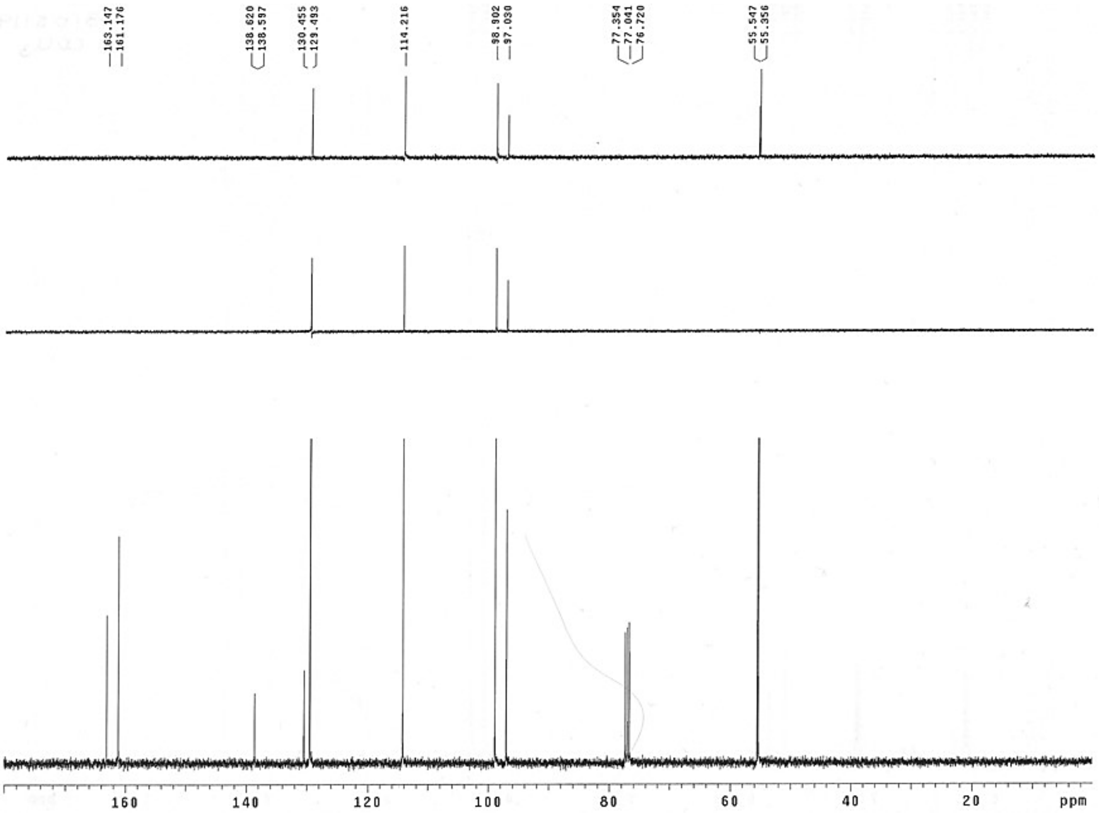

Fig. 1b: Compound **1** ^{13}C NMR spectra.

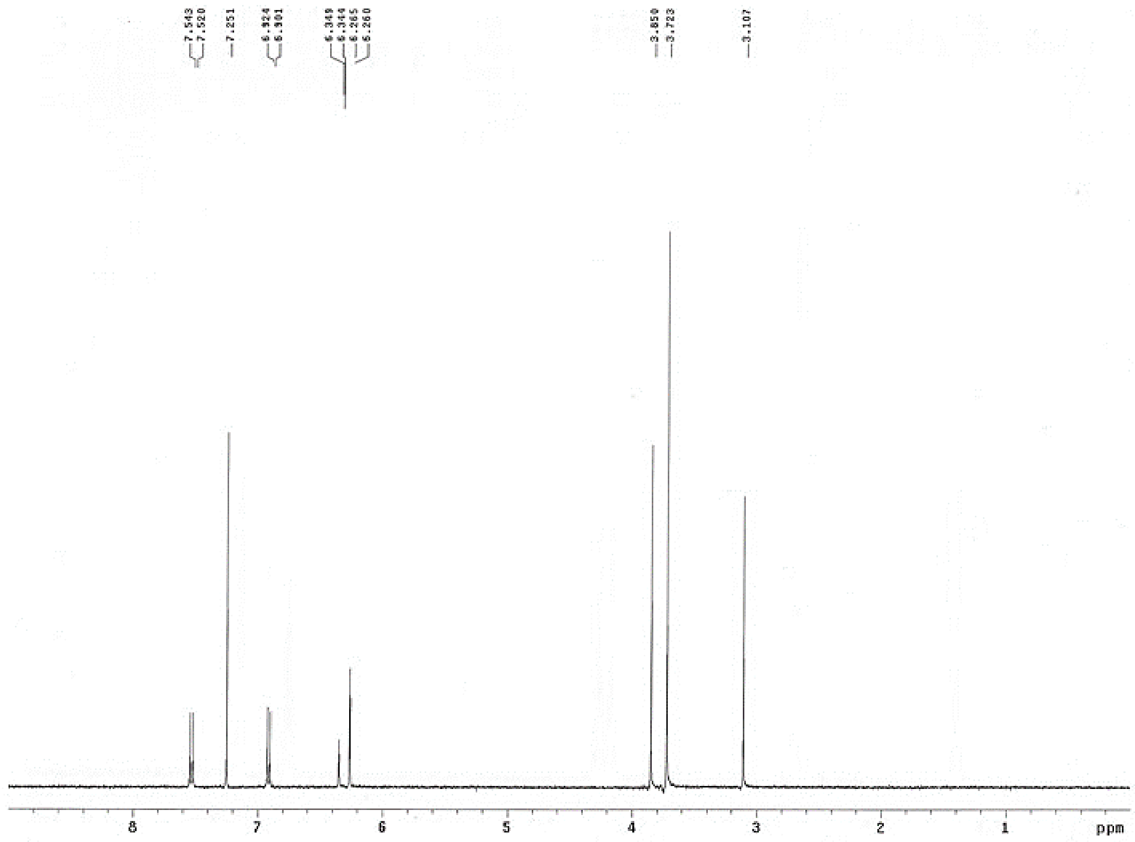

Fig. 2a: Compound **2** ^{1}H NMR spectra.

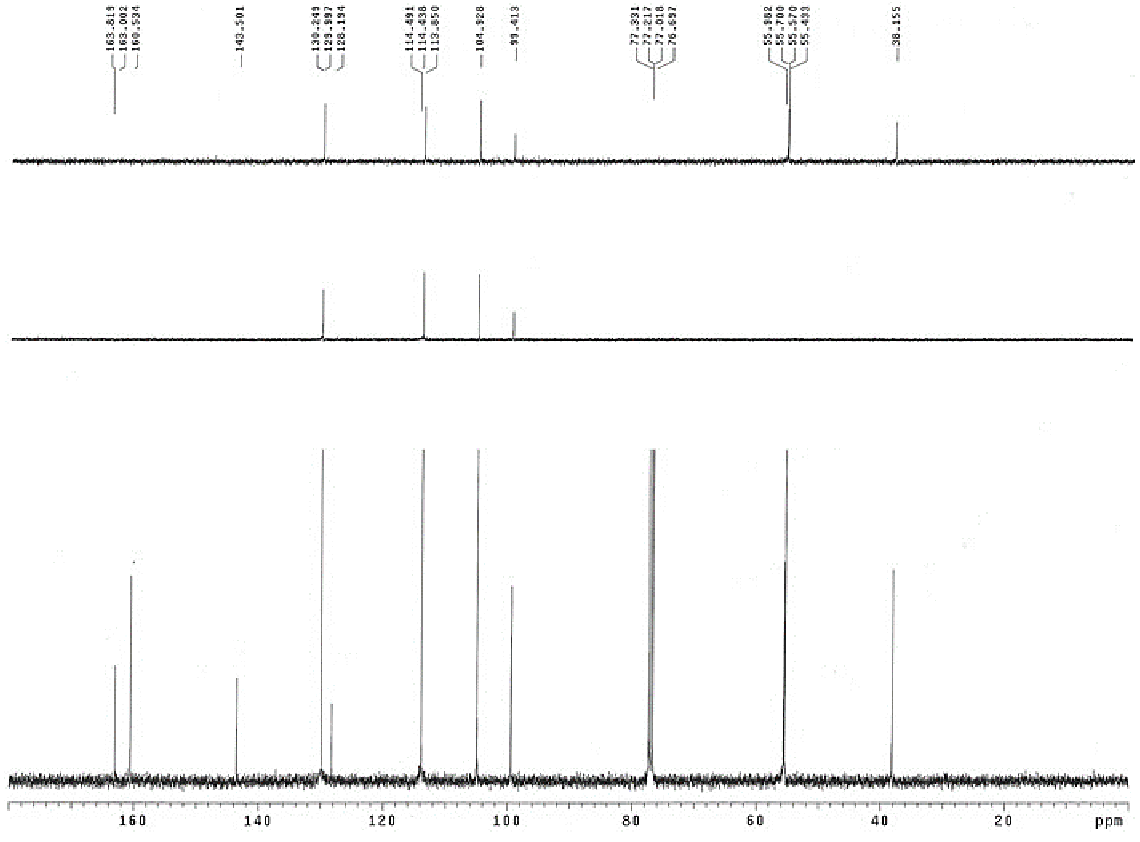

Fig. 2b: Compound **2** ^{13}C NMR spectra.

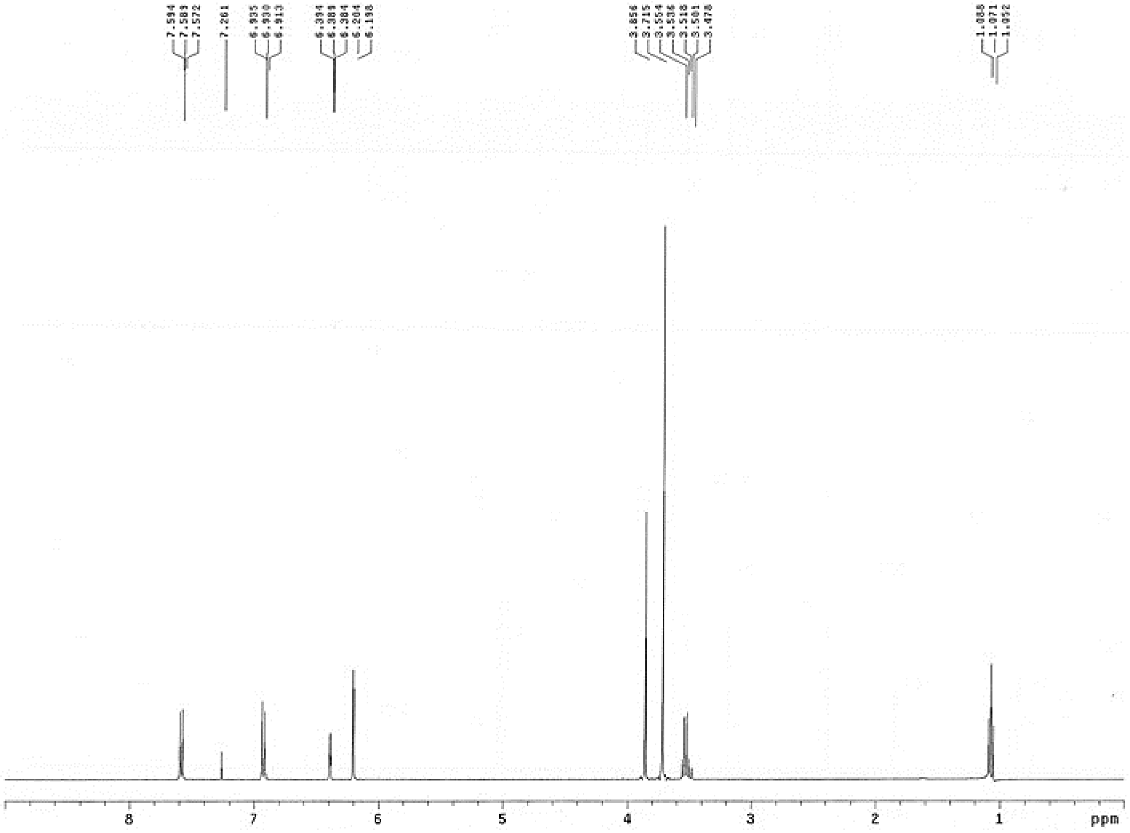

Fig. 3a: Compound **3** ^{1}H NMR spectra.

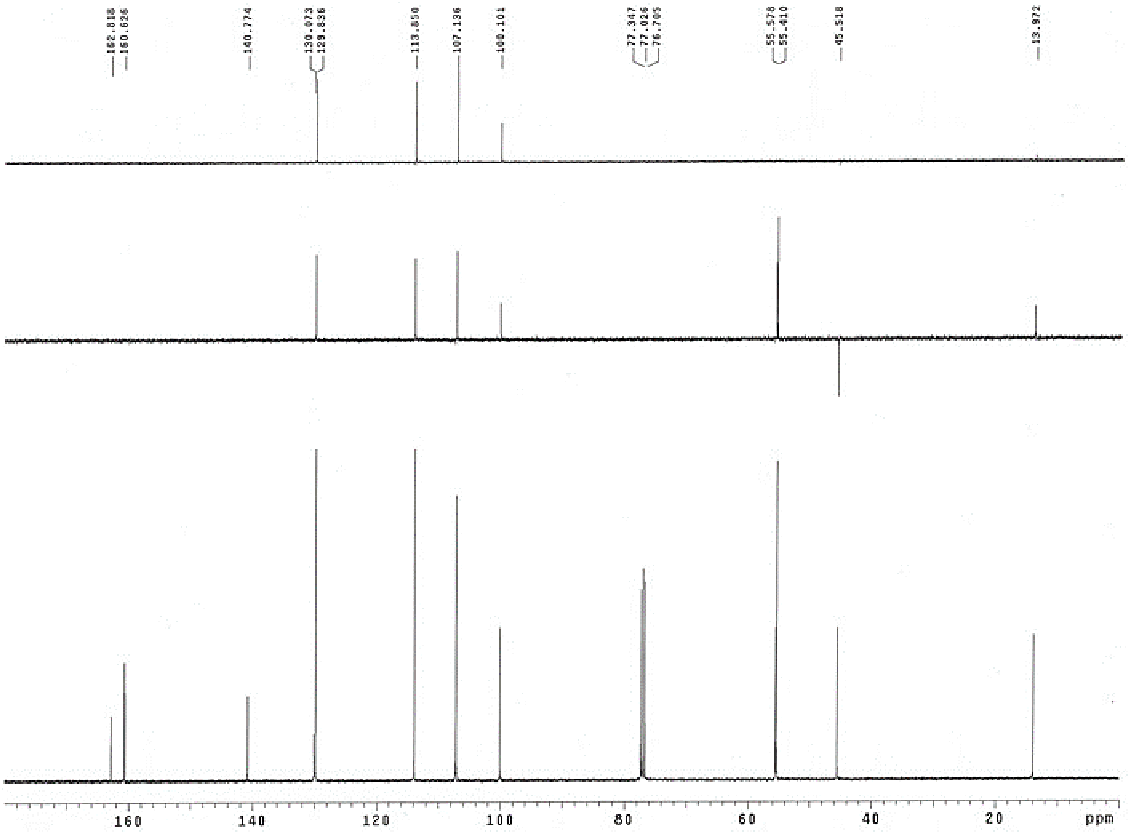

Fig. 3b: Compound **3** ^{13}C NMR spectra.

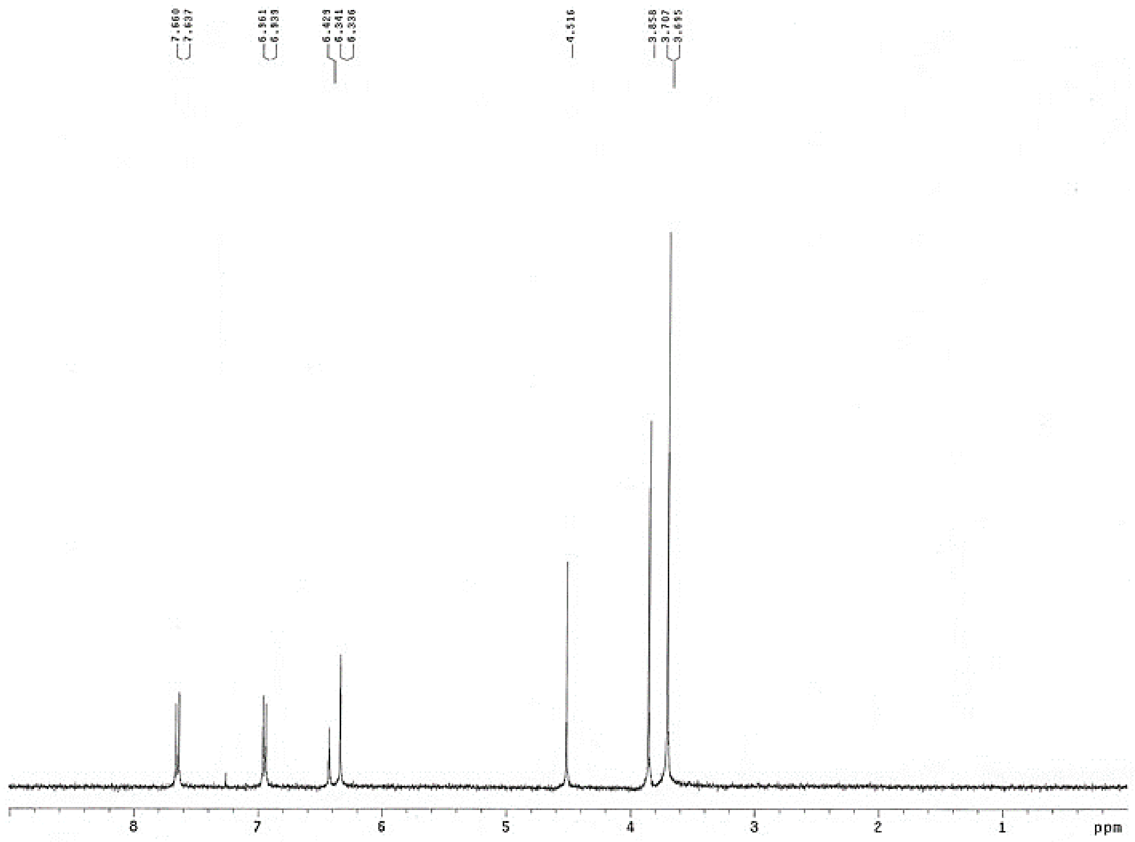

Fig. 4a: Compound **4** ^{1}H NMR spectra.

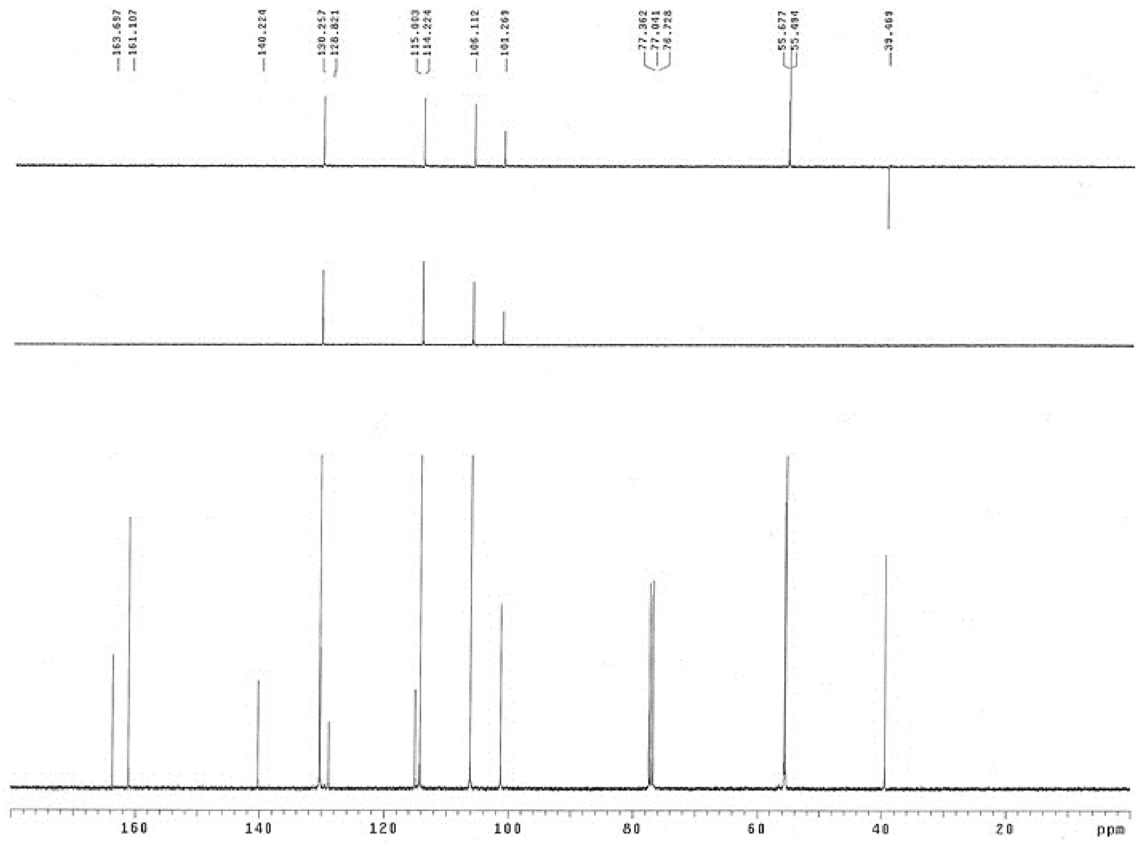

Fig. 4b: Compound **4** ^{13}C NMR spectra.

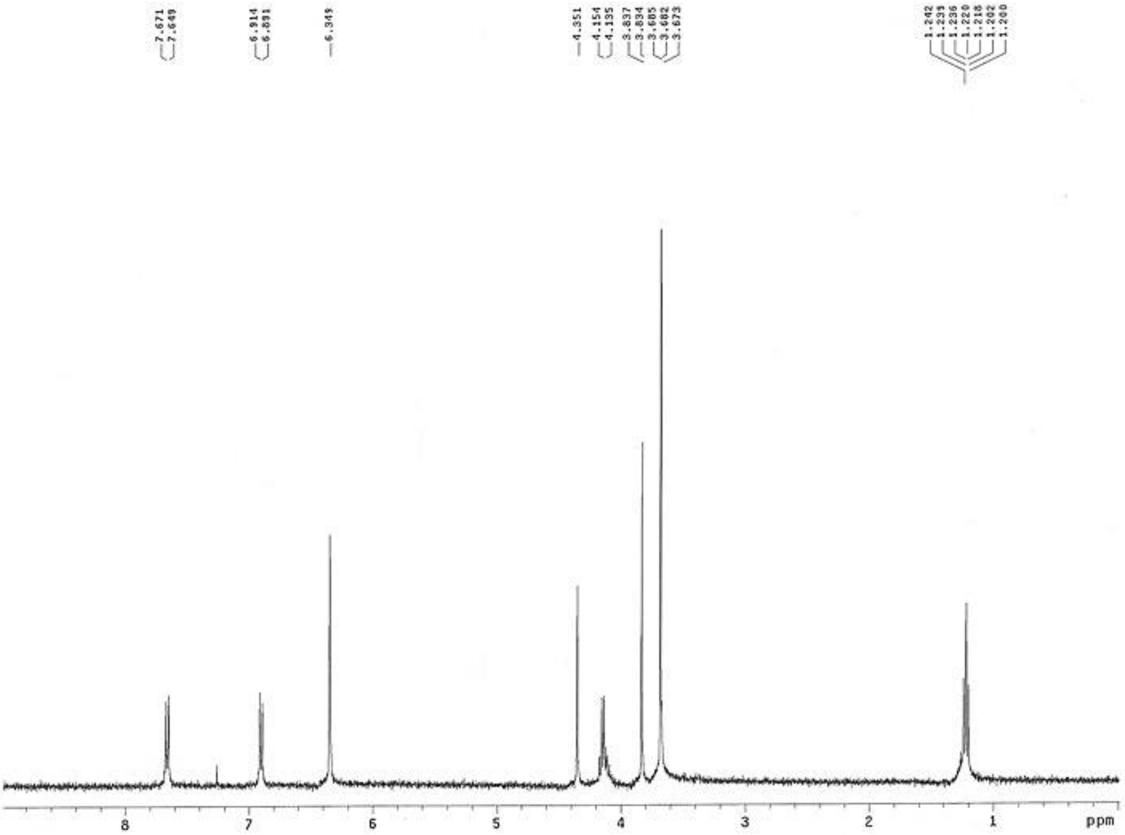

Fig. 5a: Compound **5** ^{1}H NMR spectra.

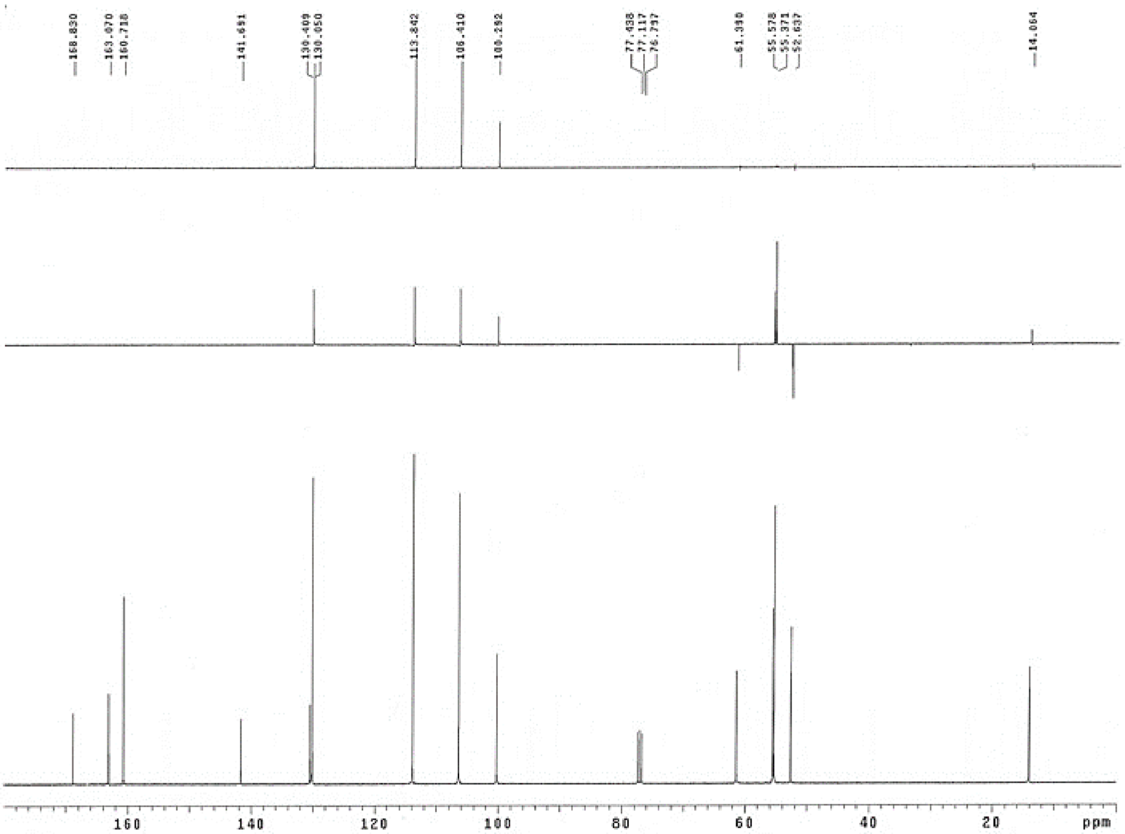

Fig. 5b: Compound **5** ^{13}C NMR spectra.

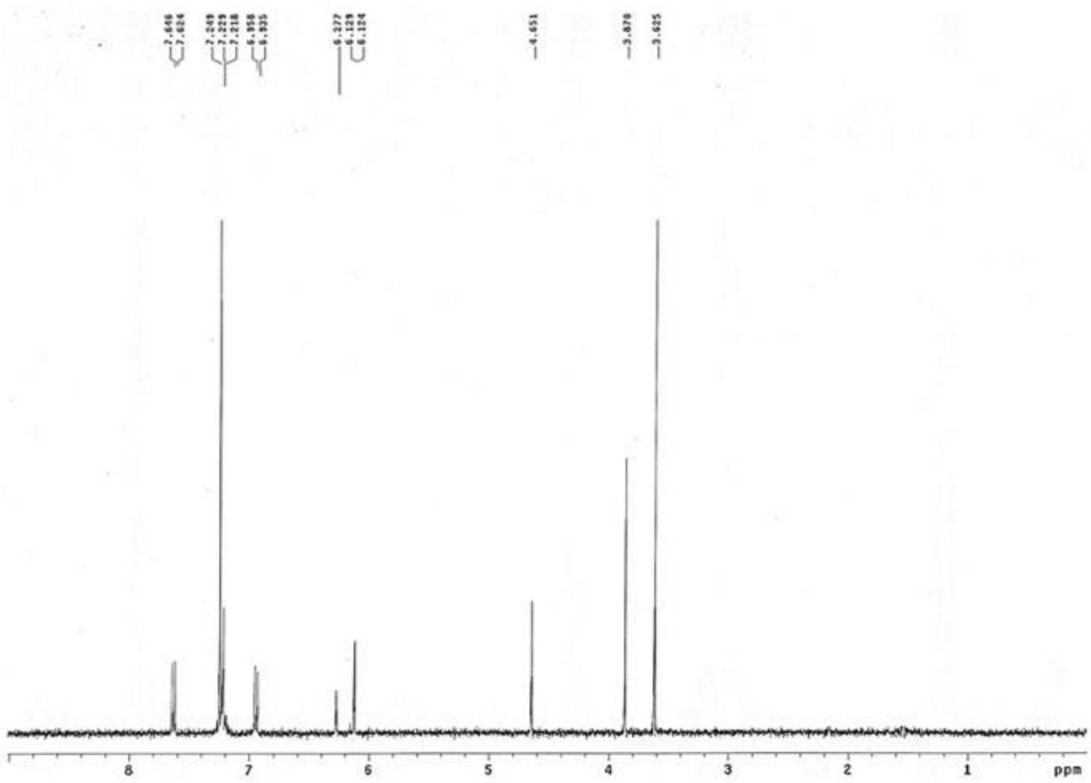

Fig. 6a: Compound **6** ^{1}H NMR spectra.

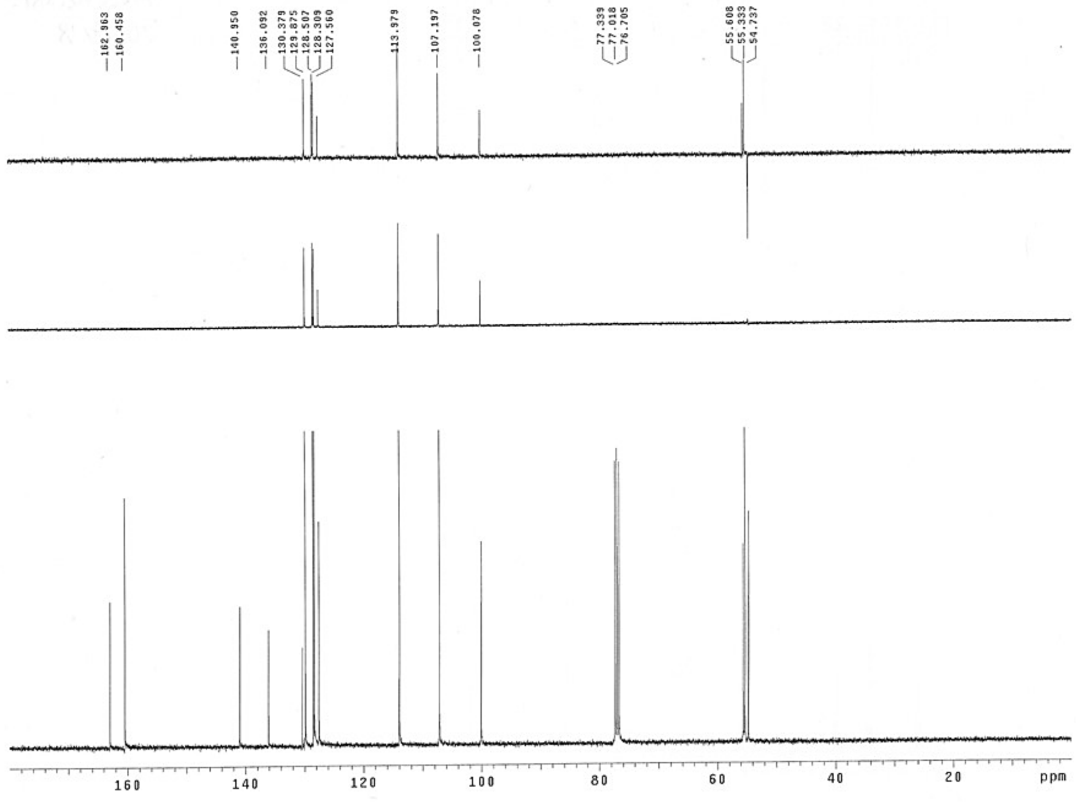

Fig. 6b: Compound **6** ^{13}C NMR spectra.

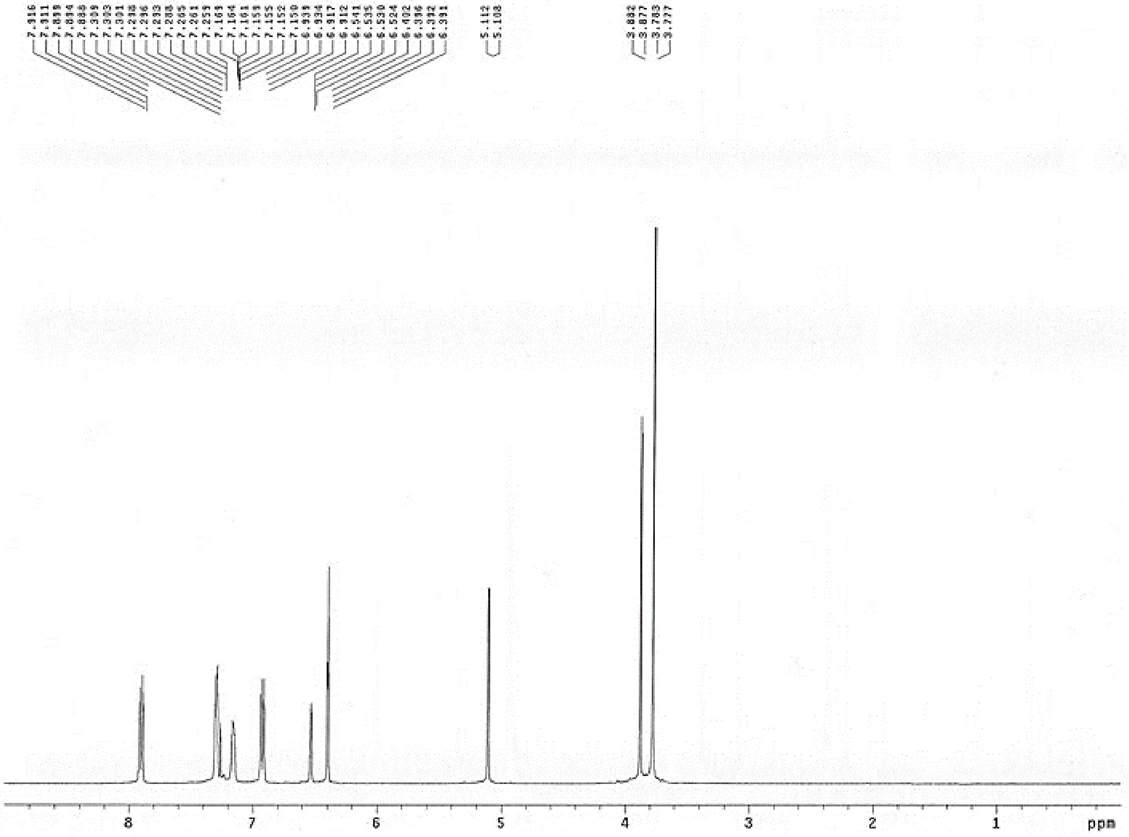

Fig. 7a: Compound **7** ^{1}H NMR spectra.

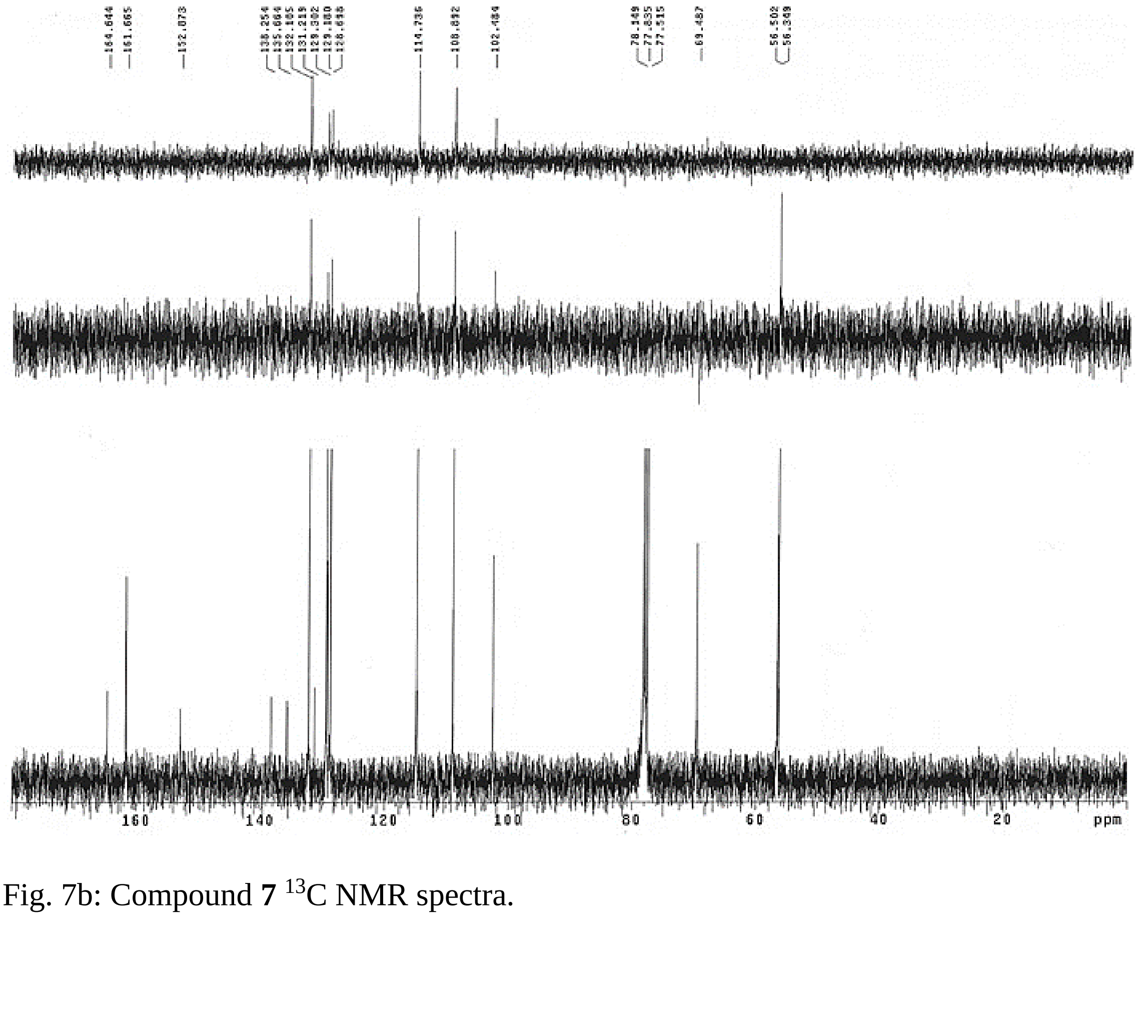

Fig. 7b: Compound **7** ^{13}C NMR spectra.

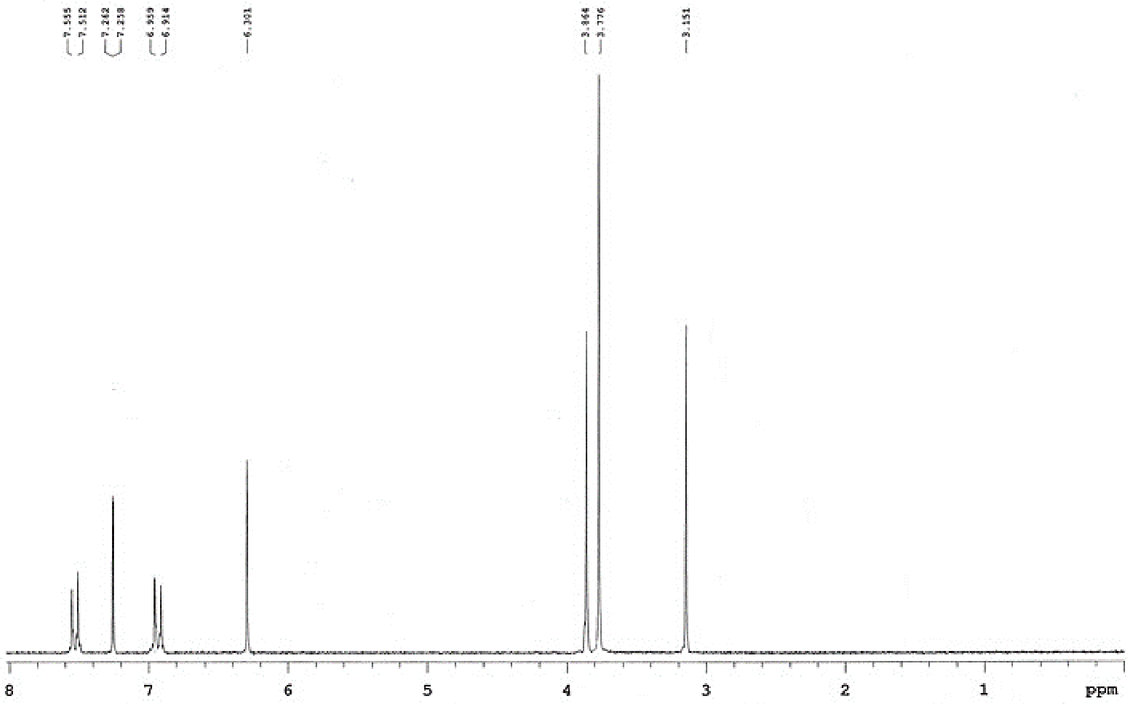

Fig. 8a: Compound **8a** ^{1}H NMR spectra.

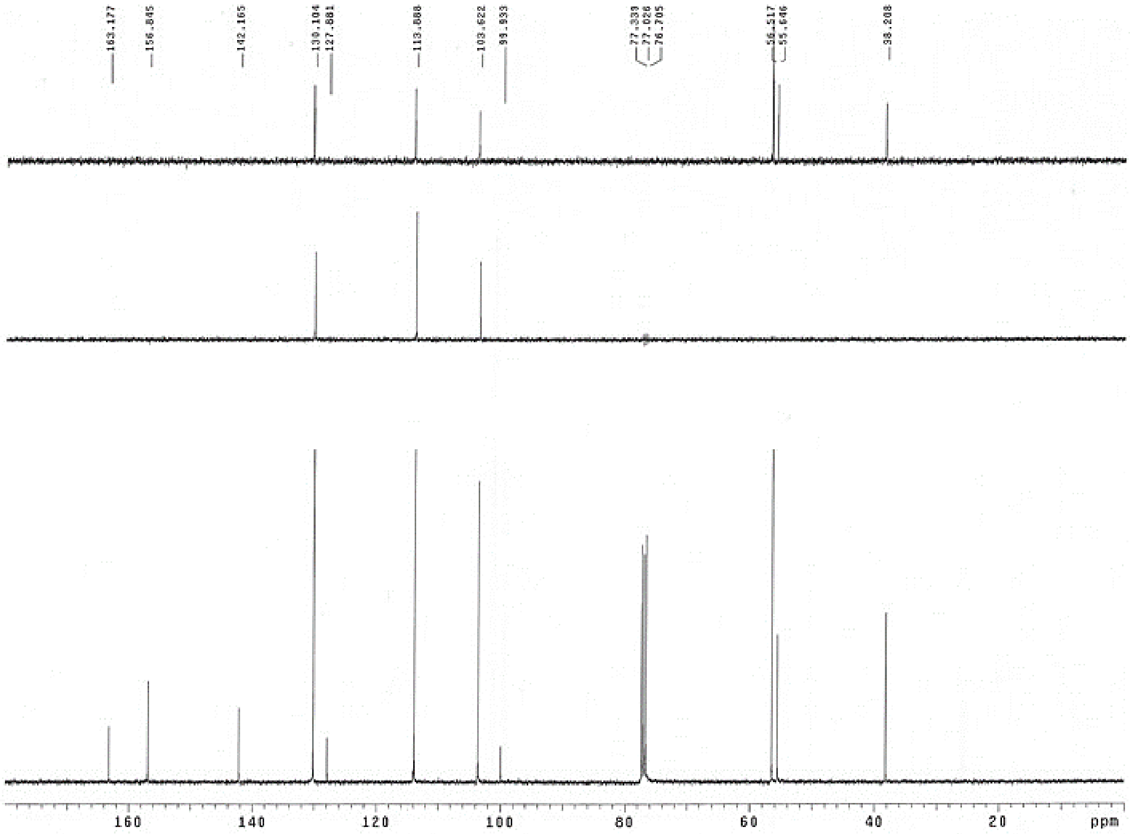

Fig. 8b: Compound **8a** ^{13}C NMR spectra.

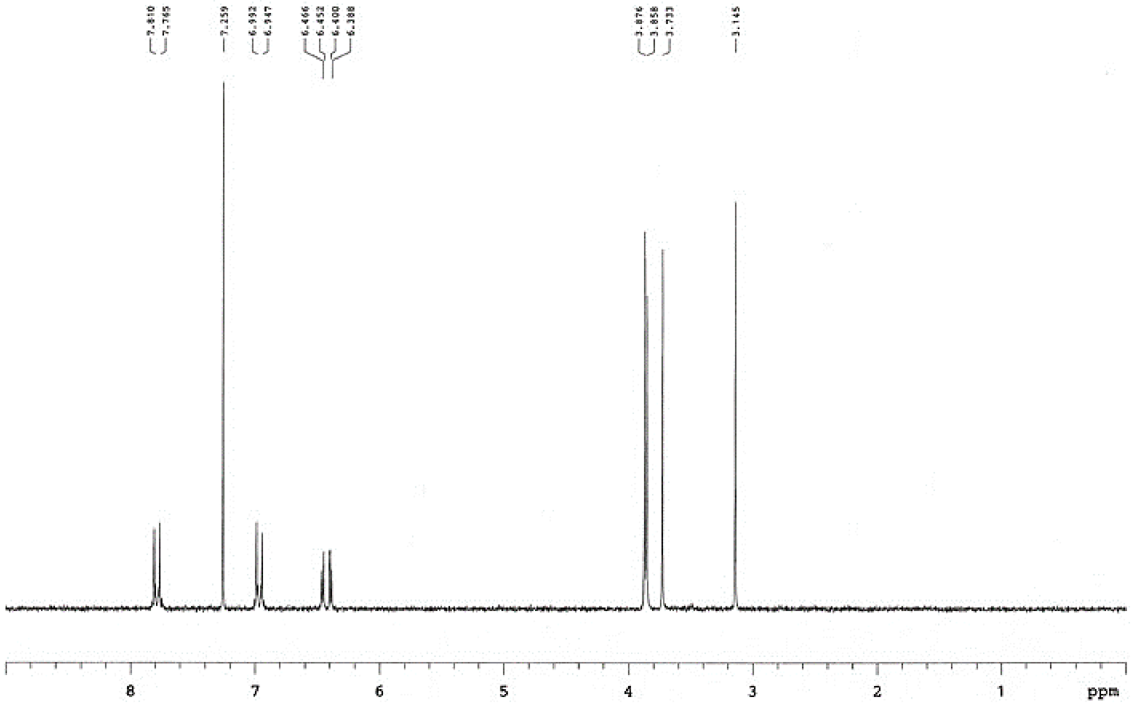

Fig. 9a: Compound **8b** ^{1}H NMR spectra.

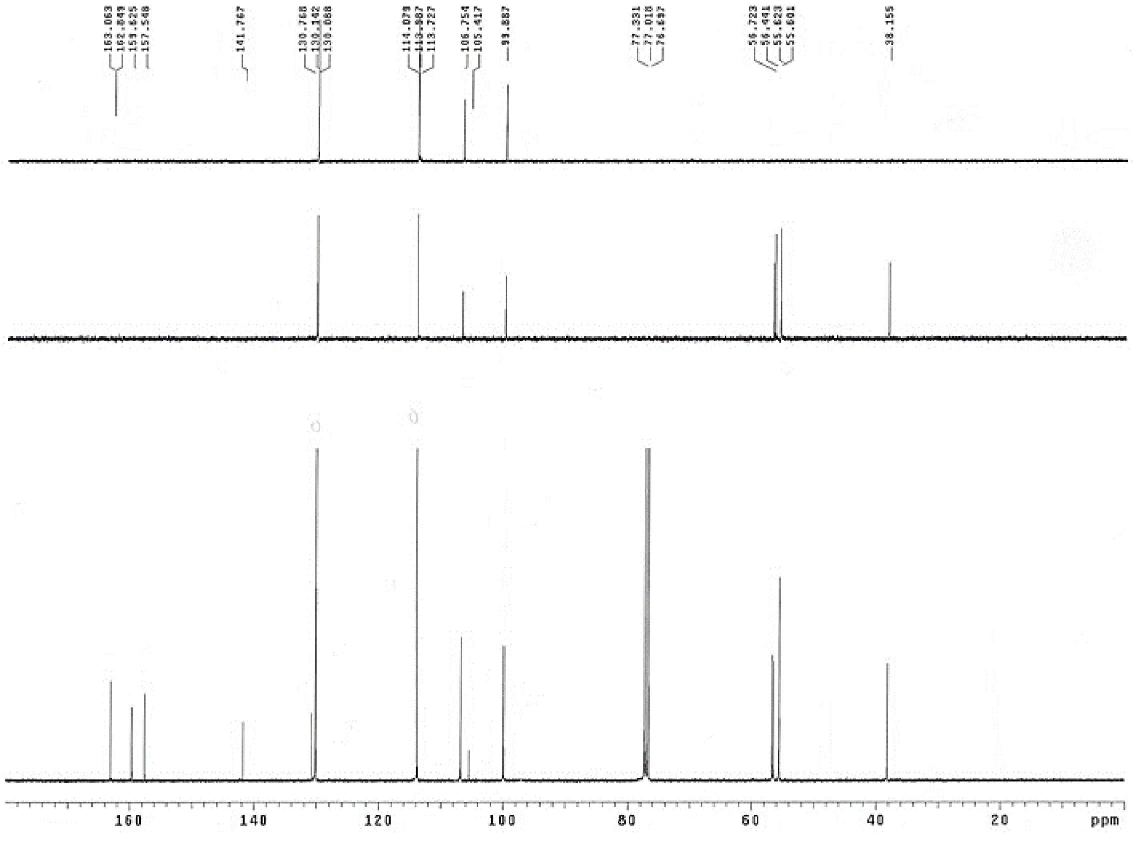

Fig. 9b: Compound **8b** ^{13}C NMR spectra.

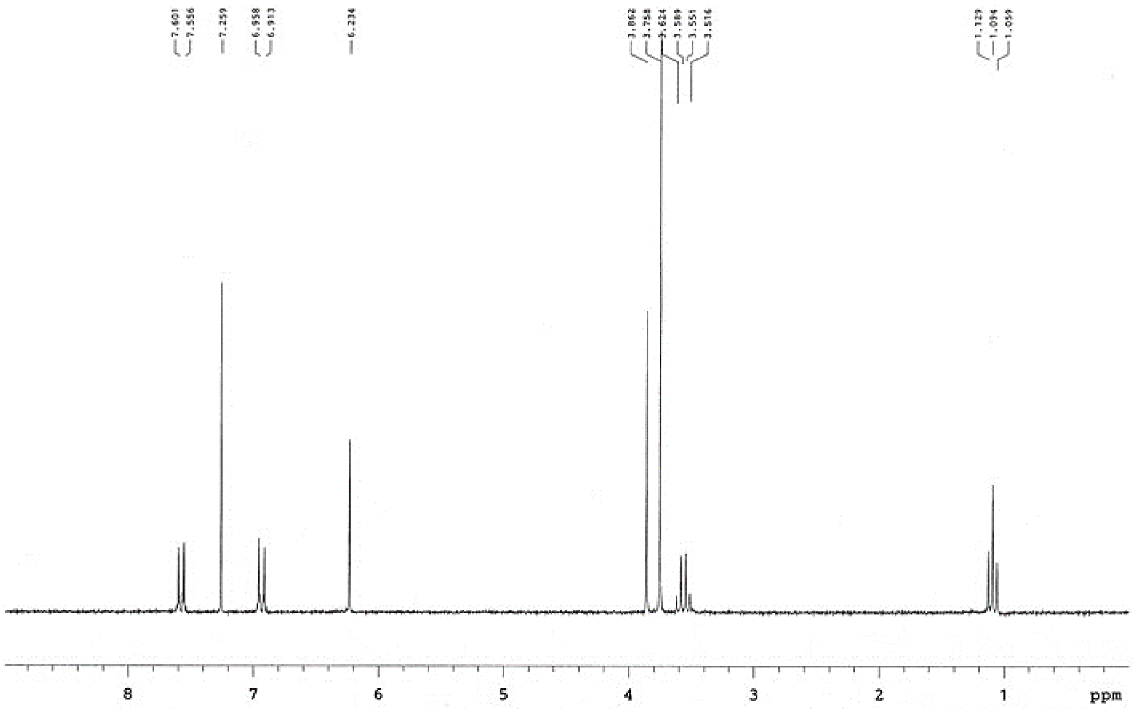

Fig. 10a: Compound **9a** ^{1}H NMR spectra.

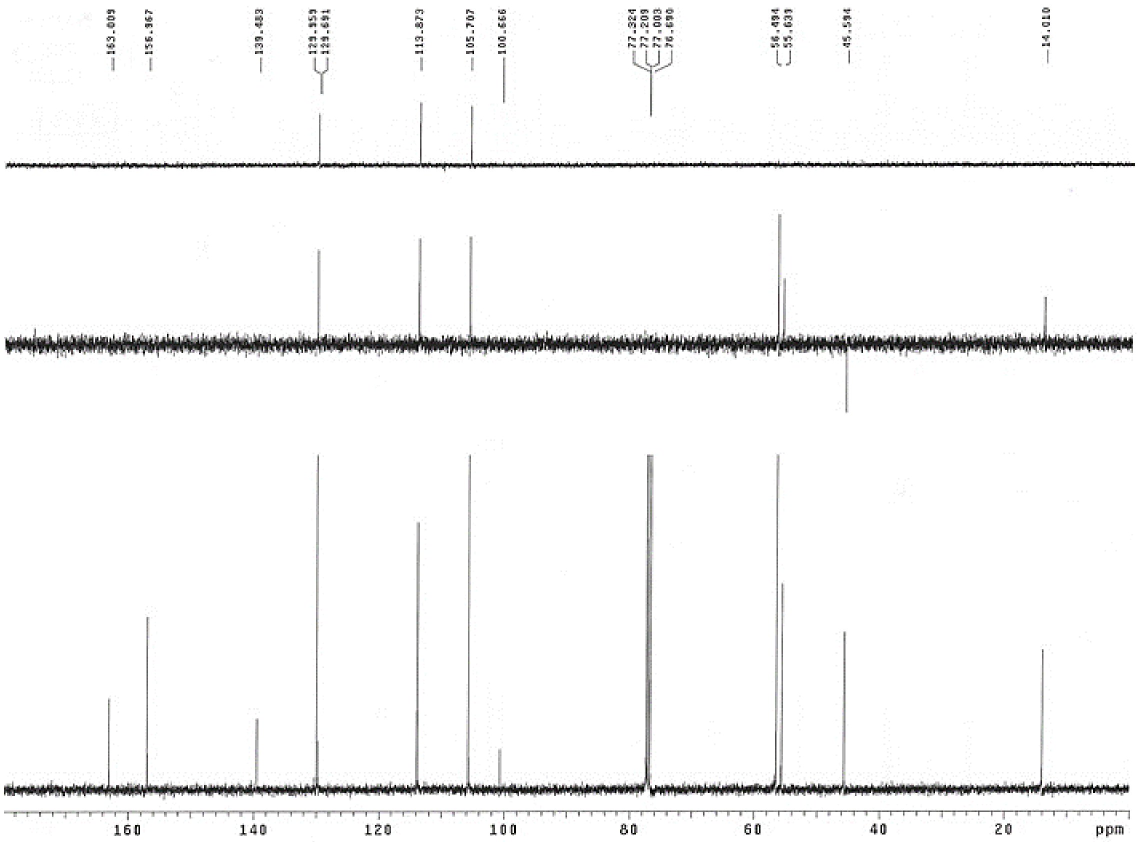

Fig. 10b: Compound **9a** ^{13}C NMR spectra.

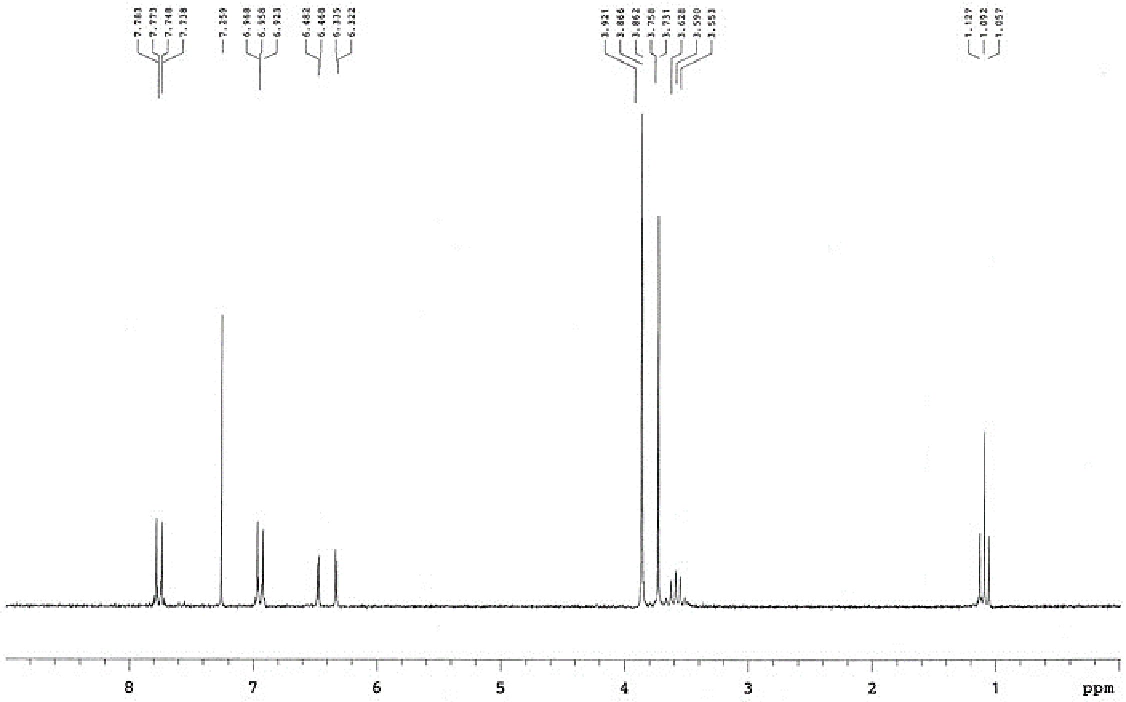

Fig. 11a: Compound **9b** ^{1}H NMR spectra.

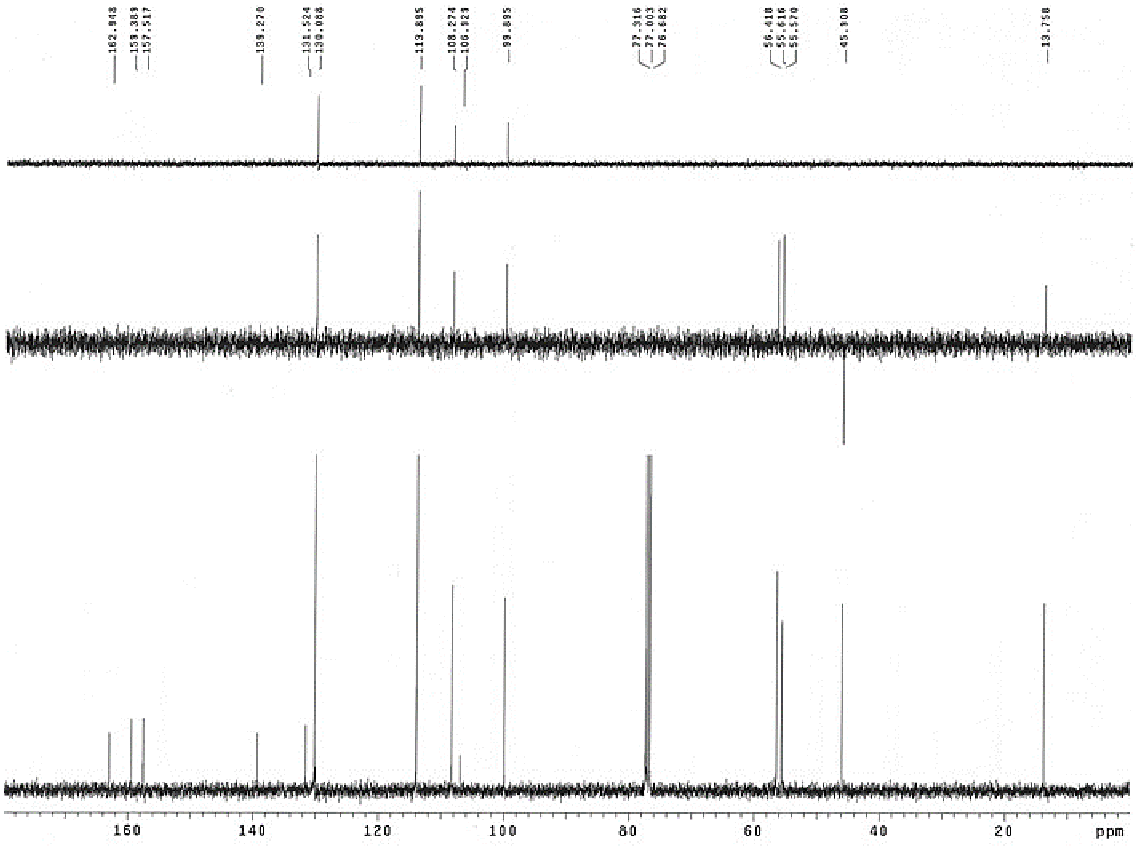

Fig. 11b: Compound **9b** ^{13}C NMR spectra.

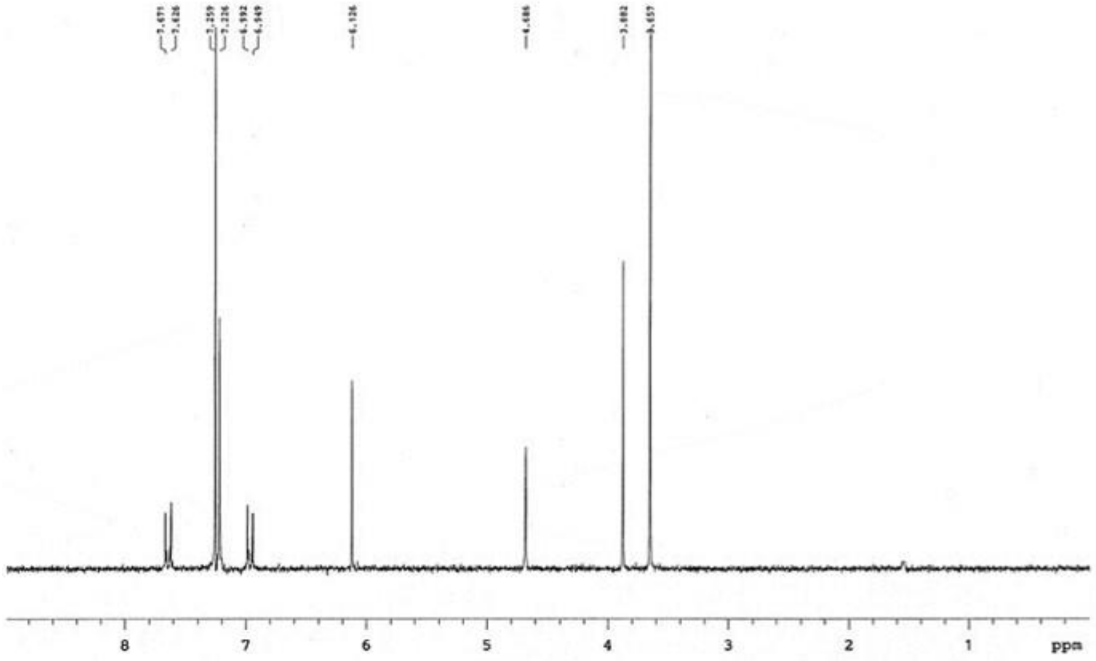

Fig. 12a: Compound **10a** ^{1}H NMR spectra.

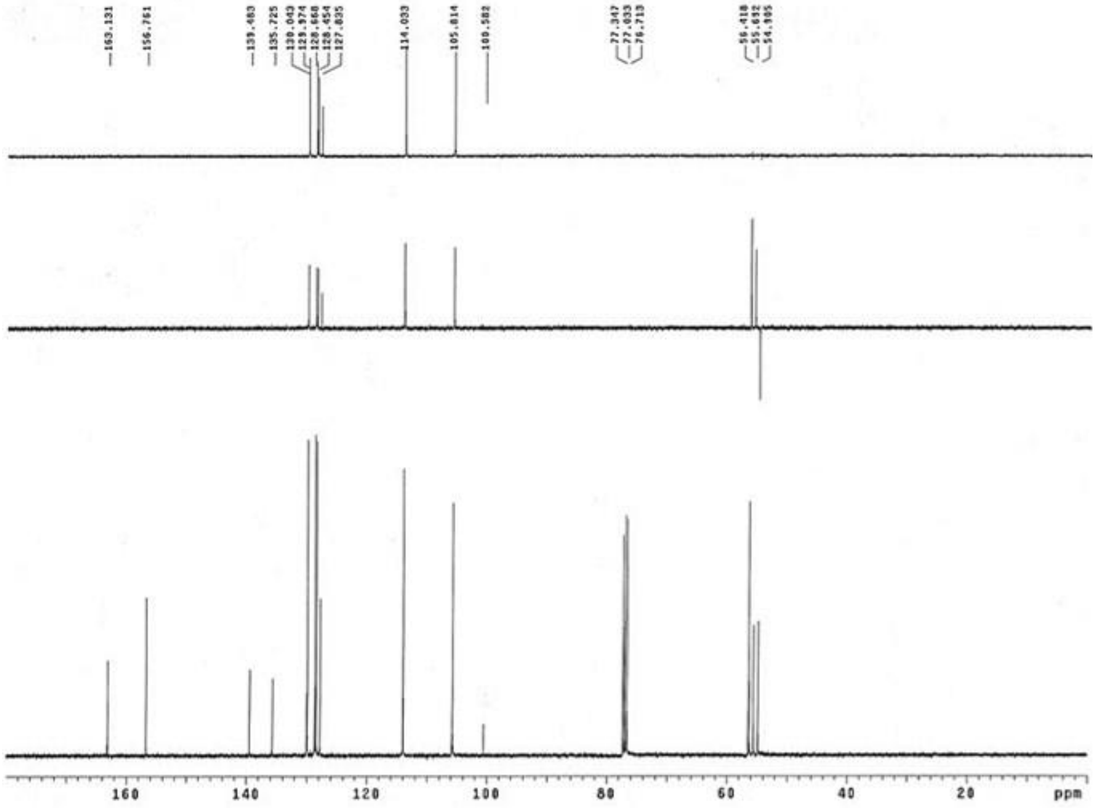

Fig. 12b: Compound **10a** ^{13}C NMR spectra.

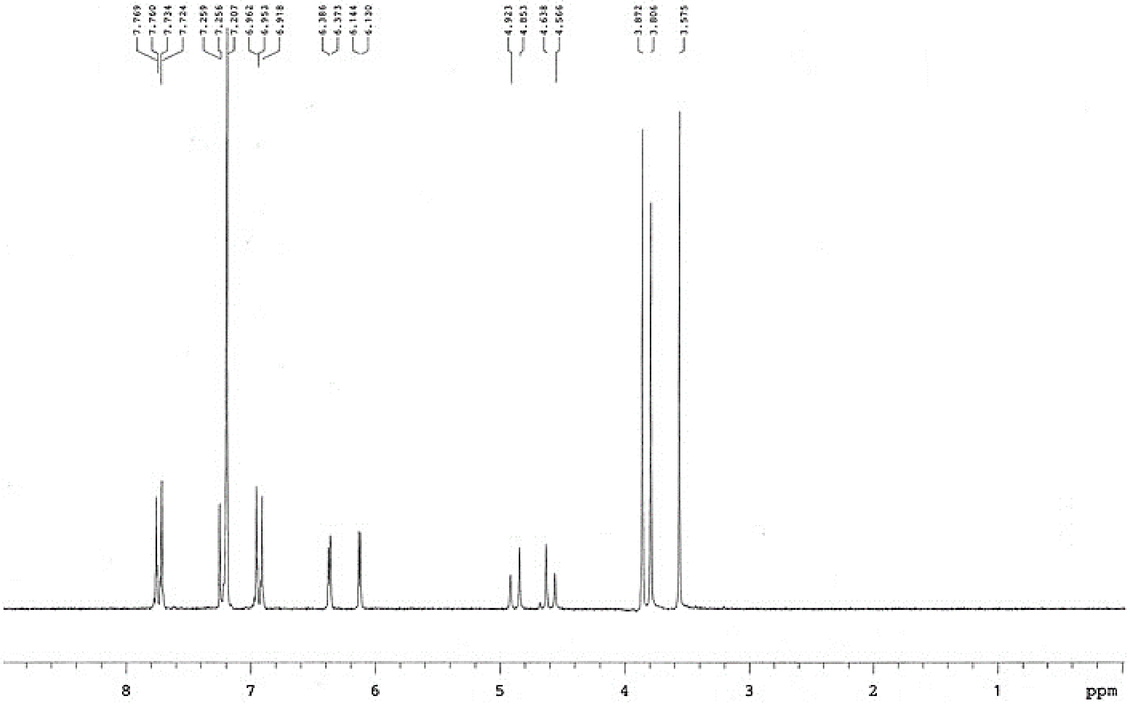

Fig. 13a: Compound **10b** ^{1}H NMR spectra.

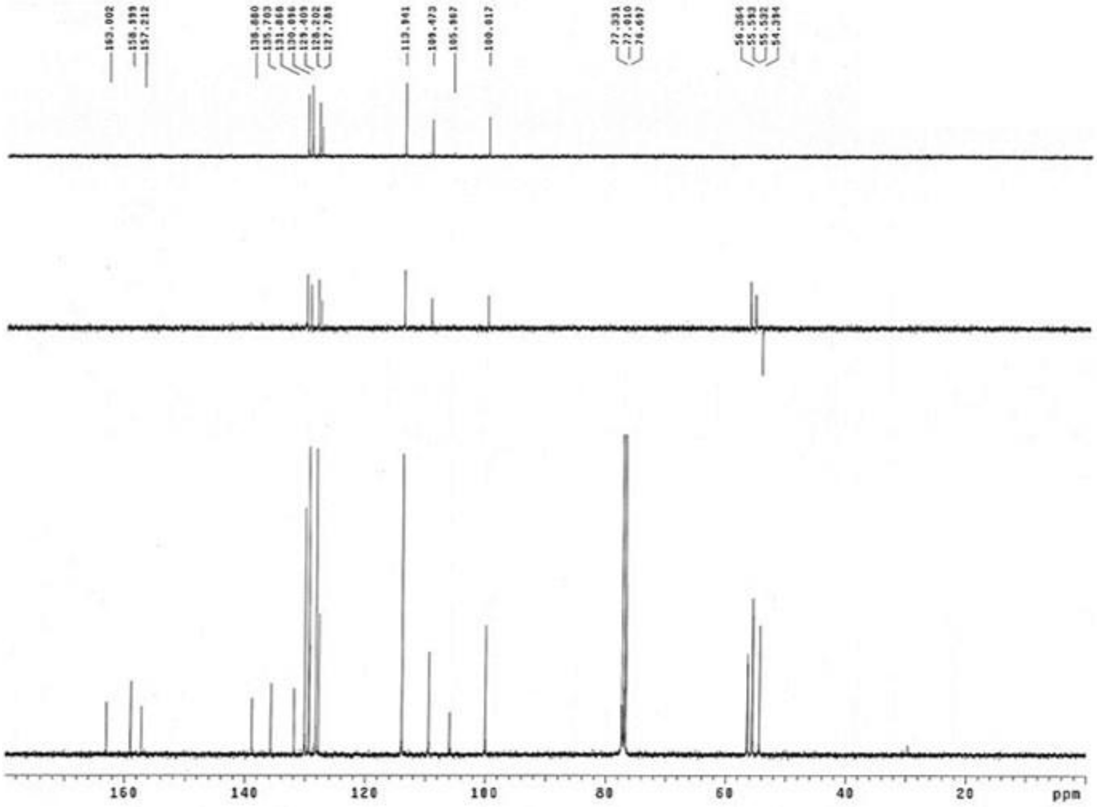

Fig. 13b: Compound **10b** ^{13}C NMR spectra.

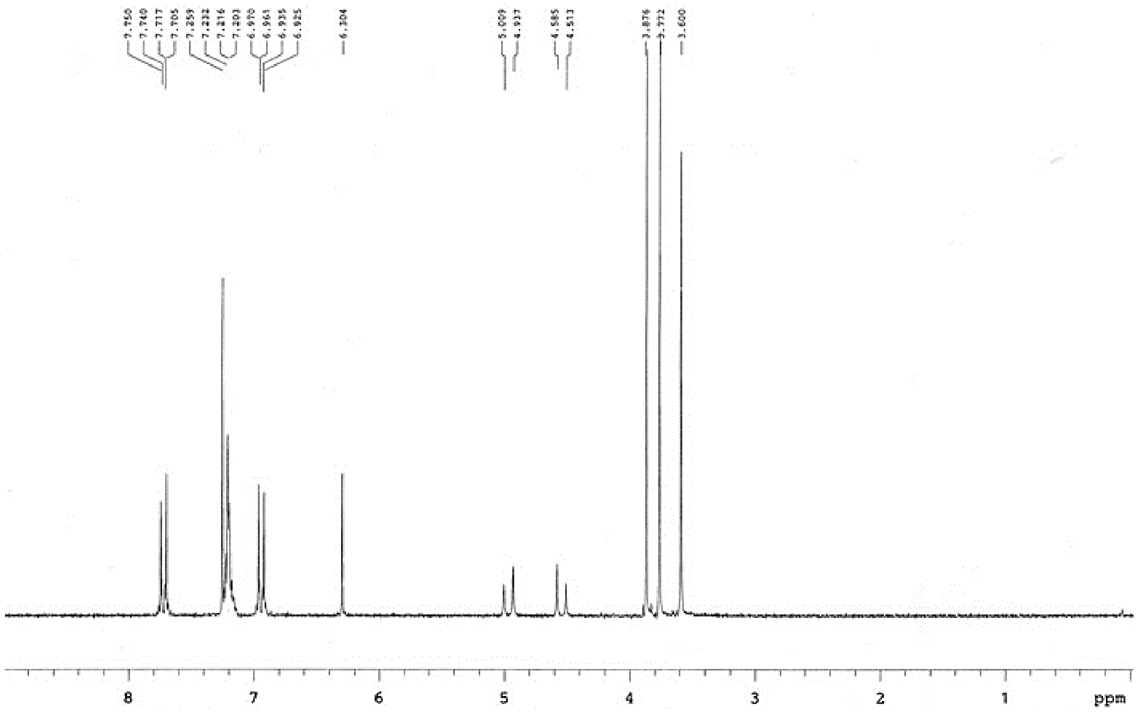

Fig. 14a: Compound **10c** ^{1}H NMR spectra.

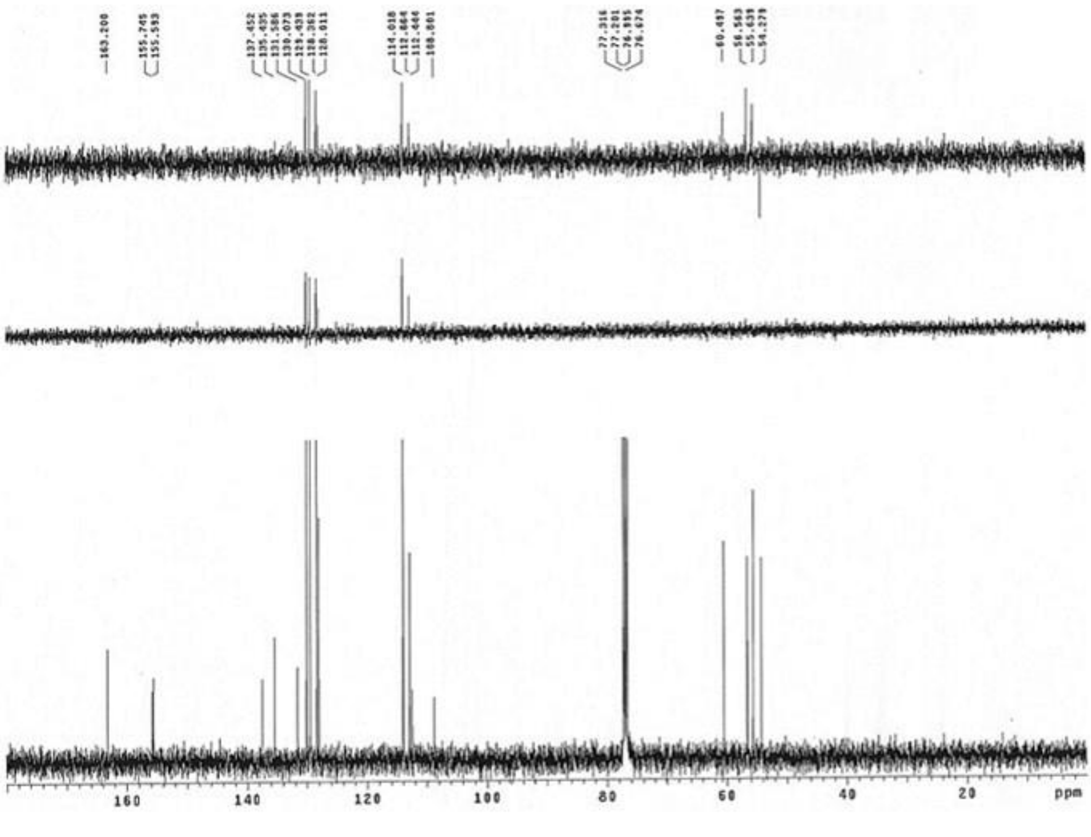

Fig. 14b: Compound **10c** ^{13}C NMR spectra.

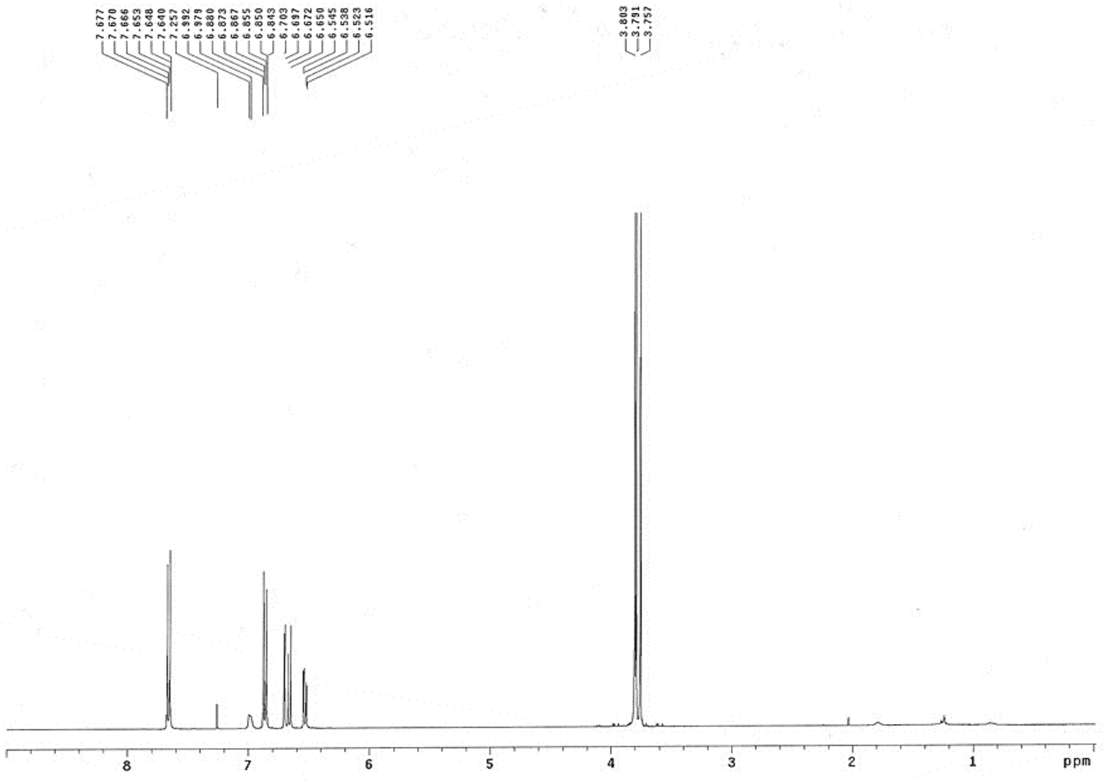

Fig. 15a: Compound **11** ^{1}H NMR spectra.

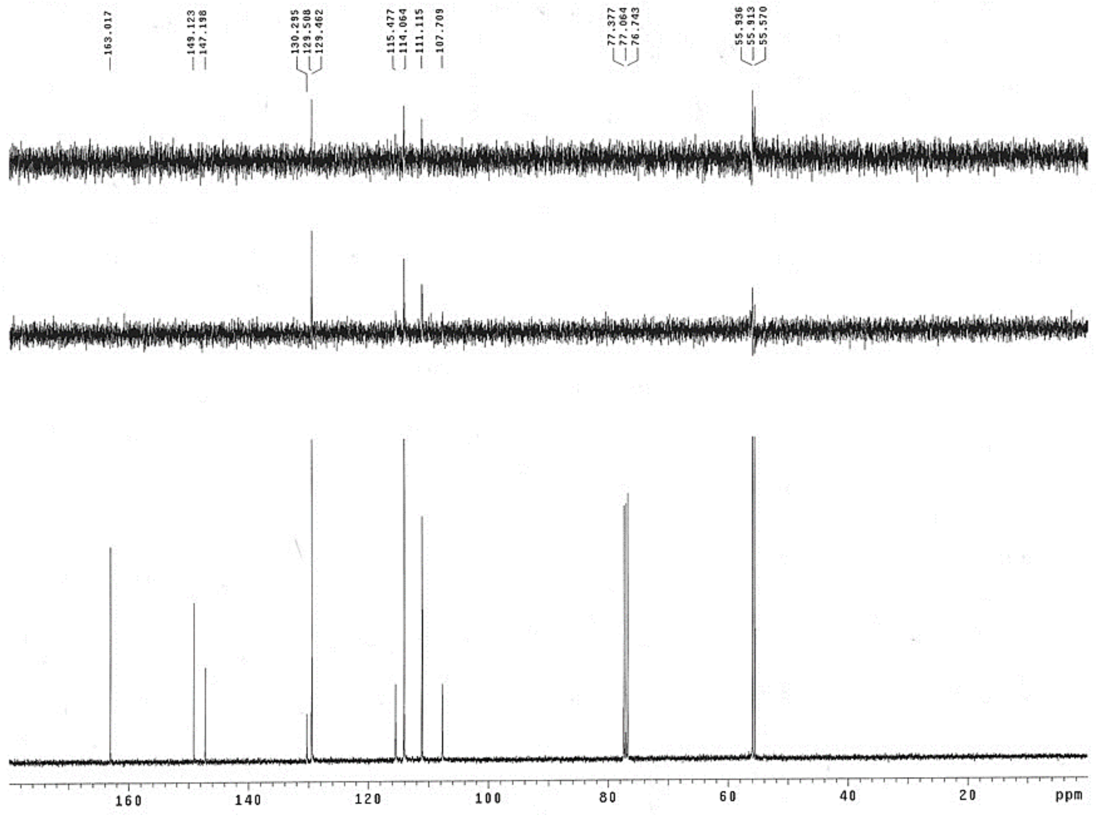

Fig. 15b: Compound **11** ^{13}C NMR spectra.

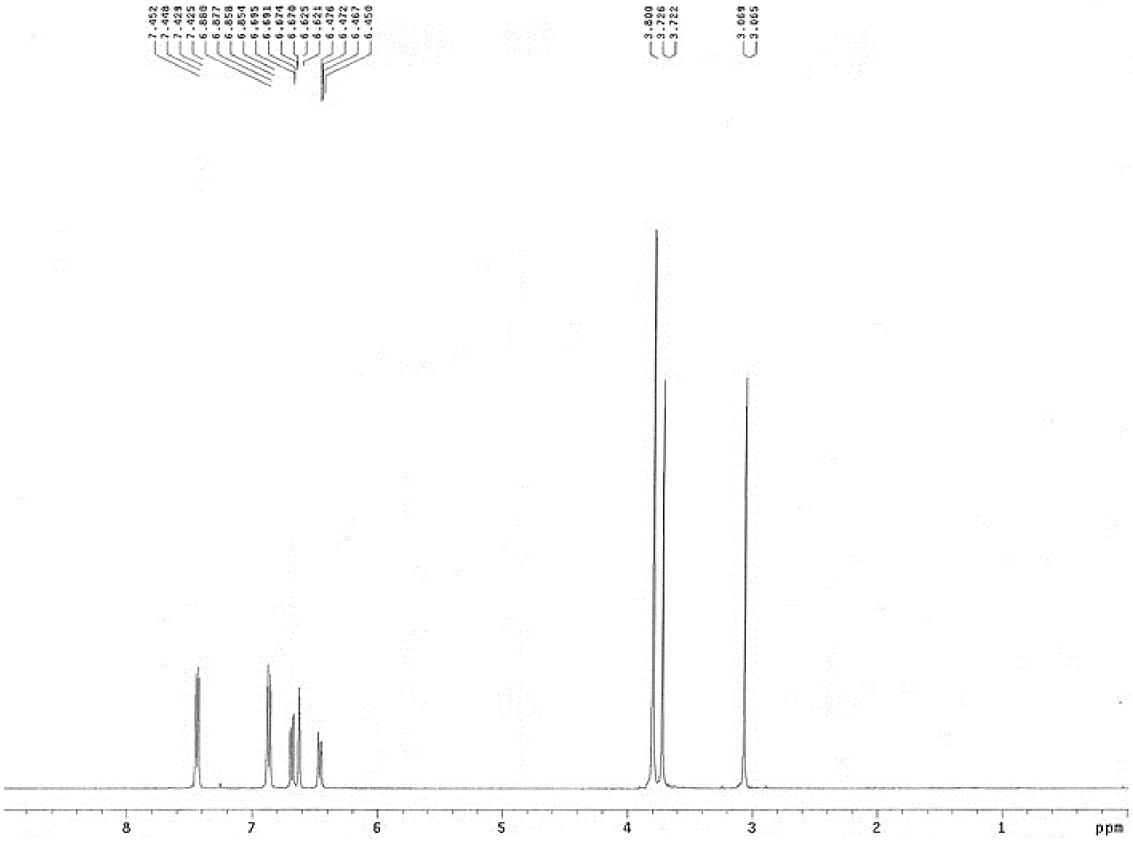

Fig. 16a: Compound **12** ^{1}H NMR spectra.

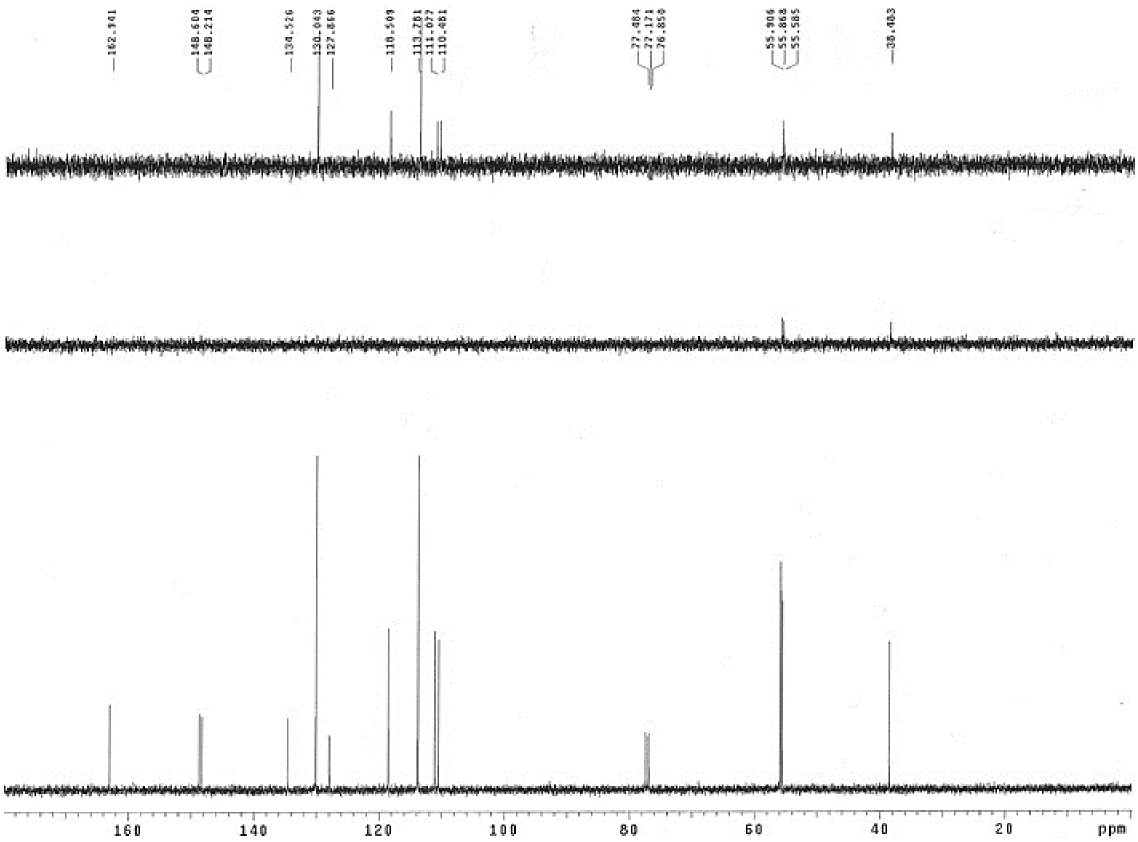

Fig. 16b: Compound **12** ^{13}C NMR spectra.

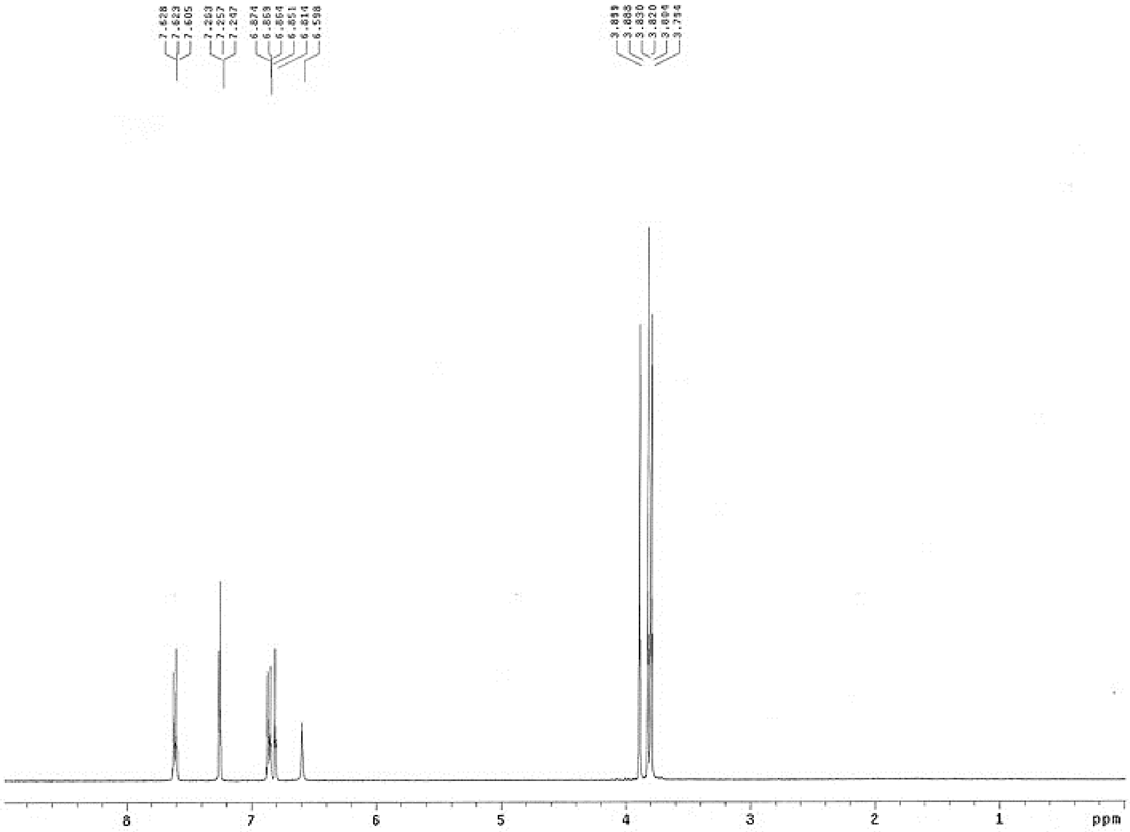

Fig. 17a: Compound **13** ^{1}H NMR spectra.

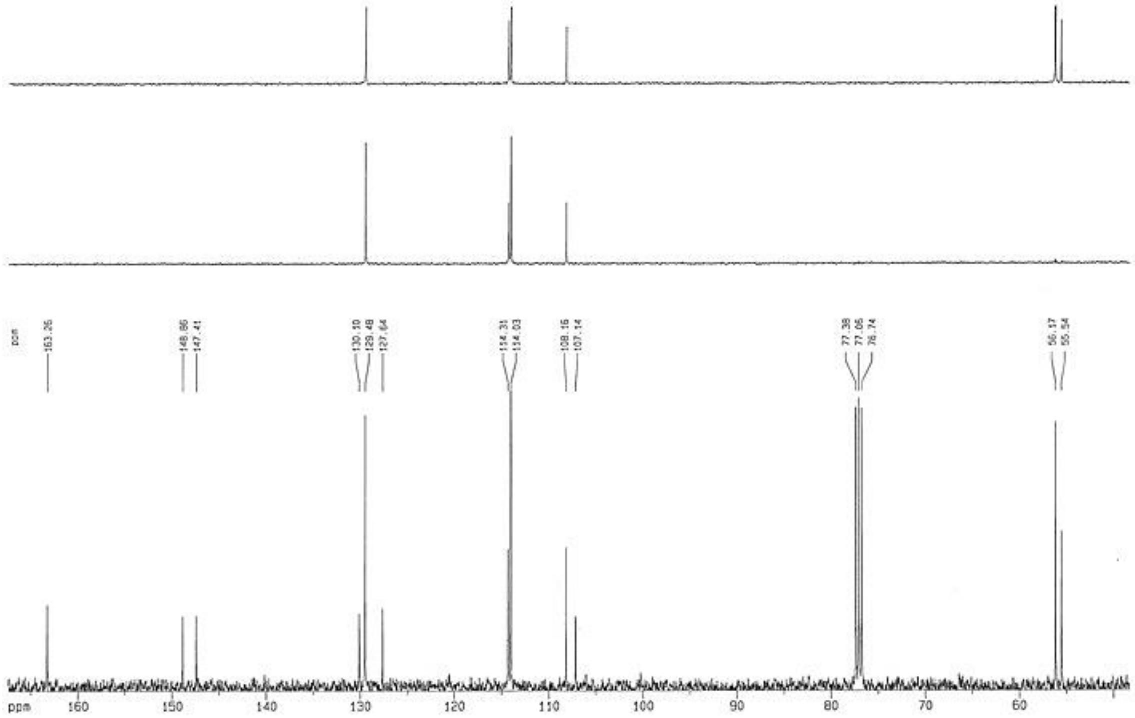

Fig. 17b: Compound **13** ^{13}C NMR spectra.

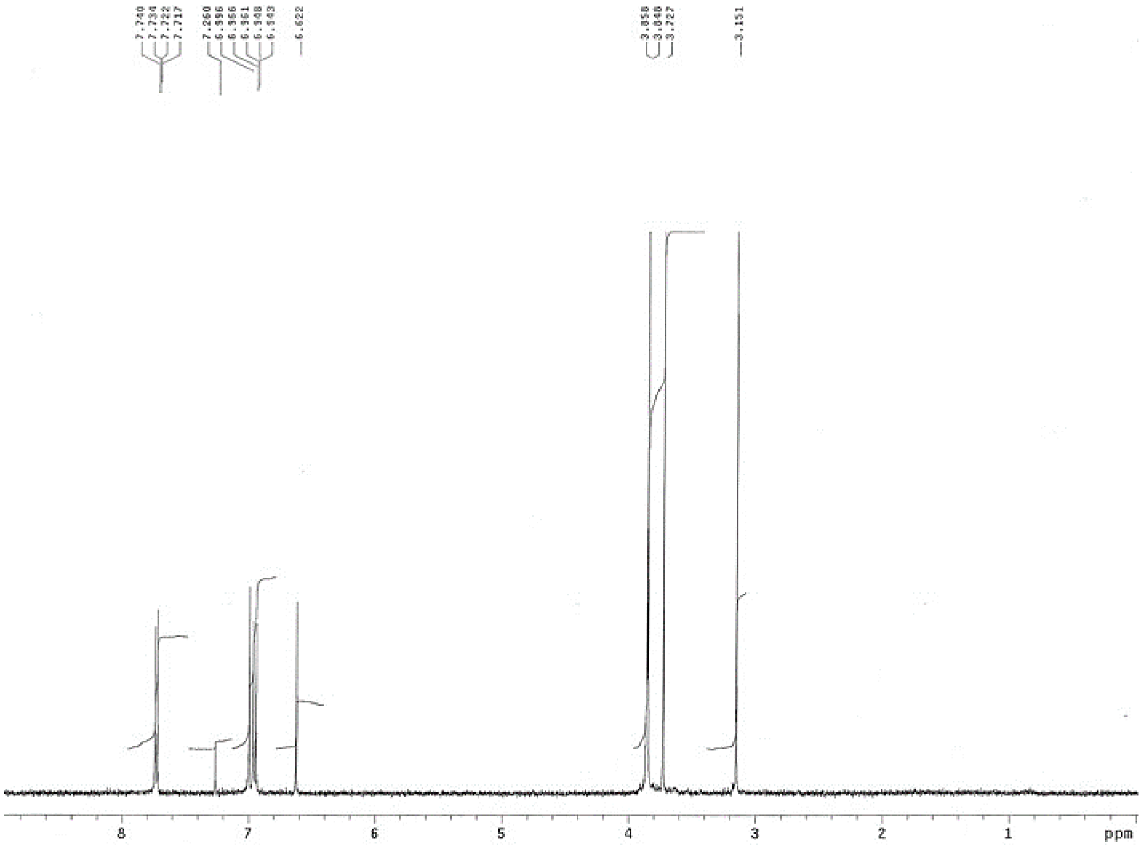

Fig. 18a: Compound **14** ^{1}H NMR spectra.

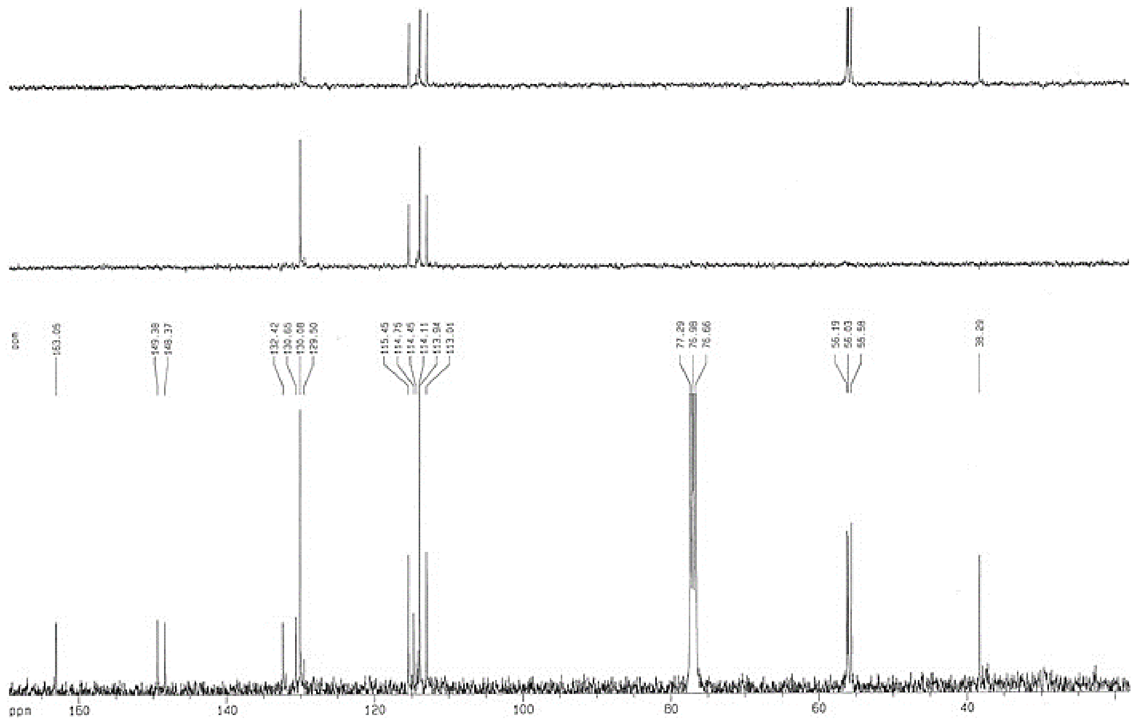

Fig. 18b: Compound **14** ^{13}C NMR spectra.

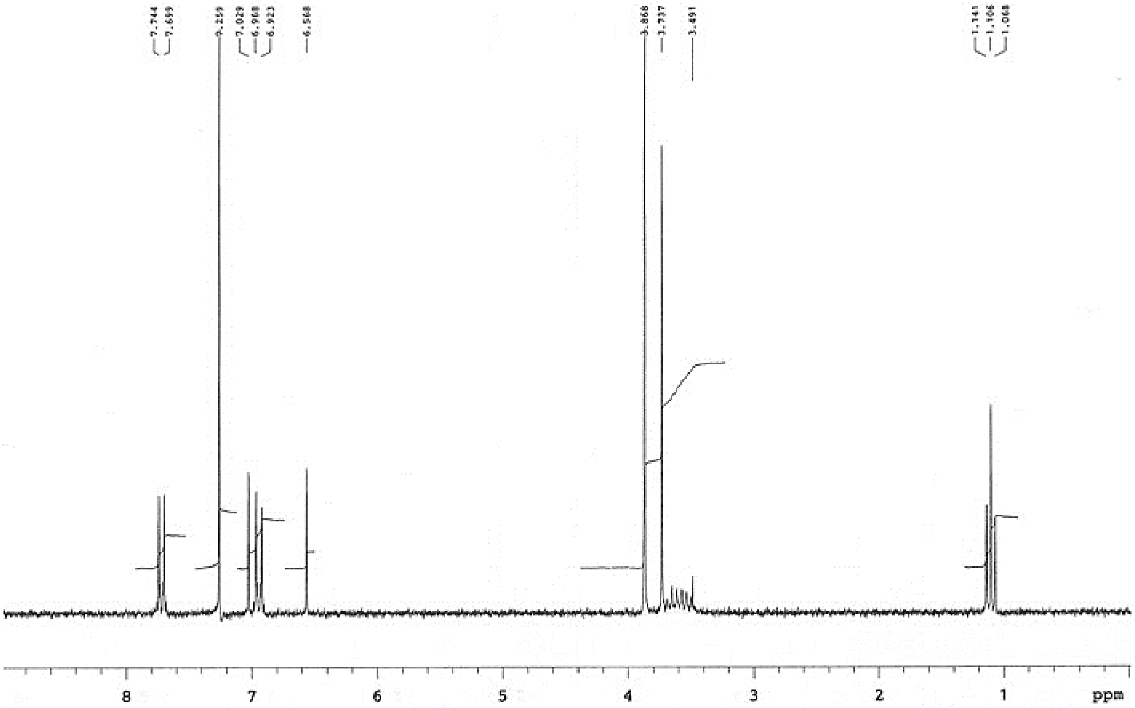

Fig. 19a: Compound **15** ^{1}H NMR spectra.

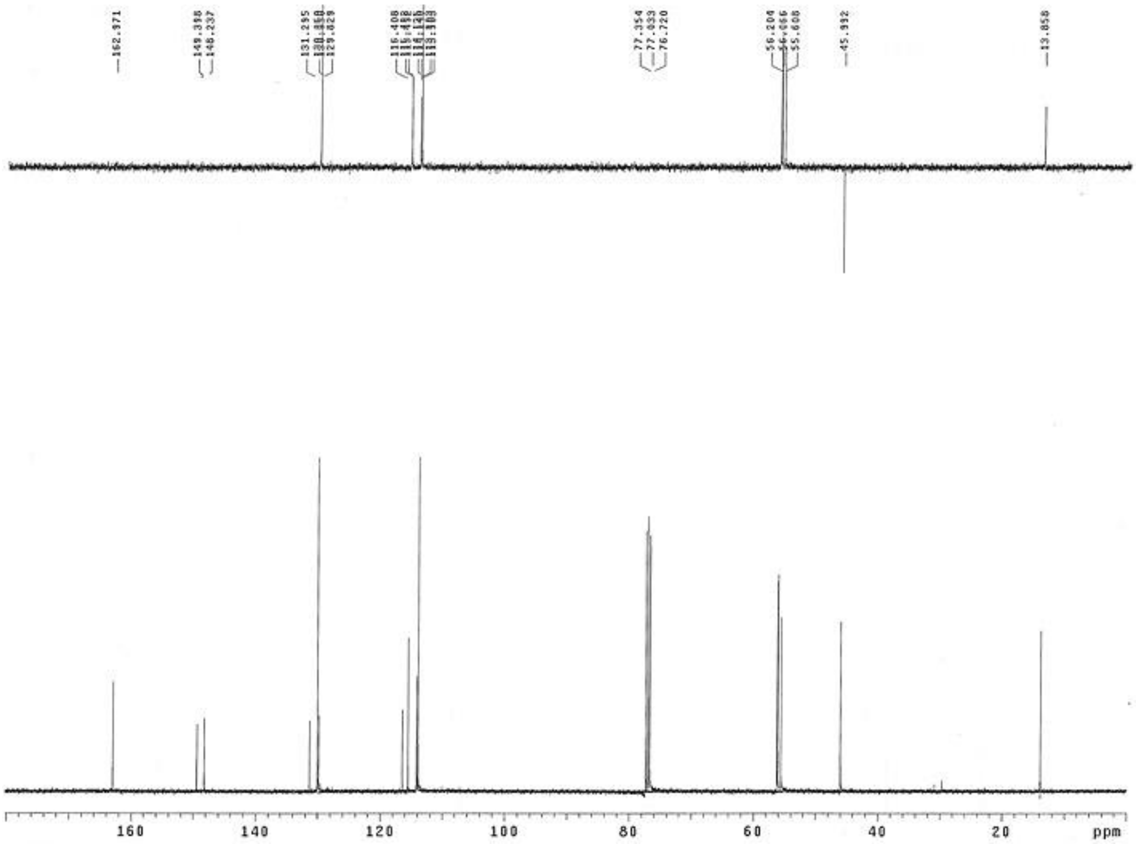

Fig. 19b: Compound **15** ^{13}C NMR spectra.

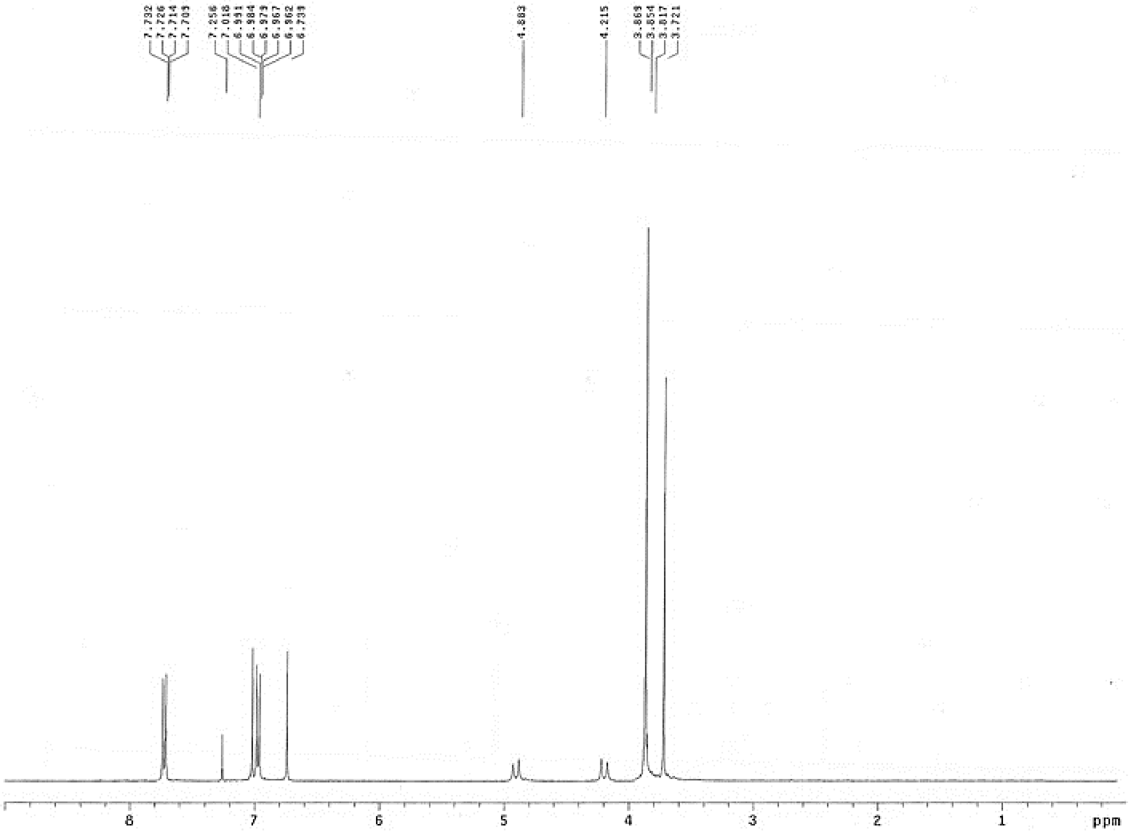

Fig. 20a: Compound **16** ^{1}H NMR spectra.

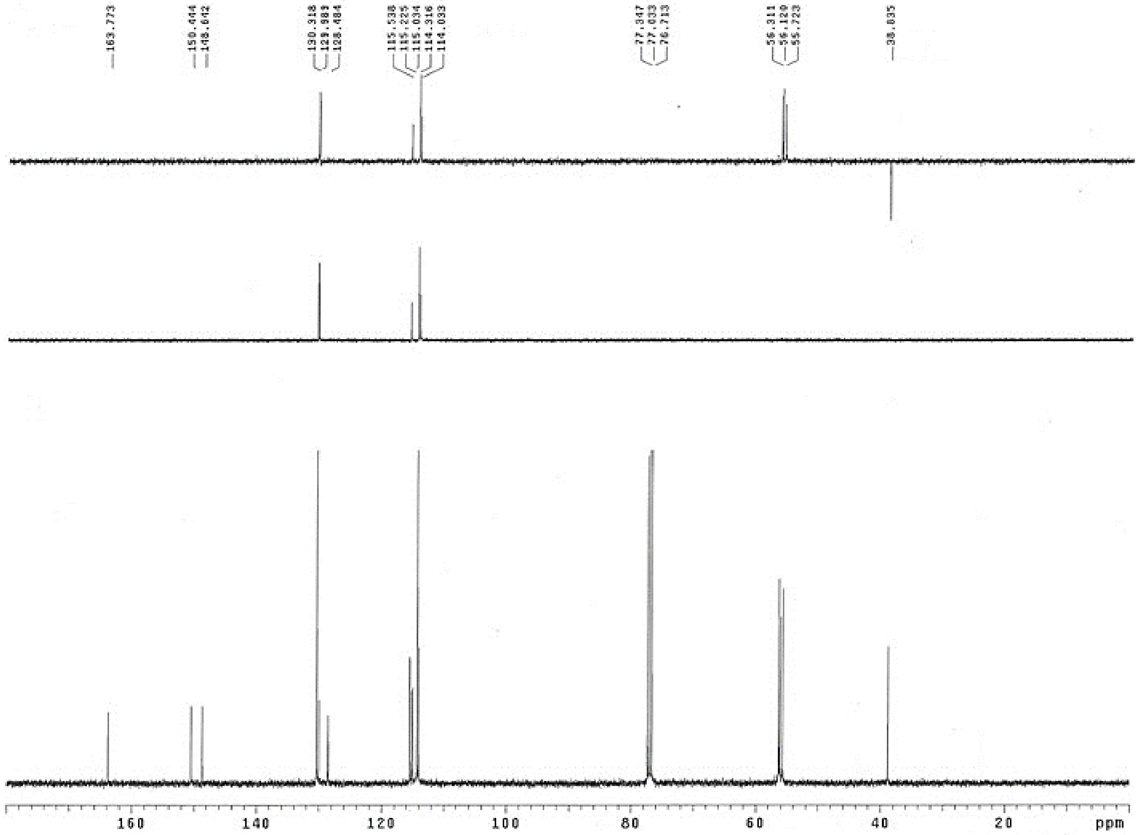

Fig. 20b: Compound **16** ^{13}C NMR spectra.

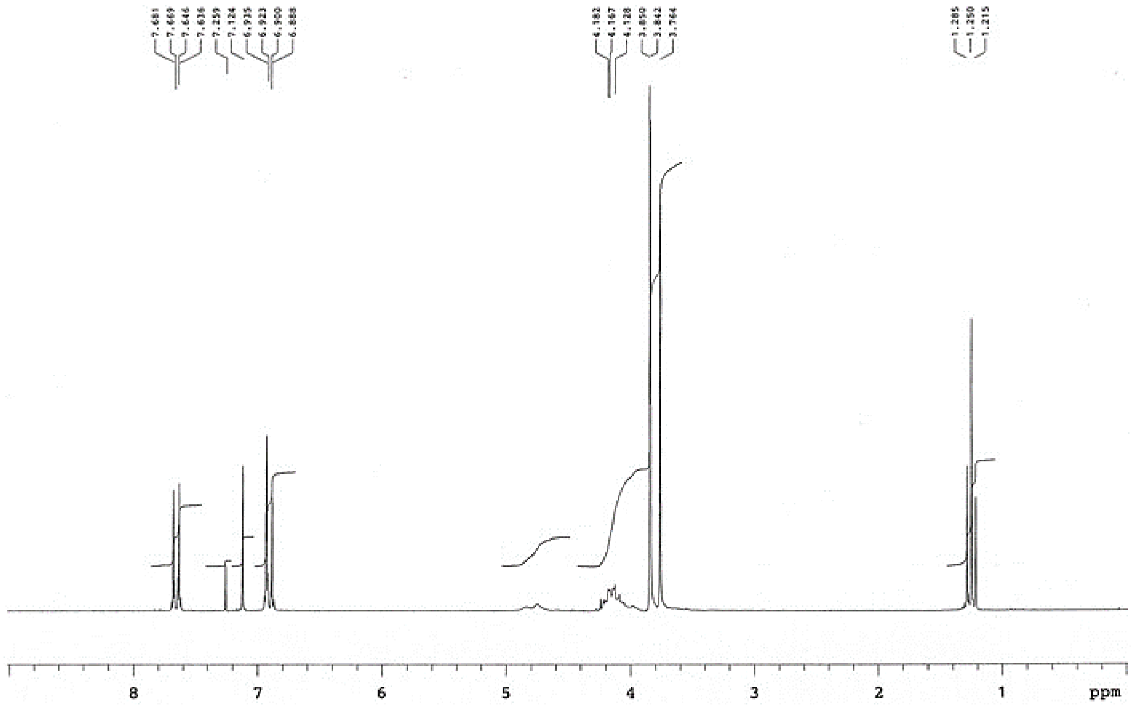

Fig. 21a: Compound **17** ^{1}H NMR spectra.

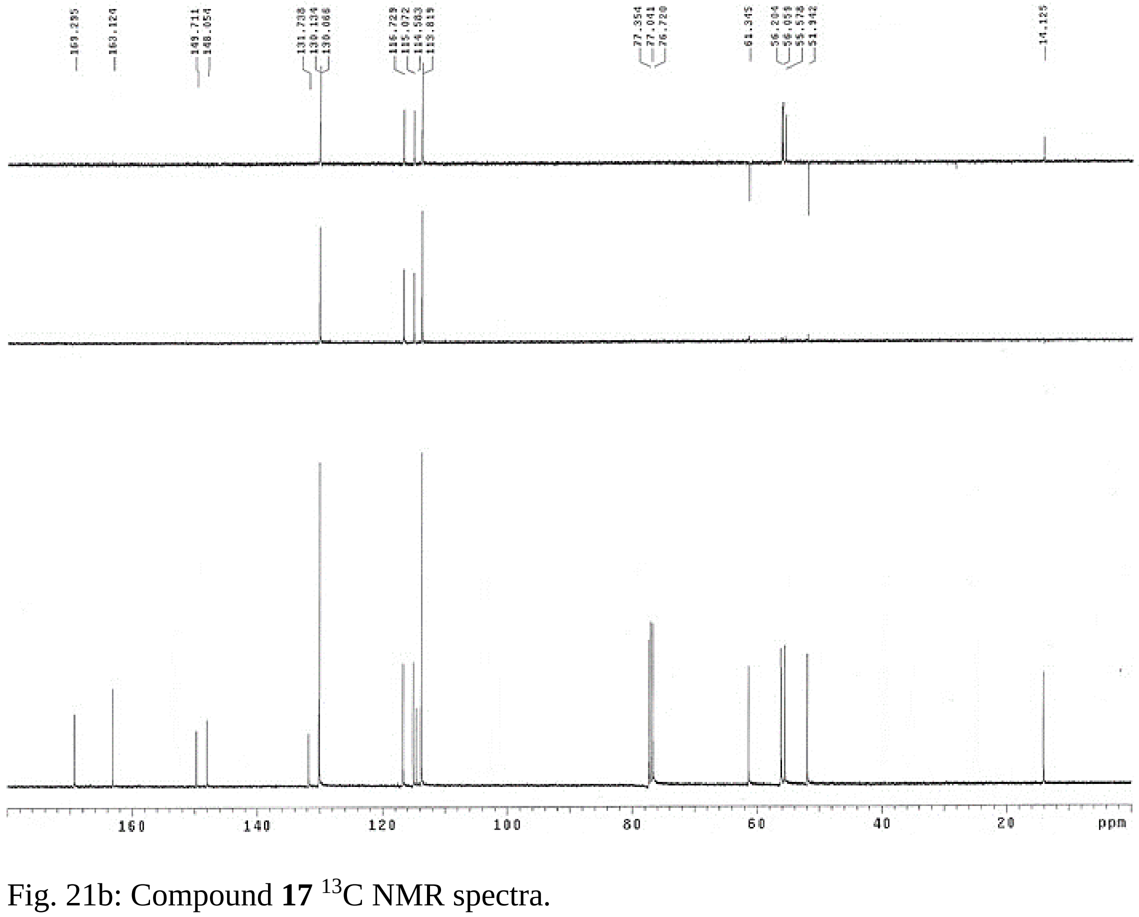

Fig. 21b: Compound **17** ^{13}C NMR spectra.

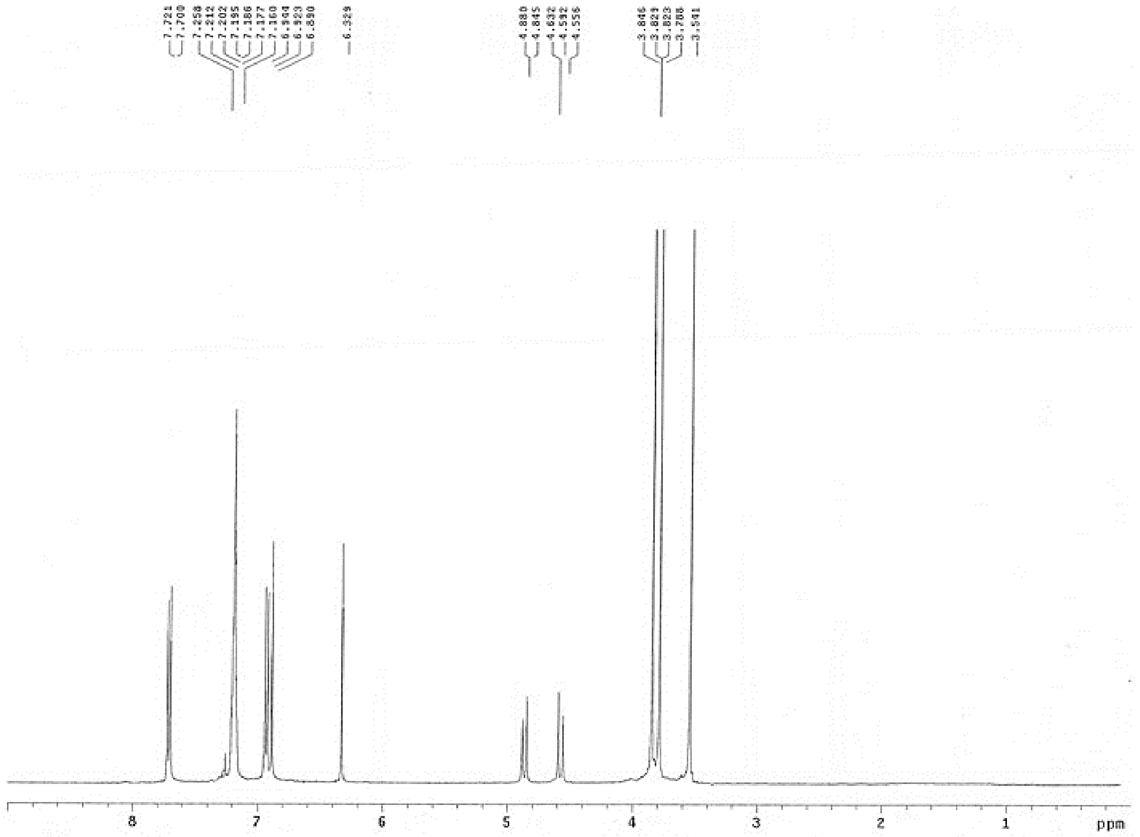

Fig. 22a: Compound **18** ^{1}H NMR spectra.

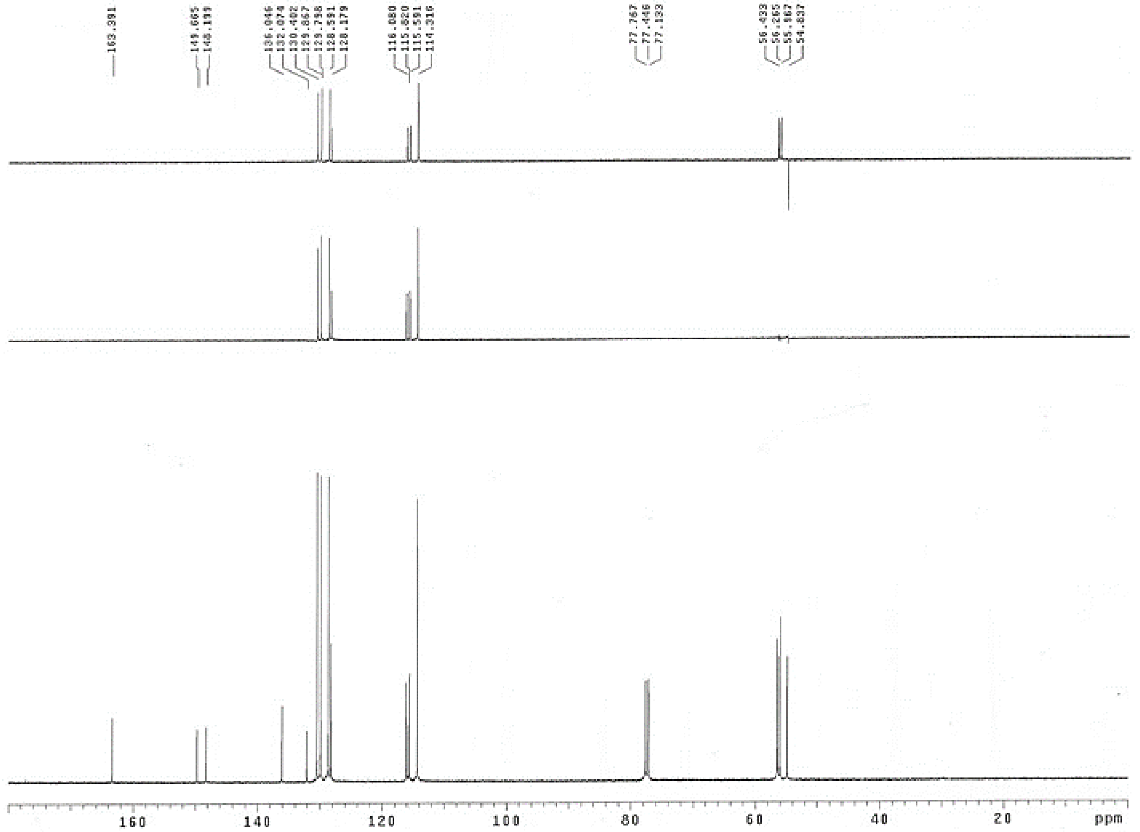

Fig. 22b: Compound **18** ^{13}C NMR spectra.

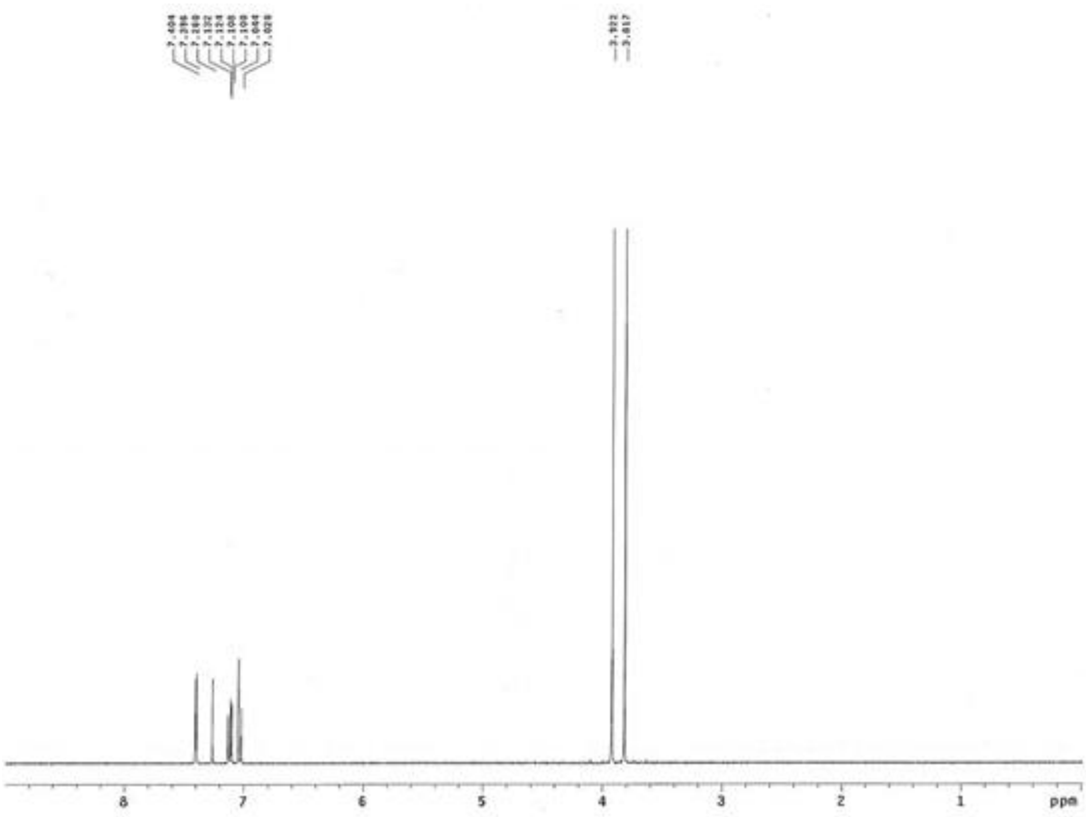

Fig.23a: Compound **19** ^{1}H NMR spectra.

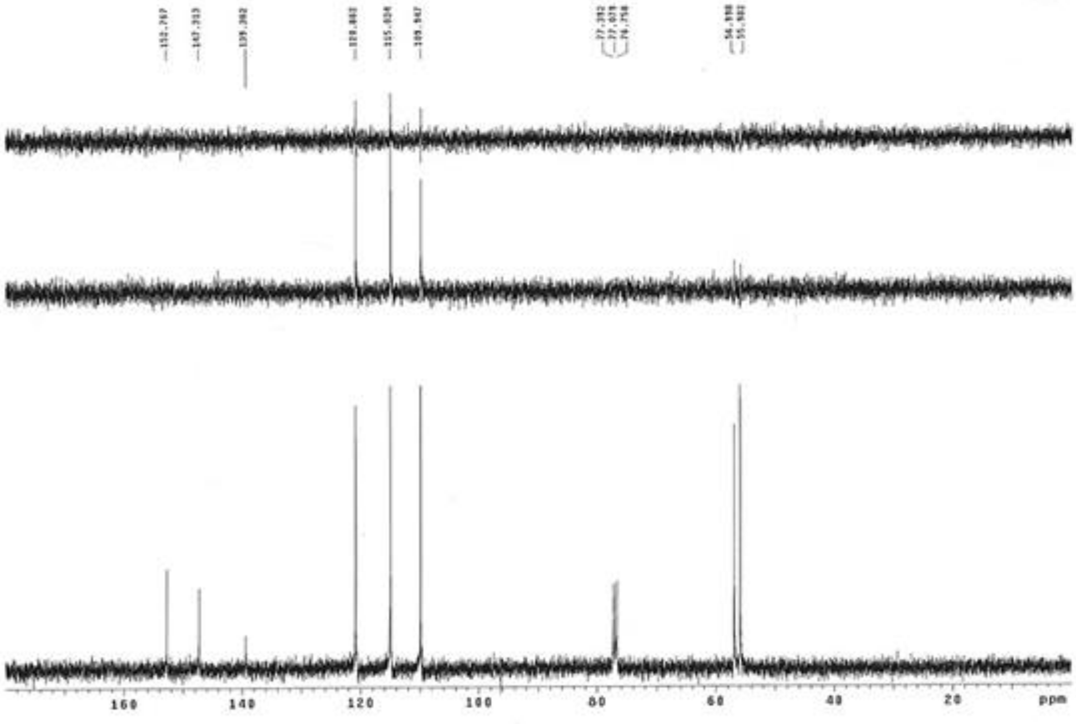

Fig. 23b: Compound **19** ^{13}C NMR spectra.

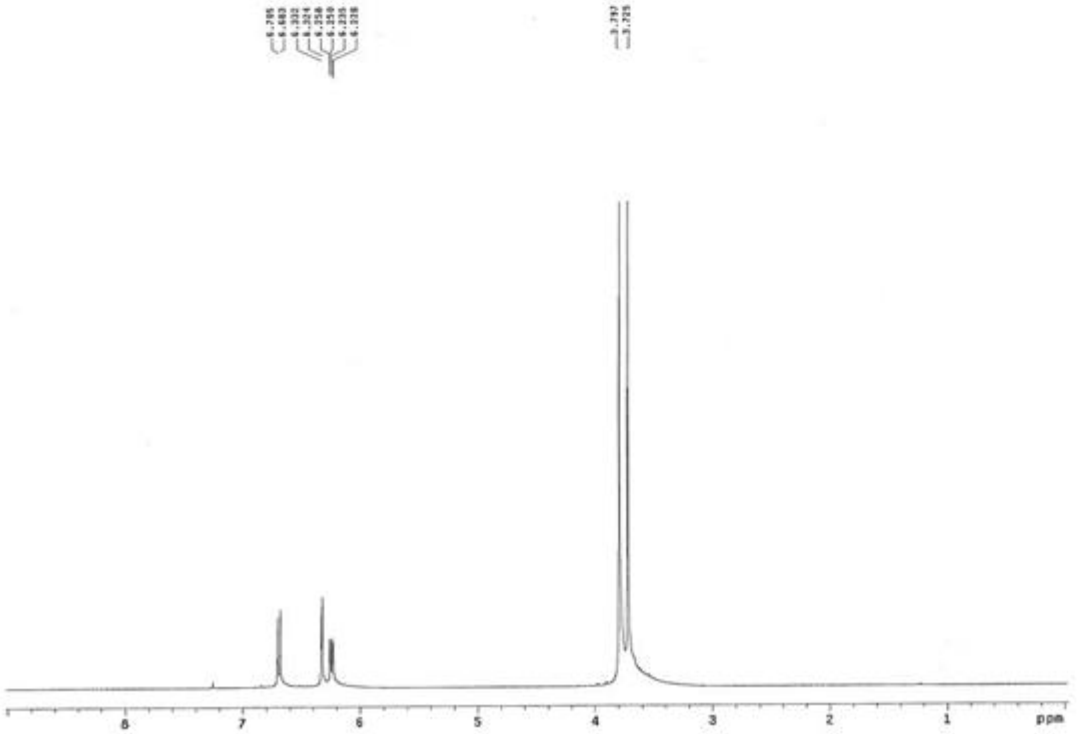

Fig. 24a: Compound **20** ^{1}H NMR spectra.

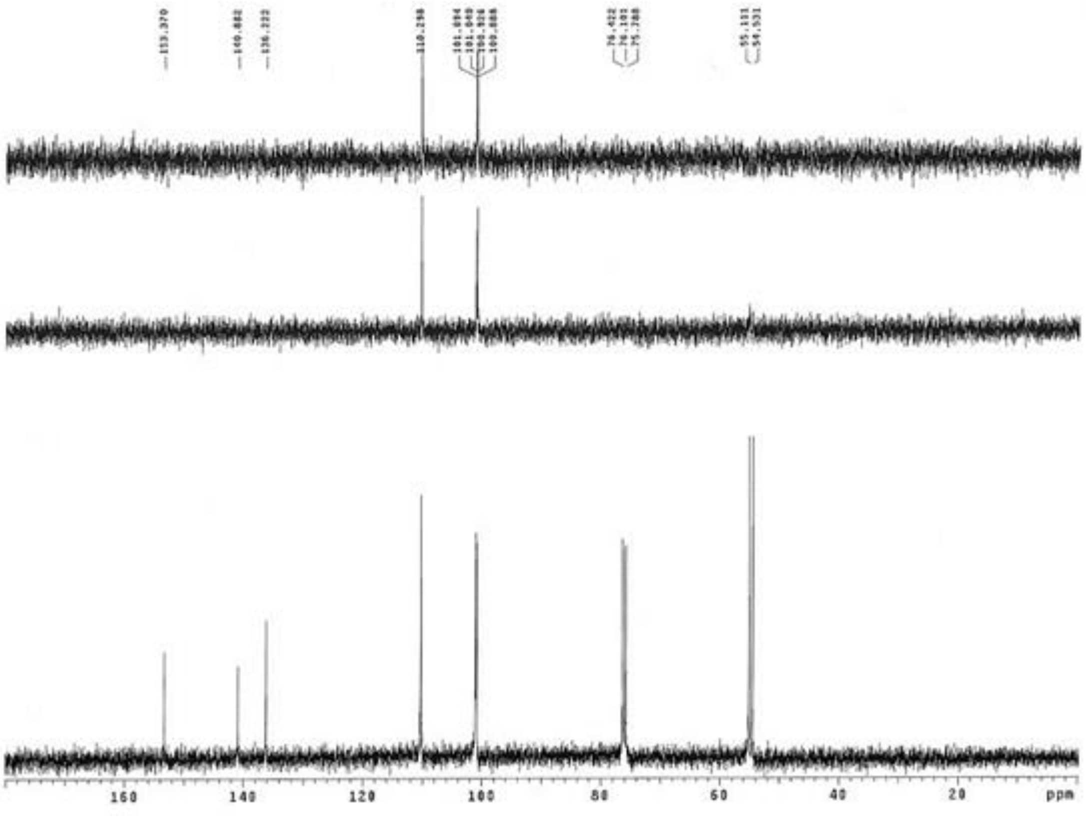

Fig. 24b: Compound **20** ^{13}C NMR spectra.

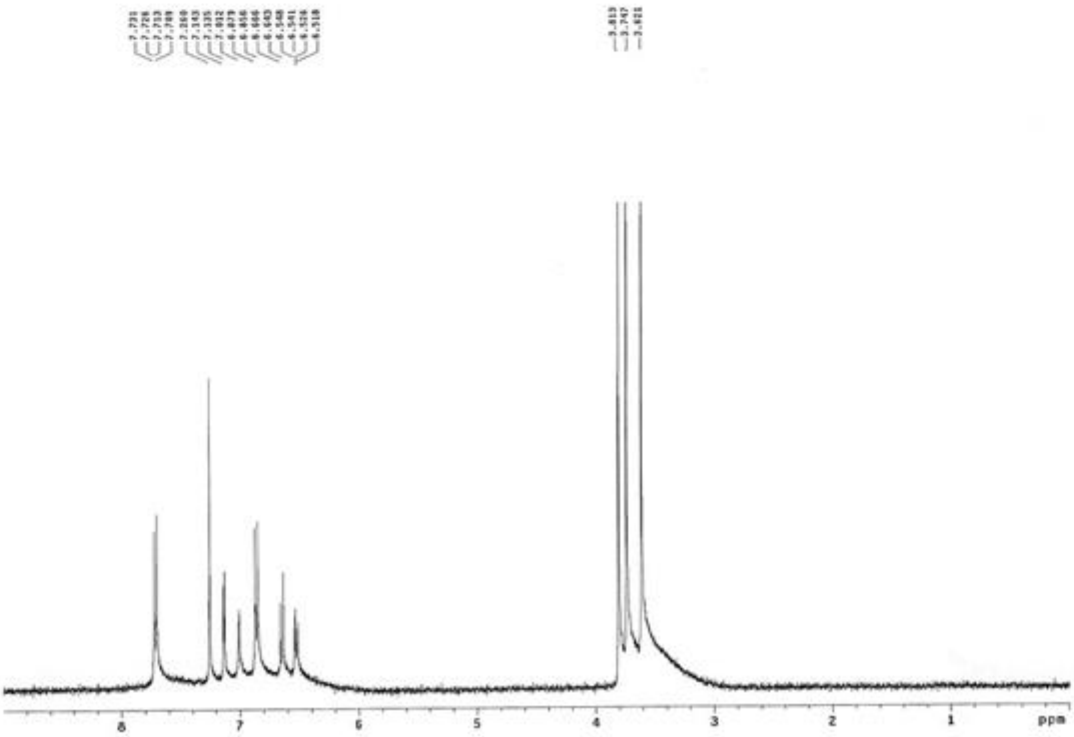

Fig. 25a: Compound **21** ^{1}H NMR spectra.

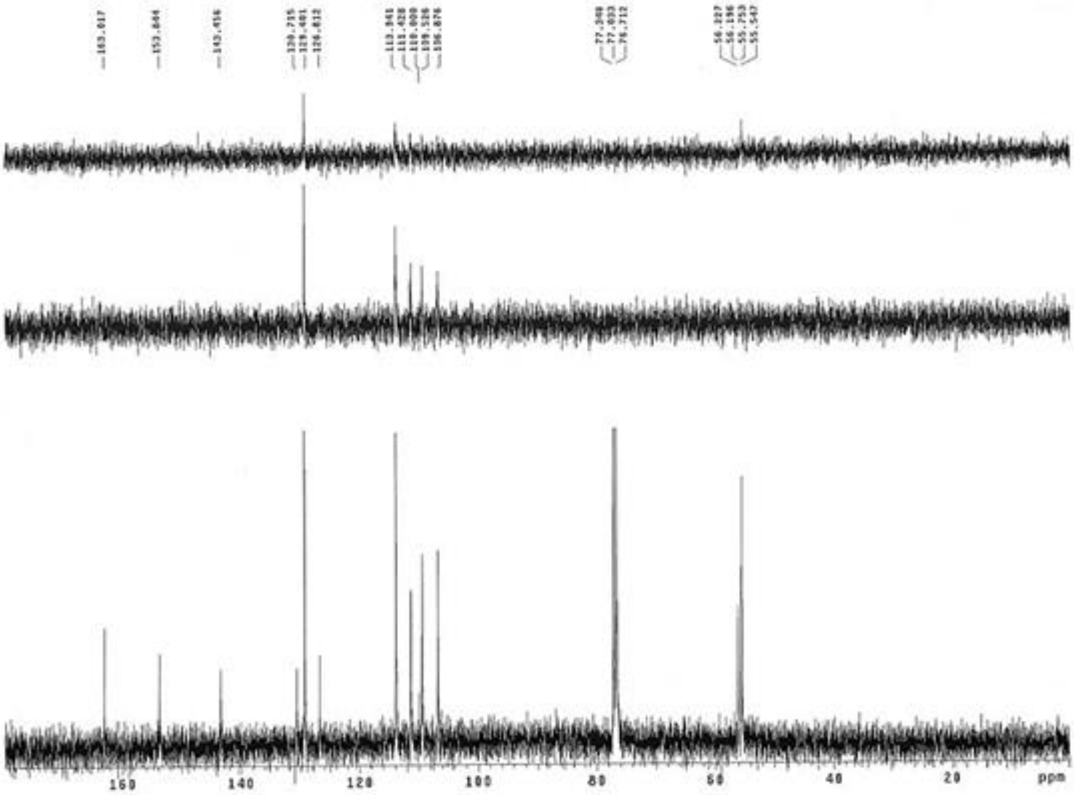

Fig. 25b: Compound **21** ^{13}C NMR spectra.

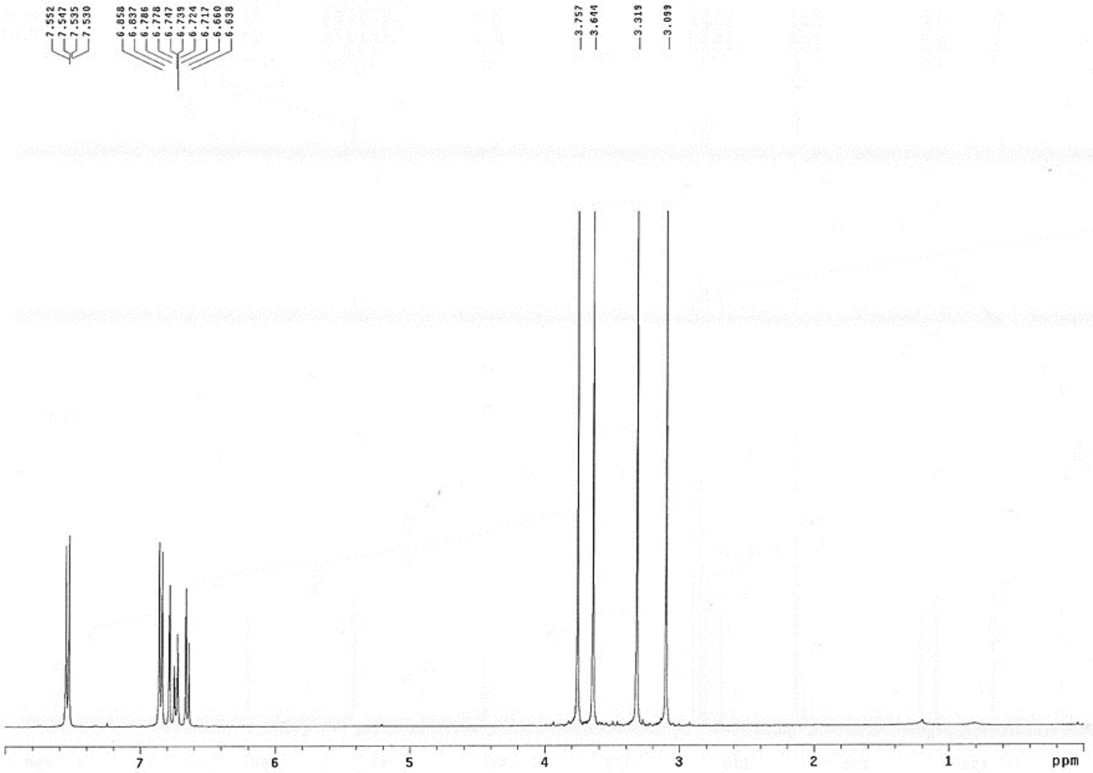

Fig. 26a: Compound **22** ^{1}H NMR spectra.

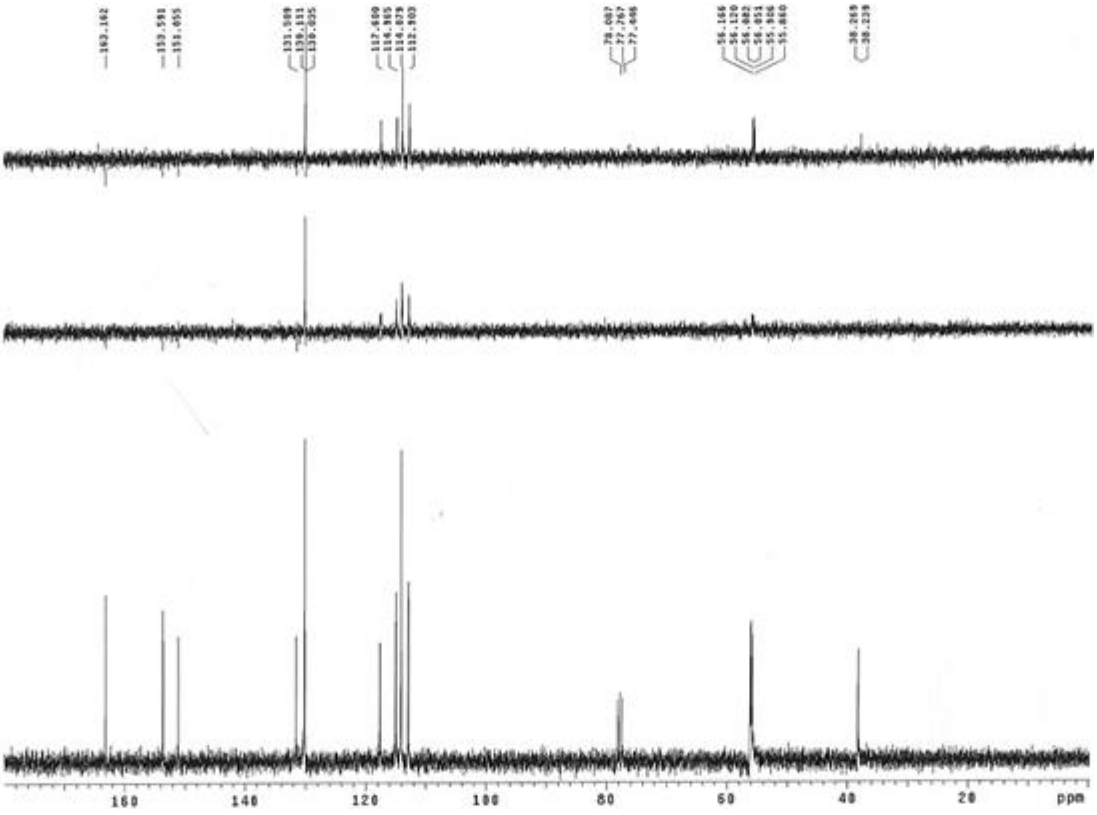

Fig. 26b: Compound **22** ^{13}C NMR spectra.

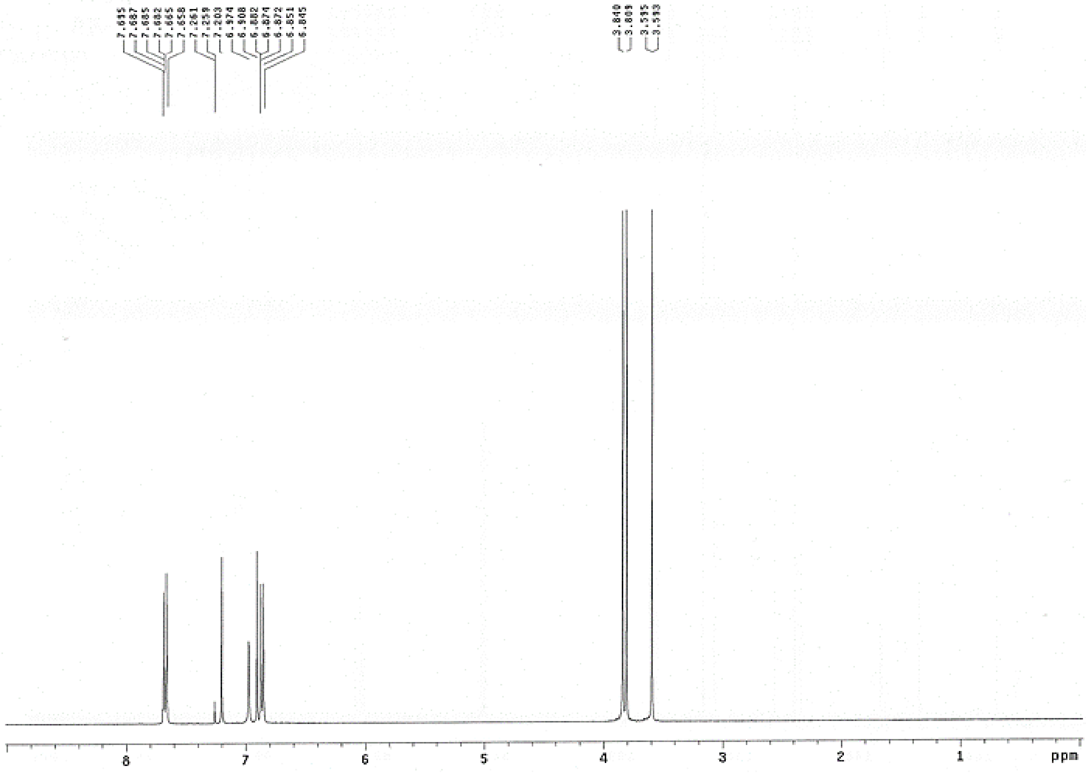

Fig. 27a: Compound **23** ^{1}H NMR spectra.

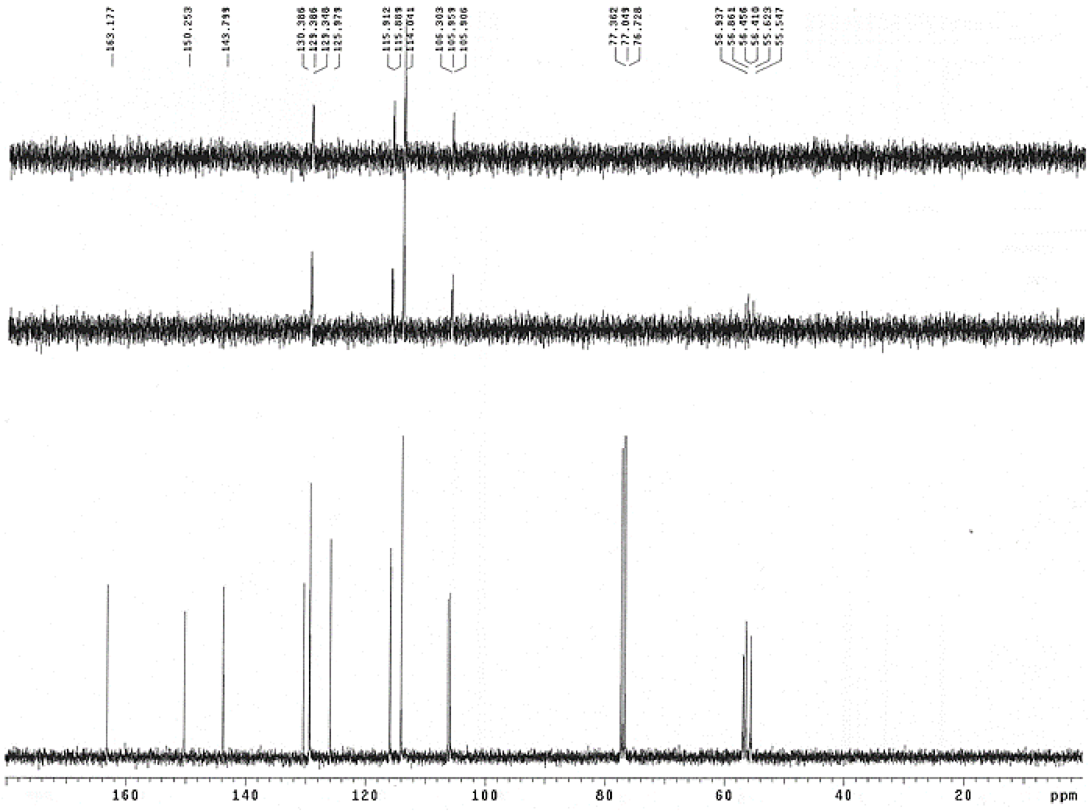

Fig. 27b: Compound **23** ^{13}C NMR spectra.

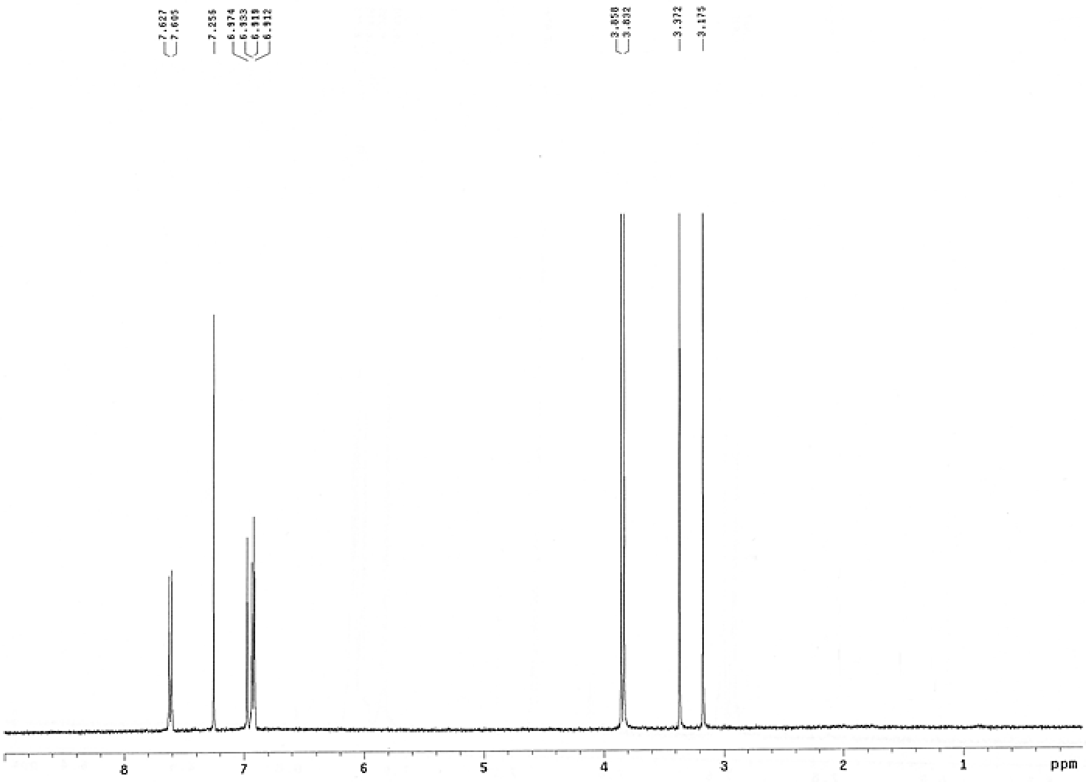

Fig. 28a: Compound **24** ^{1}H NMR spectra.

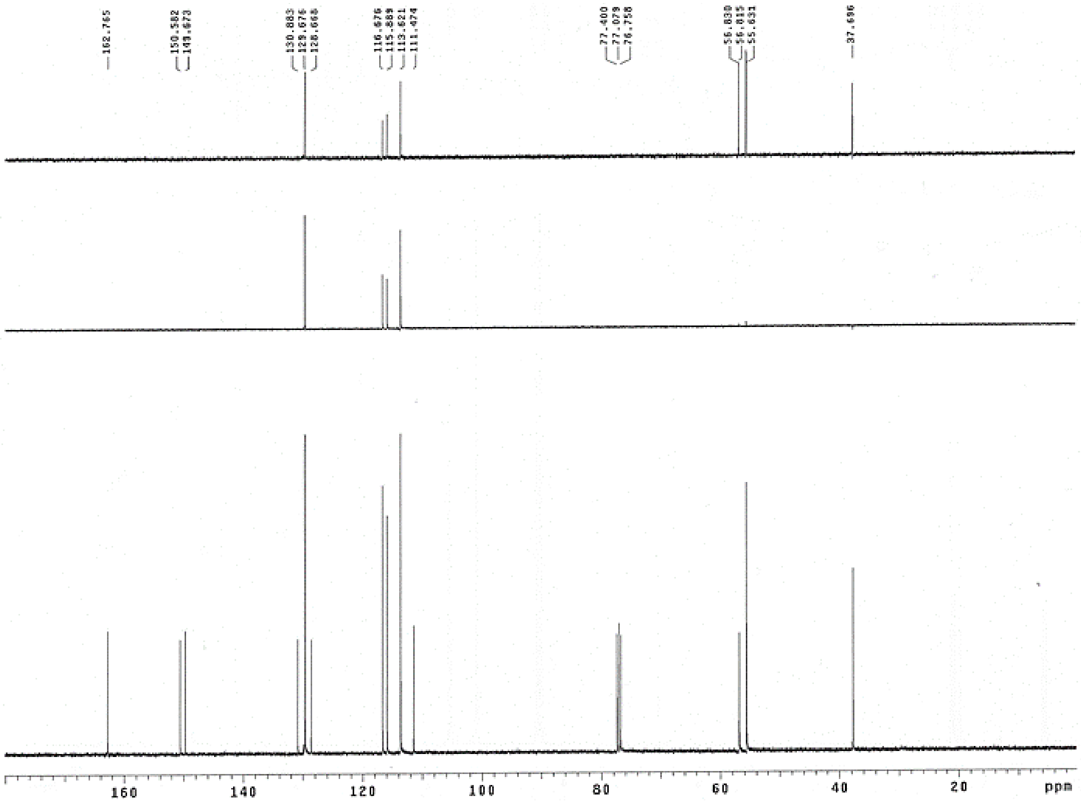

Fig. 28b: Compound **24** ^{13}C NMR spectra.

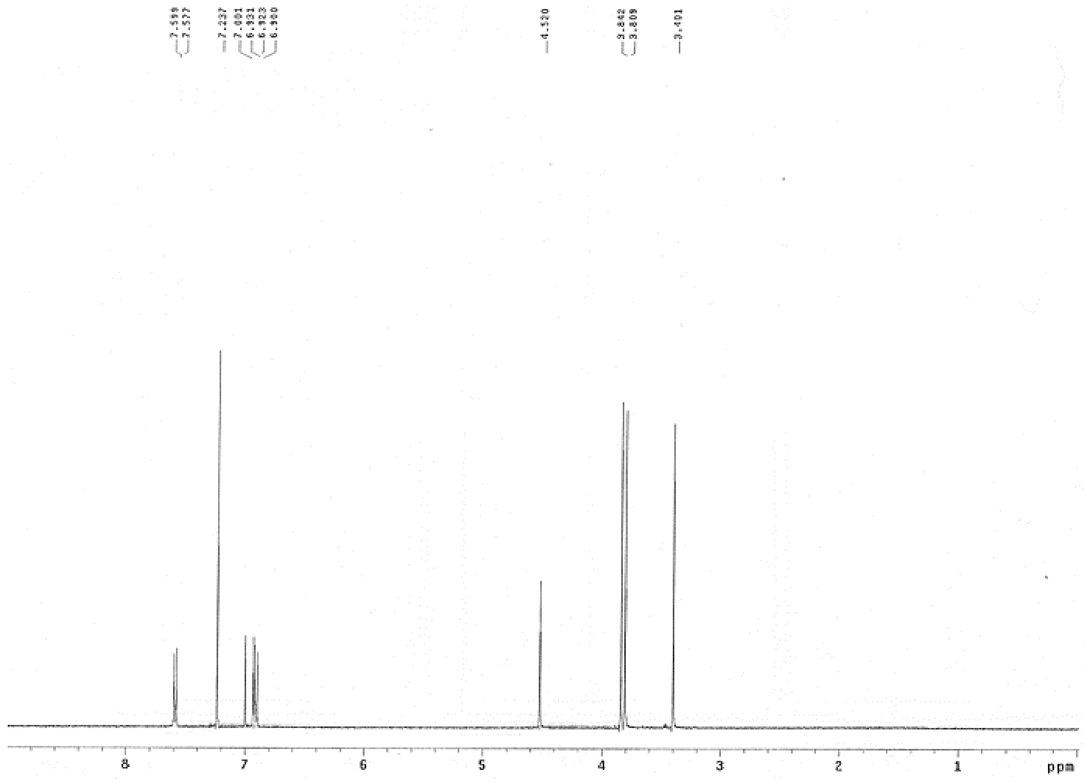

Fig. 29a: Compound **25** ^{1}H NMR spectra.

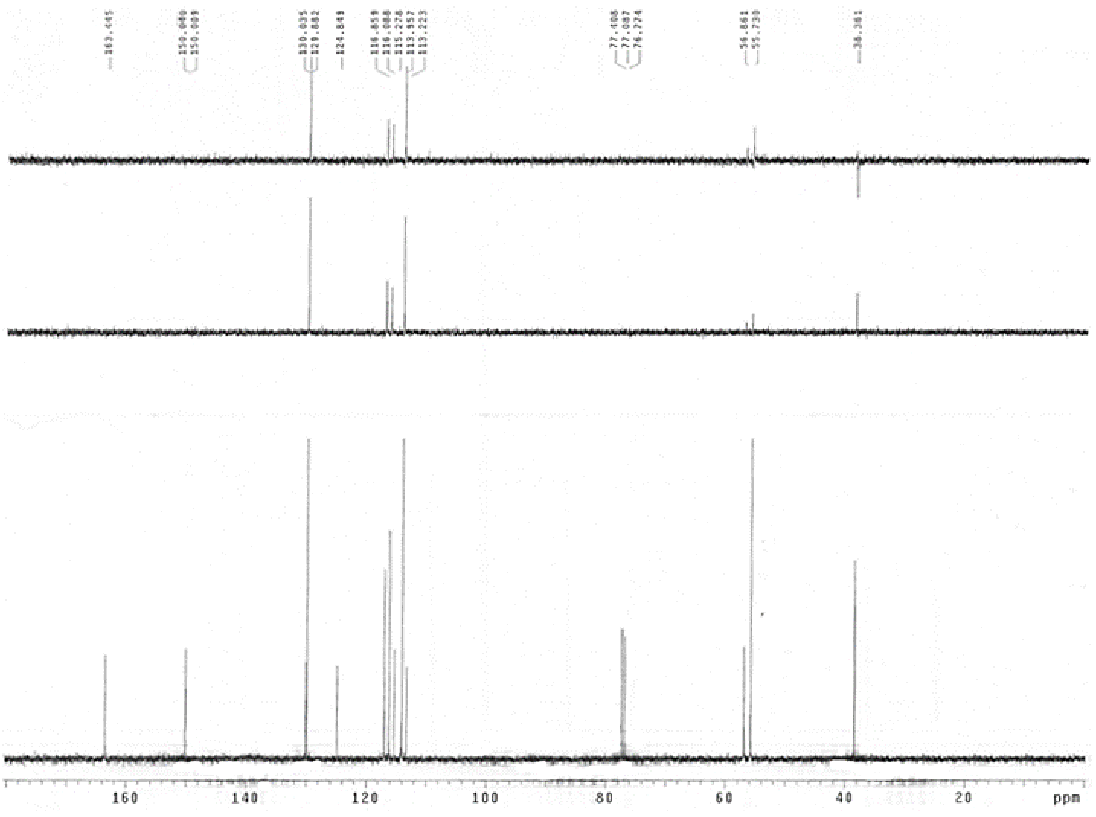

Fig. 29b: Compound **25** ^{13}C NMR spectra.

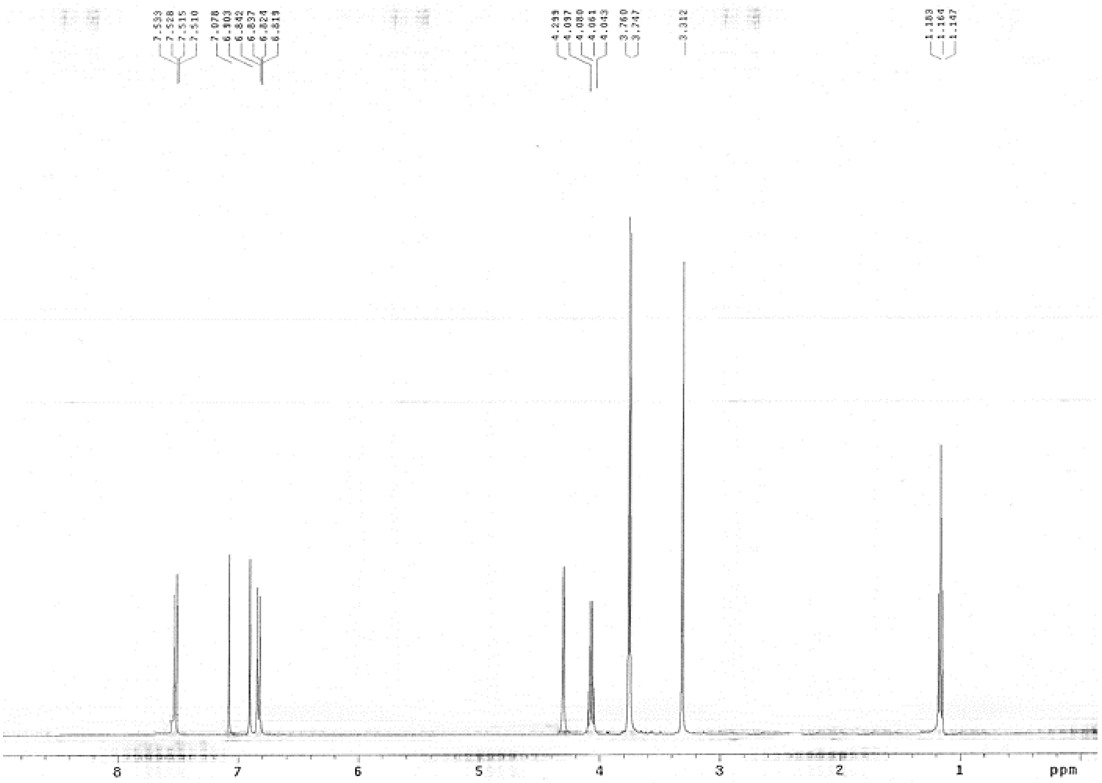

Fig. 30a: Compound **26** ^{1}H NMR spectra.

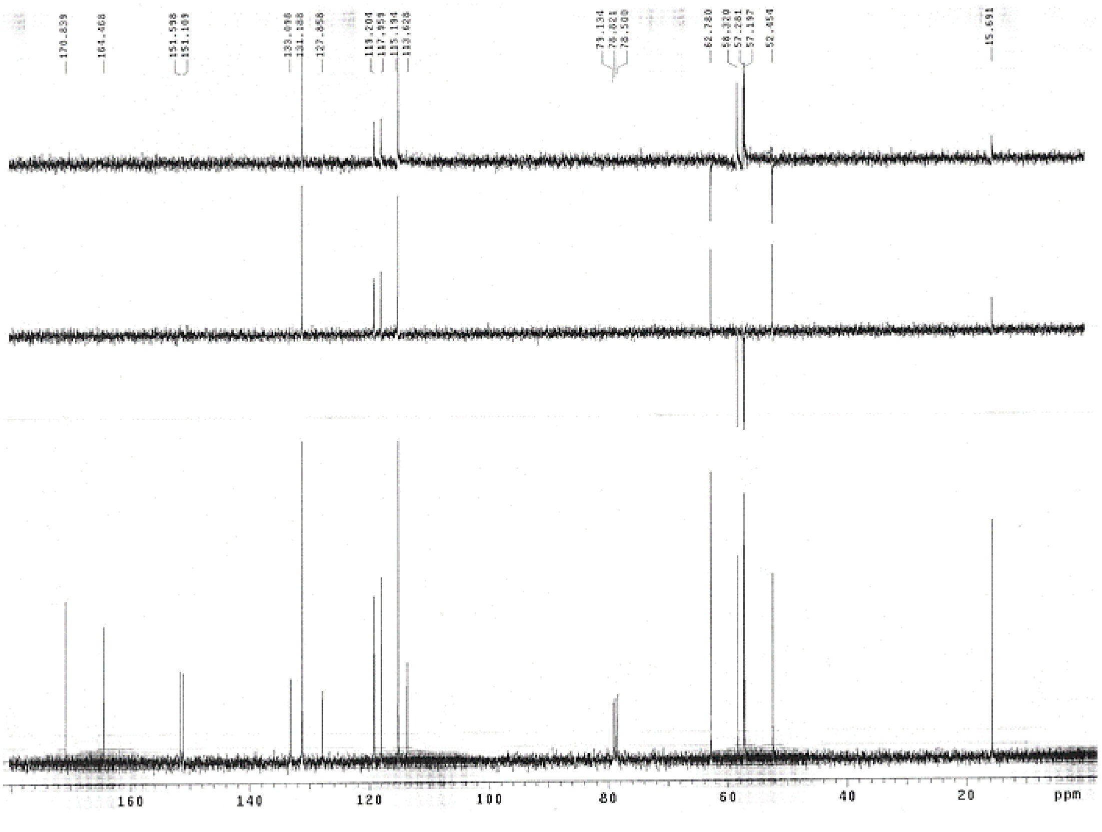

Fig. 30b: Compound **26** ^{13}C NMR spectra.

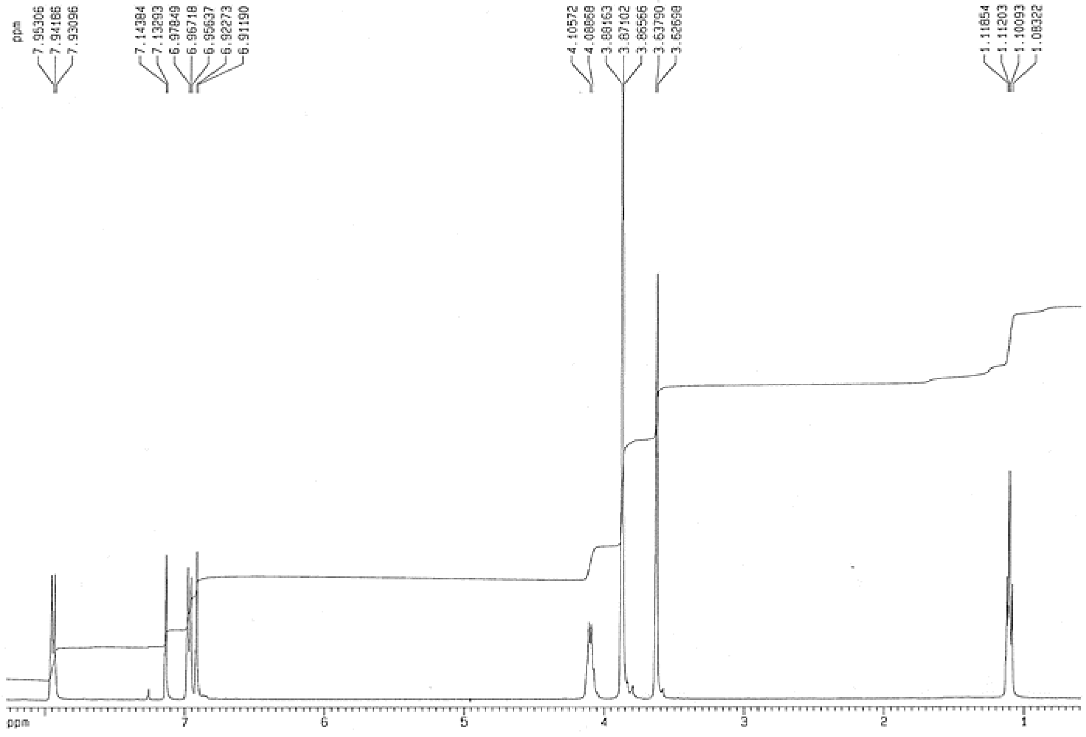

Fig. 31a: Compound **27** ^{1}H NMR spectra.

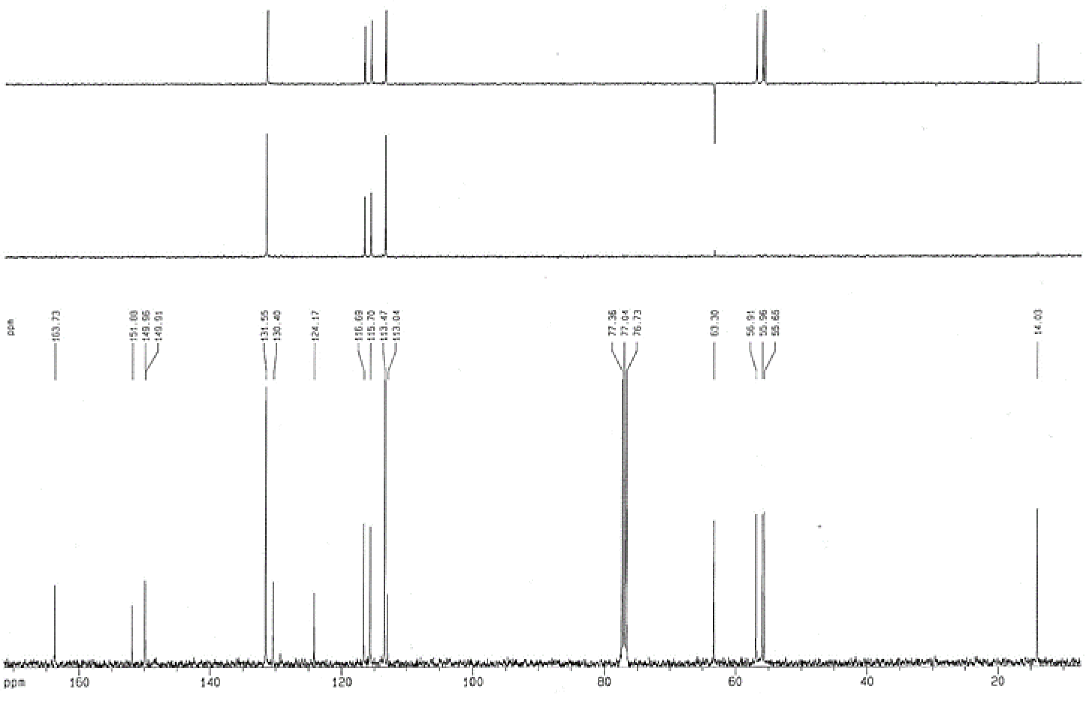

Fig. 31b: Compound **27** ^{13}C NMR spectra.

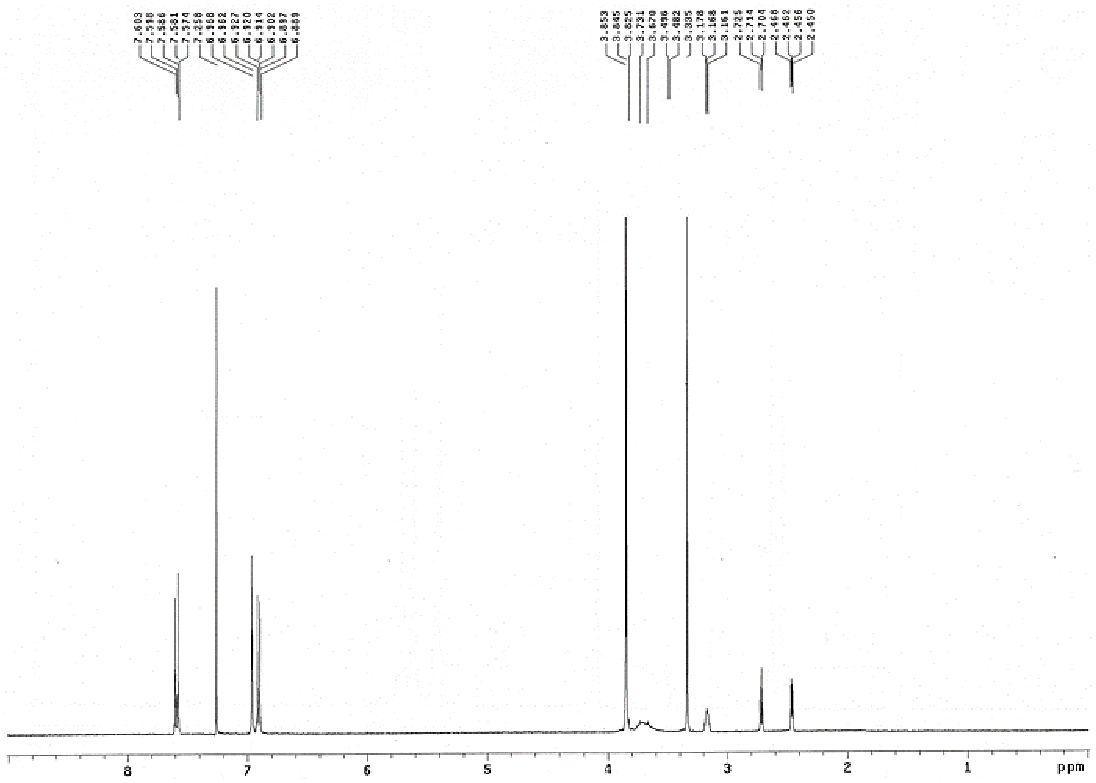

Fig. 32a: Compound **28** ^{1}H NMR spectra.

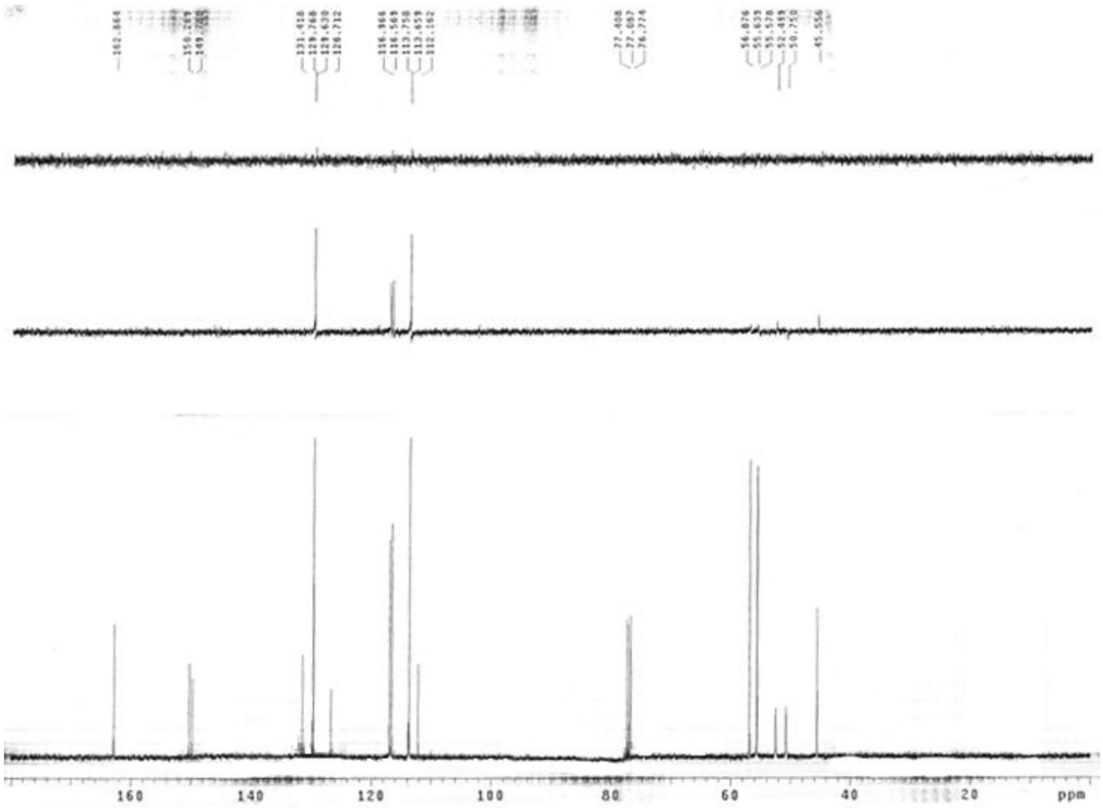

Fig. 32b: Compound **28** ^{13}C NMR spectra.

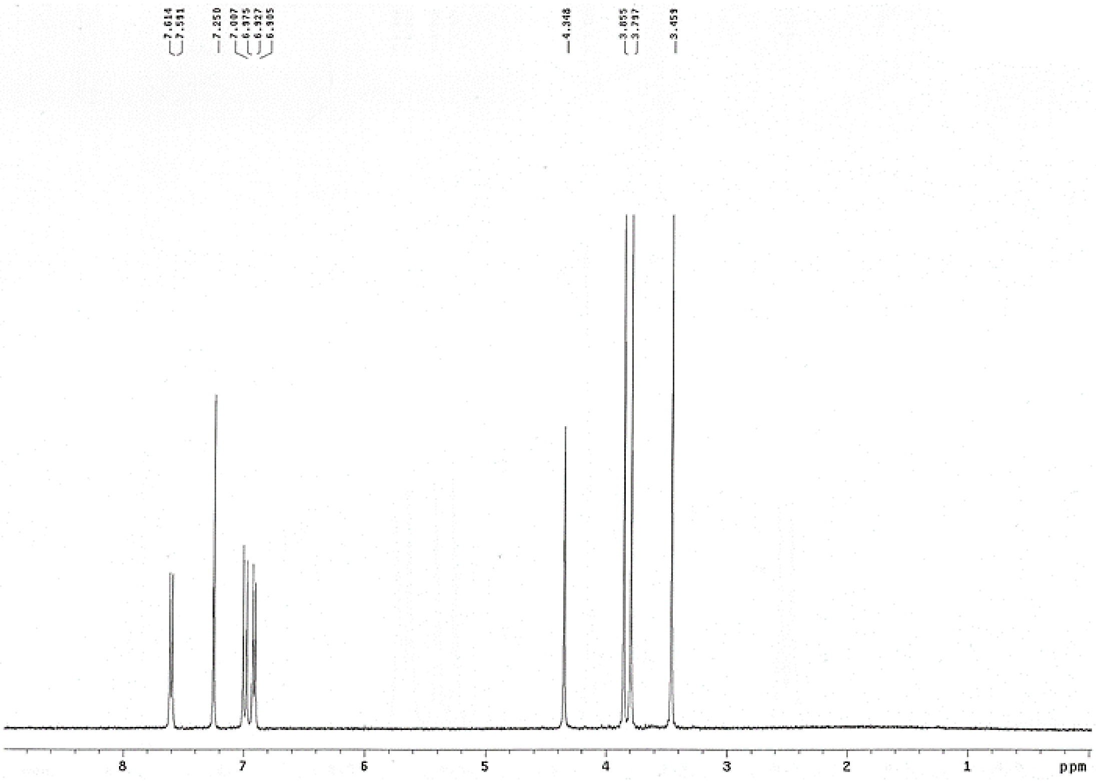

Fig. 33a: Compound **29** ^{1}H NMR spectra.

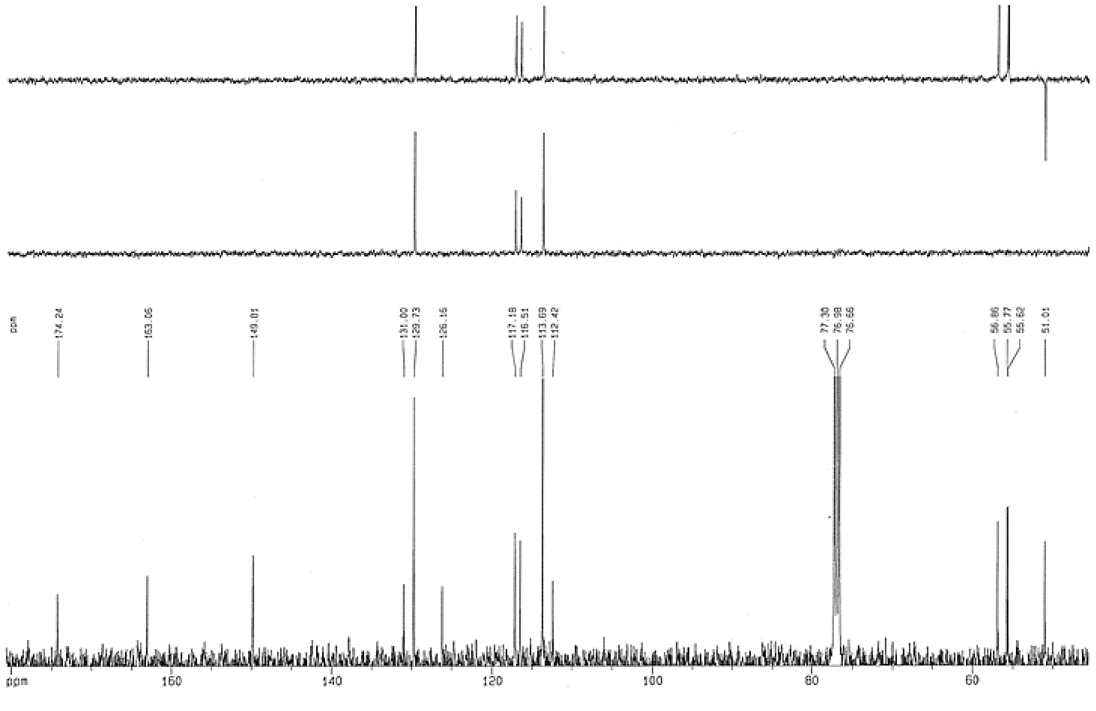

Fig. 33b: Compound **29** ^{13}C NMR spectra.

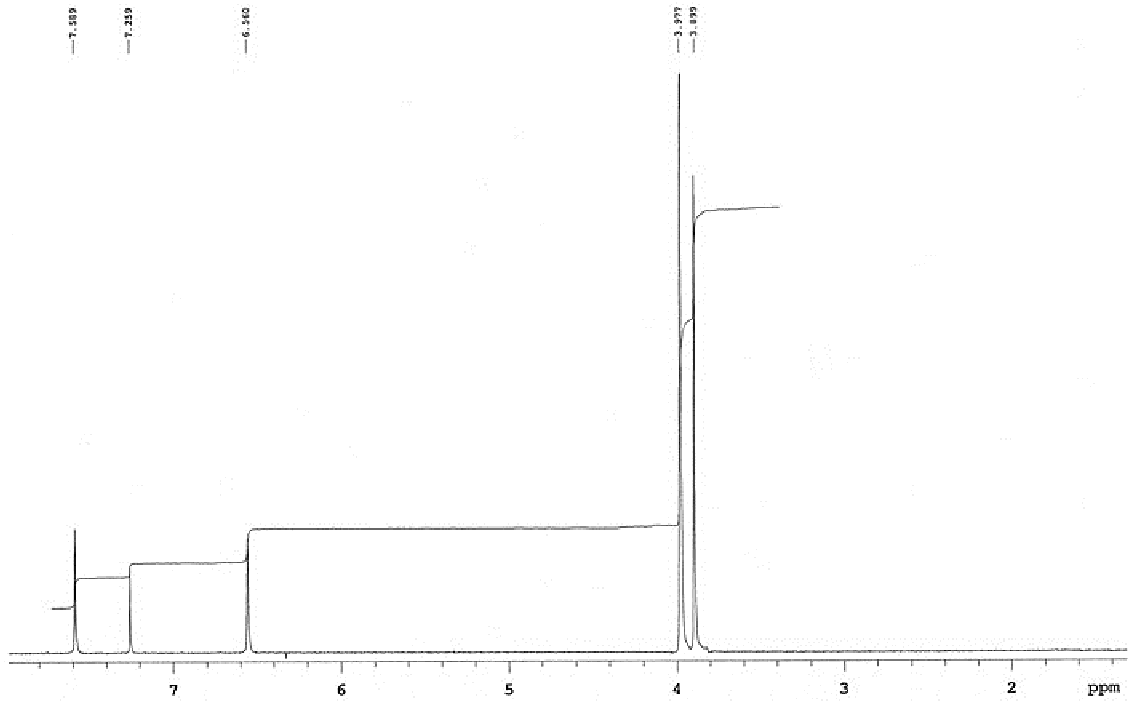

Fig. 34a: Compound **30** ^{1}H NMR spectra.

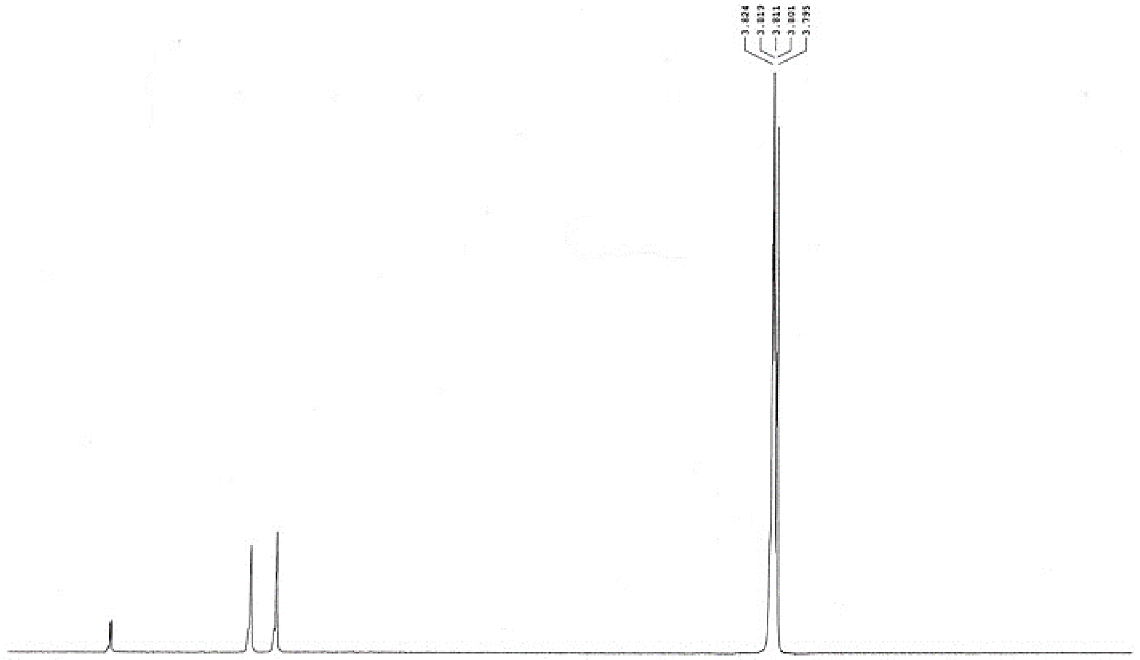

Fig. 35a: Compound **31** ^{1}H NMR spectra.

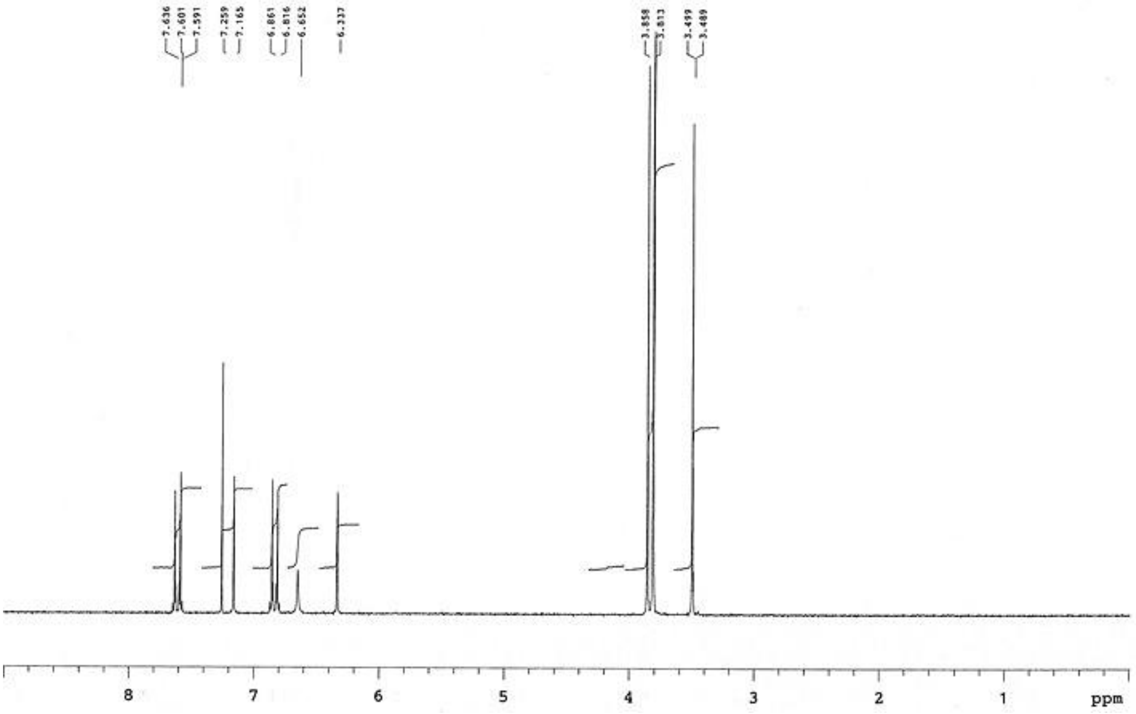

Fig. 36a: Compound **32** ^{1}H NMR spectra.

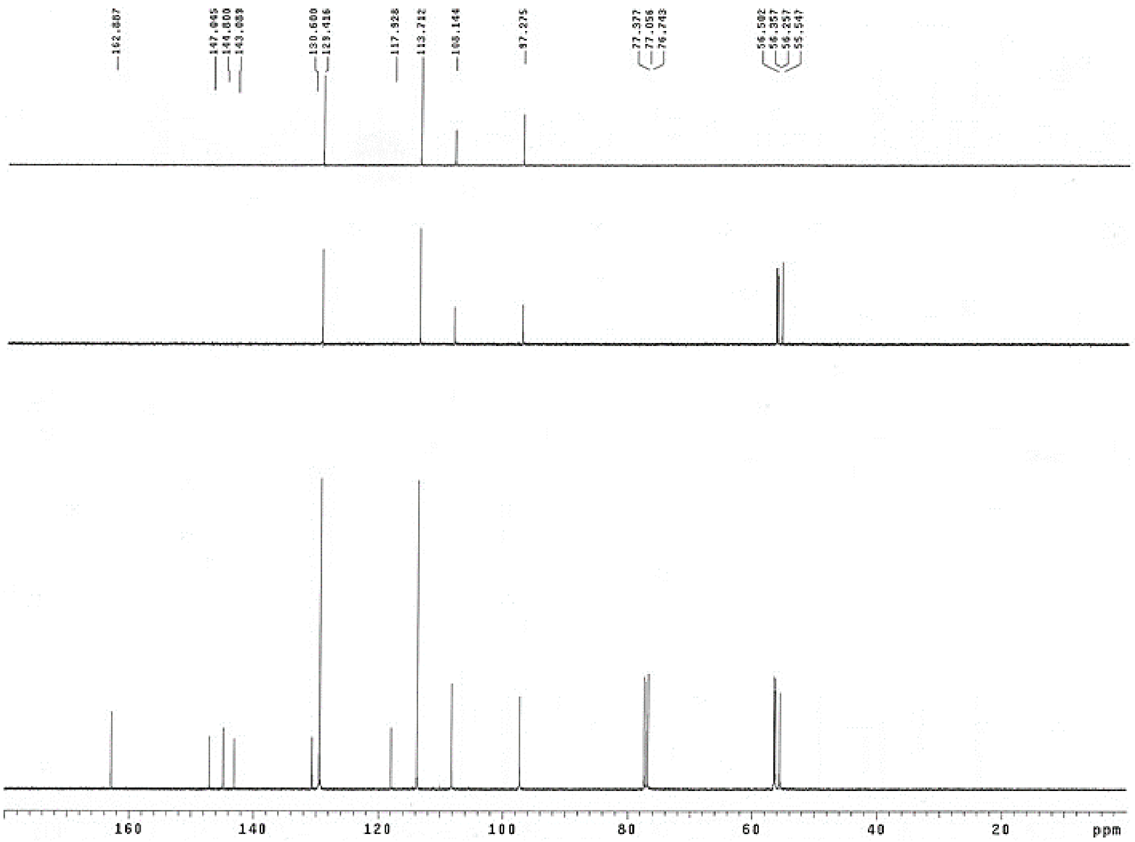

Fig. 36b: Compound **32** ^{13}C NMR spectra.

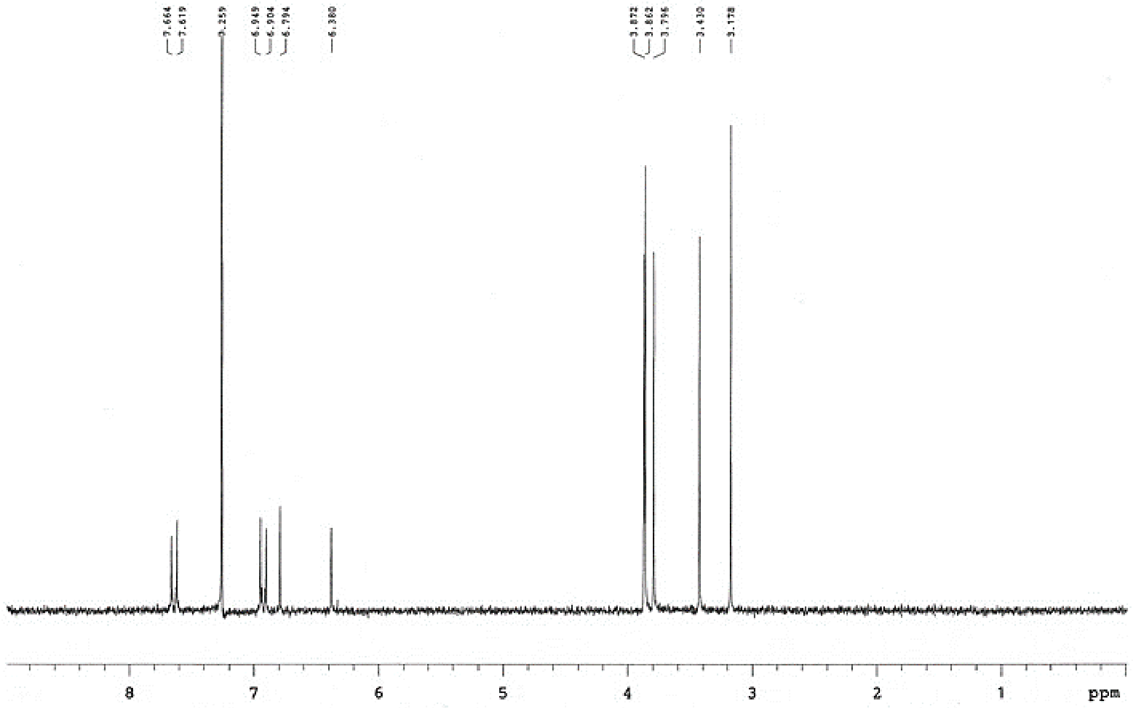

Fig. 37a: Compound **33** ^{1}H NMR spectra.

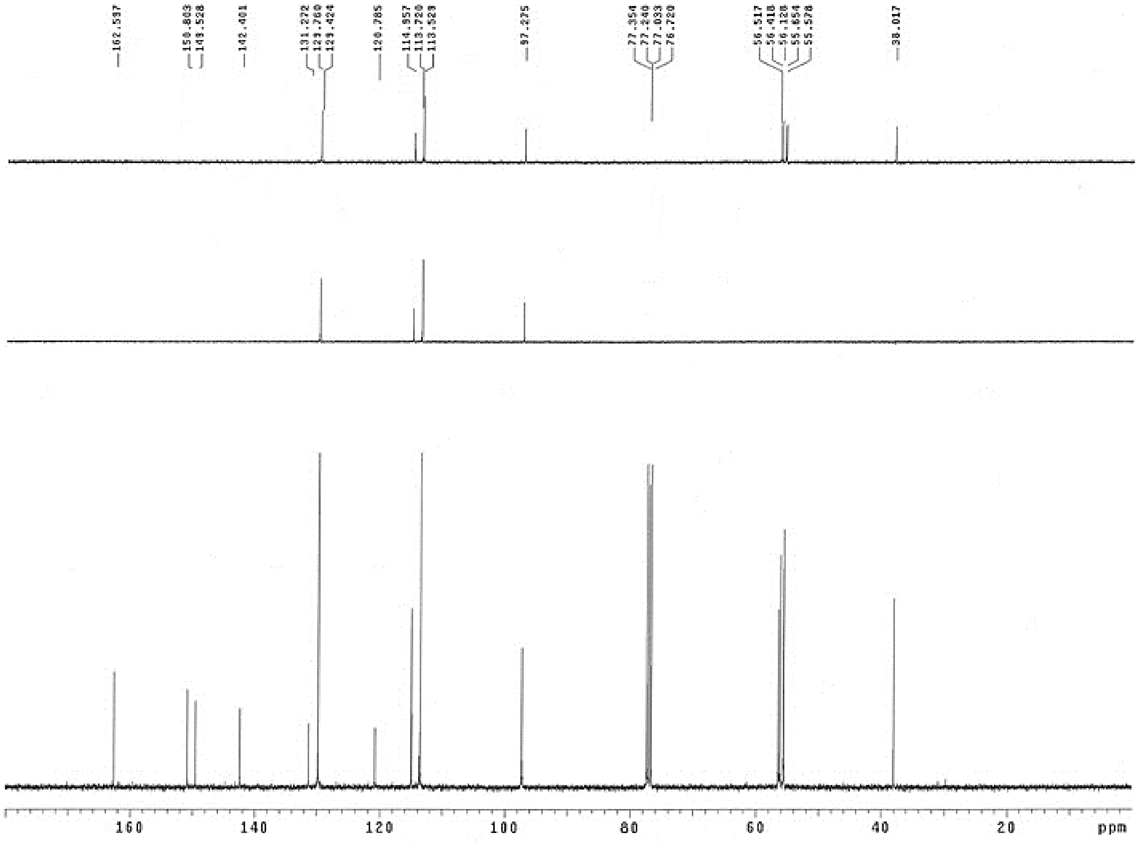

Fig. 37b: Compound **33** ^{13}C NMR spectra.

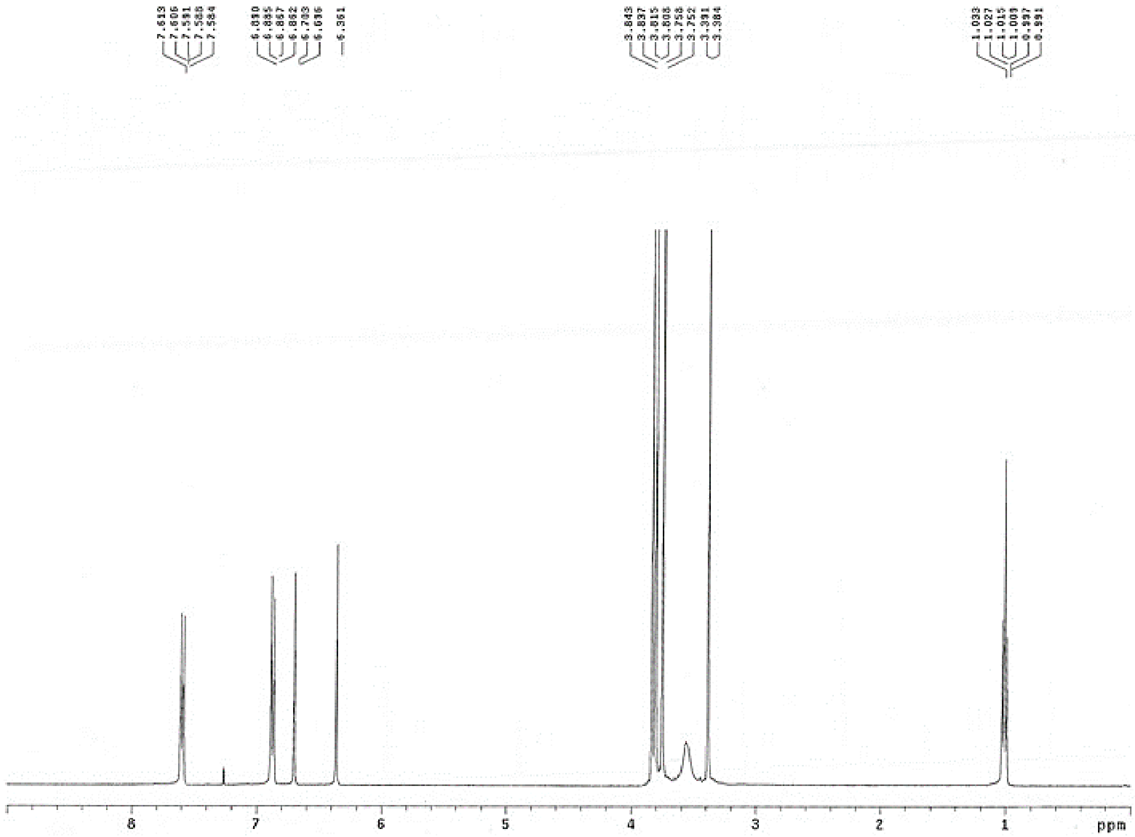

Fig. 38a: Compound **34** ^{1}H NMR spectra.

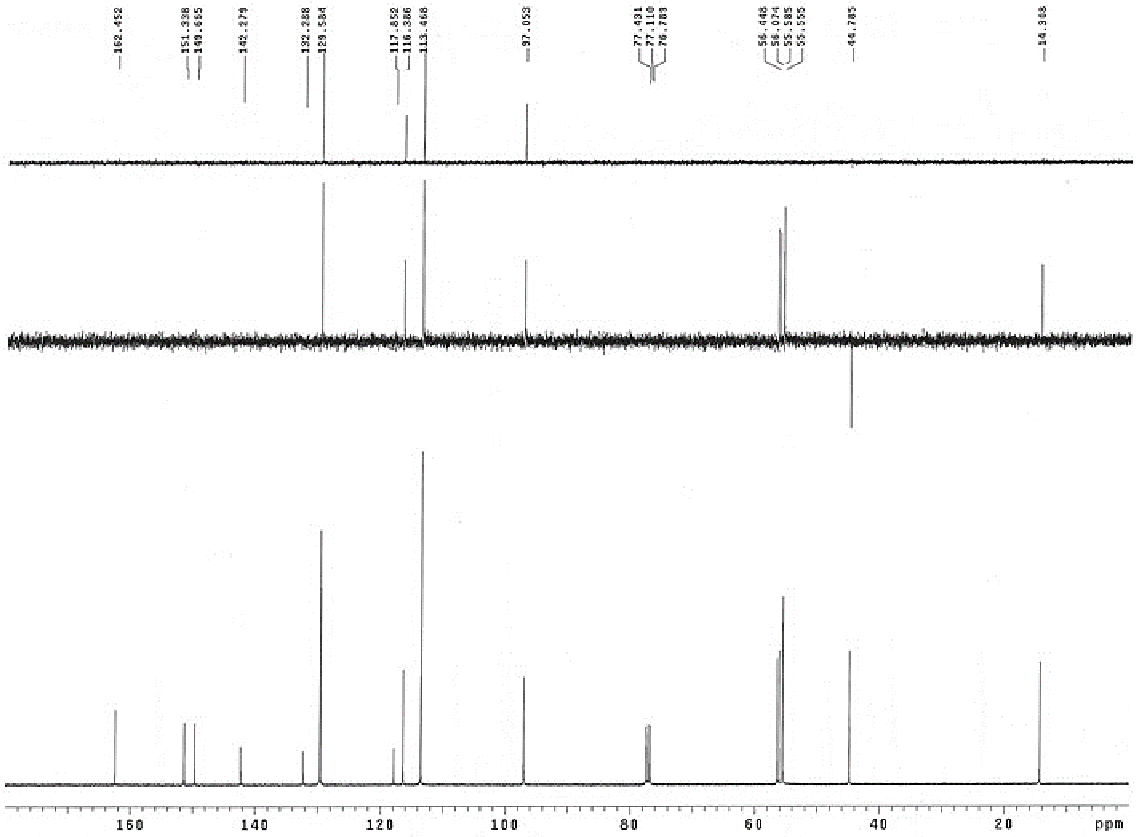

Fig. 38b: Compound **34** ^{13}C NMR spectra.

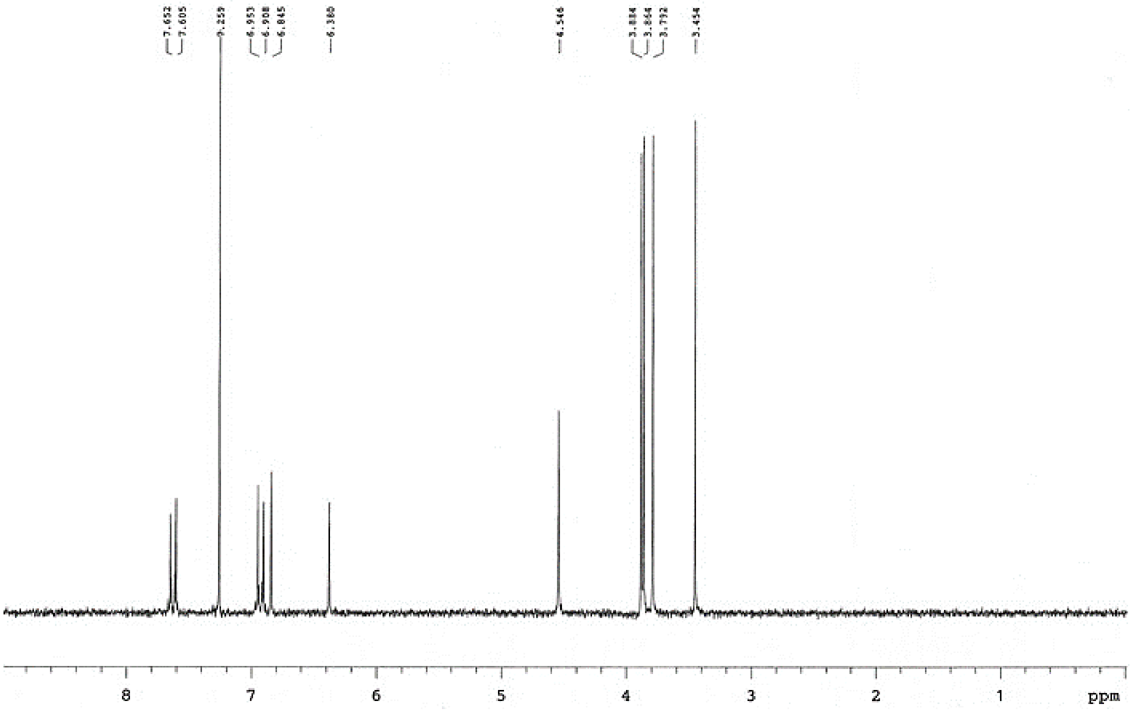

Fig. 39a: Compound **35** ^{1}H NMR spectra.

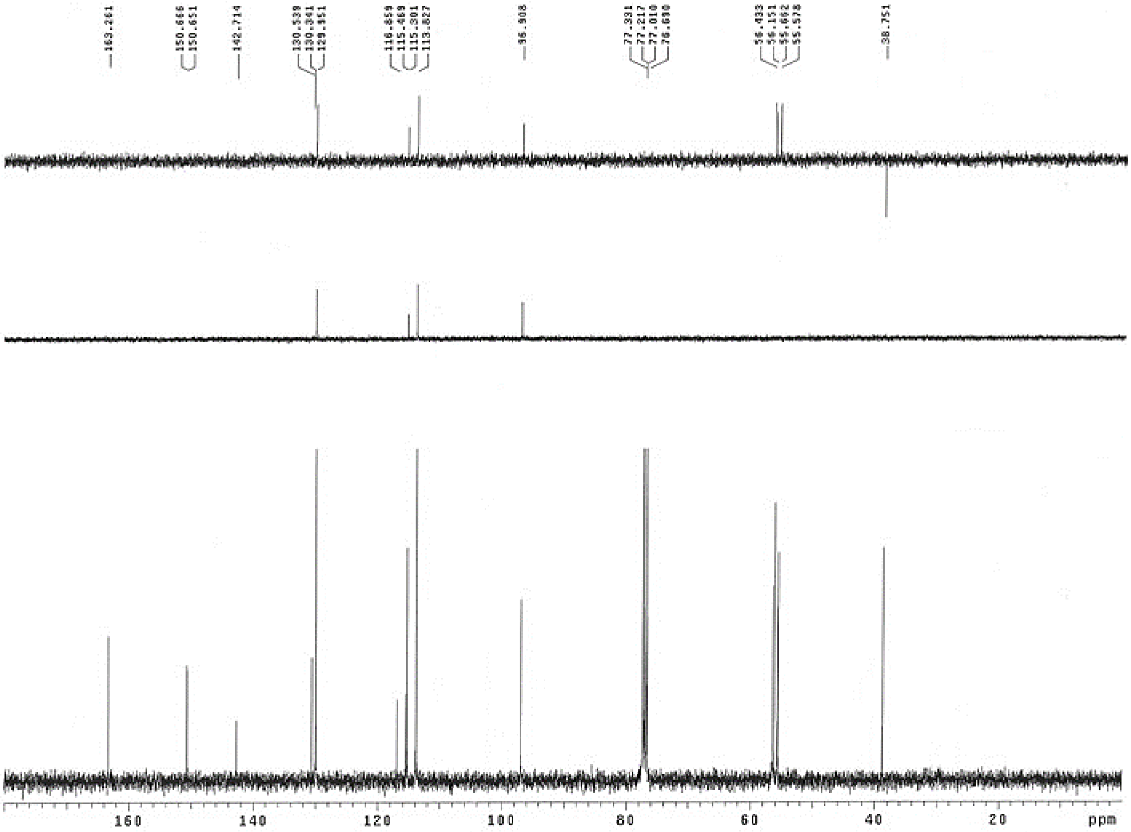

Fig. 39b: Compound **35** ^{13}C NMR spectra.

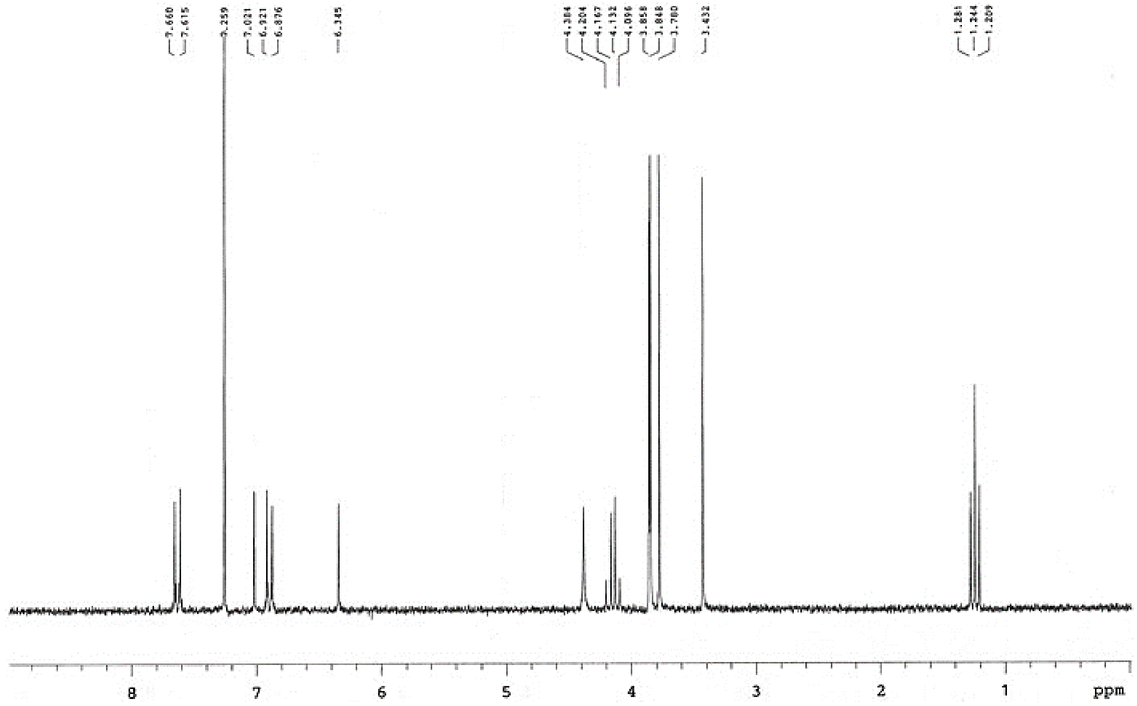

Fig. 40a: Compound **36** ^{1}H NMR spectra.

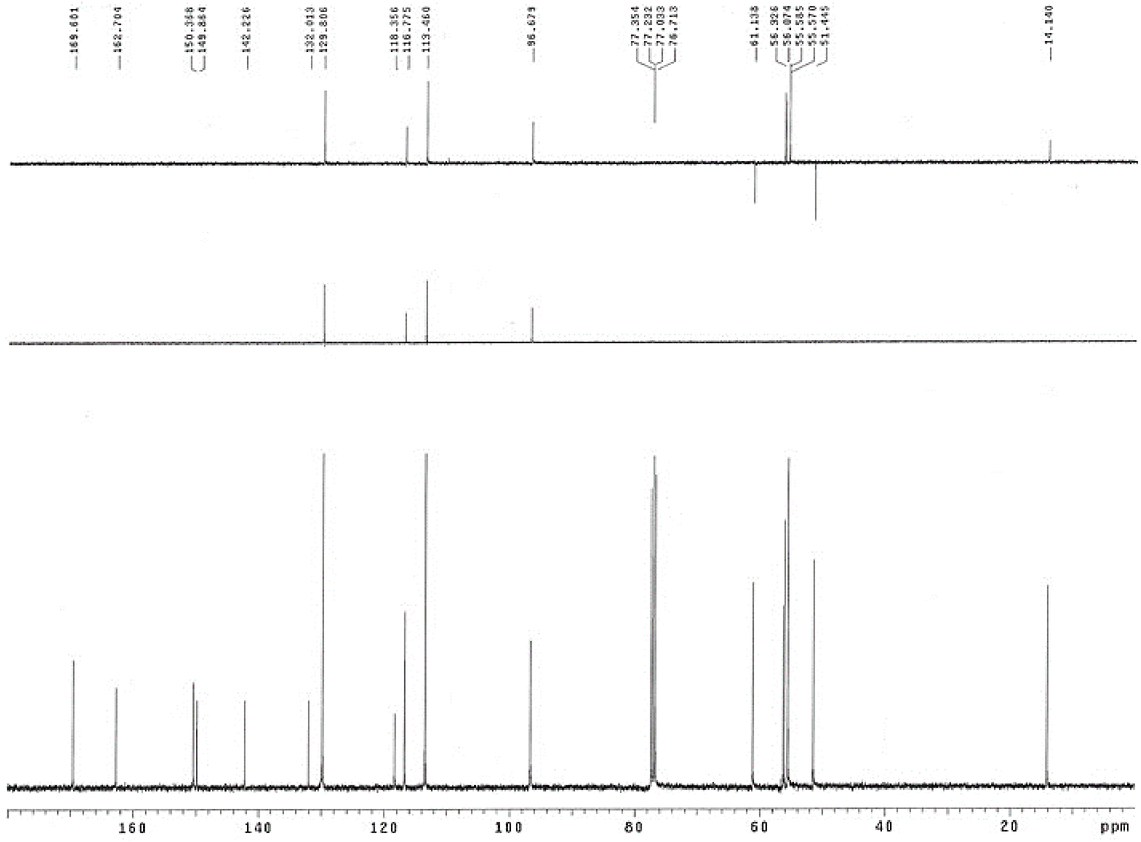

Fig. 40b: Compound **36** ^{13}C NMR spectra.

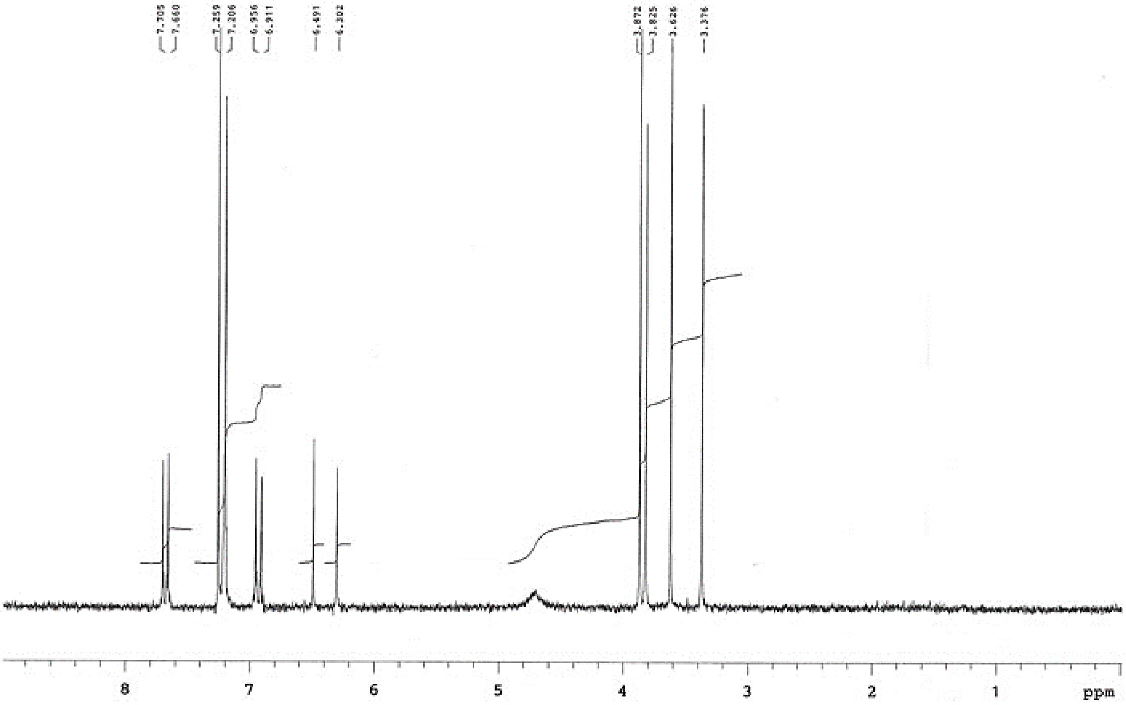

Fig. 41a: Compound **37** ^{1}H NMR spectra.

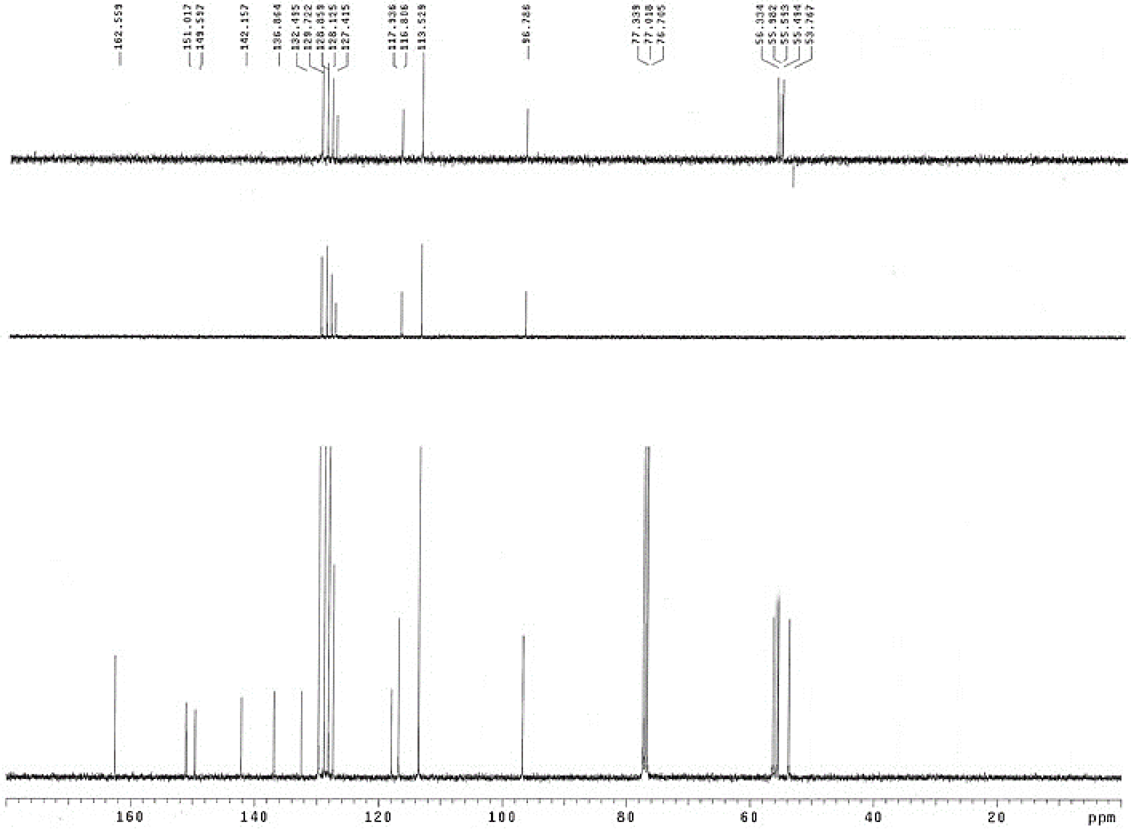

Fig. 41b: Compound **37** ^{13}C NMR spectra.
